# Synthesis of enantiomerically pure (2*S*,3*S*)-5,5,5-trifluoroisoleucine and (2*R*,3*S*)-5,5,5-trifluoro-*allo*-isoleucine

**DOI:** 10.3762/bjoc.9.236

**Published:** 2013-10-02

**Authors:** Holger Erdbrink, Elisabeth K Nyakatura, Susanne Huhmann, Ulla I M Gerling, Dieter Lentz, Beate Koksch, Constantin Czekelius

**Affiliations:** 1 Department of Chemistry and Biochemistry, Freie Universität Berlin, Takustr. 3, 14195 Berlin, Germany

**Keywords:** amino acids, CD-spectroscopy, fluorine, helix propensity, organo-fluorine, trifluoroisoleucine

## Abstract

A practical route for the stereoselective synthesis of (2*S*,3*S*)-5,5,5-trifluoroisoleucine (L-5-F_3_Ile) and (2*R*,3*S*)-5,5,5-trifluoro-*allo*-isoleucine (D-5-F_3_-*allo*-Ile) was developed. The hydrophobicity of L-5-F_3_Ile was examined and it was incorporated into a model peptide via solid phase peptide synthesis to determine its α-helix propensity. The α-helix propensity of 5-F_3_Ile is significantly lower than Ile, but surprisingly high when compared with 4’-F_3_Ile.

## Introduction

Due to the unique physicochemical properties of fluorine, namely its small size, extremely low polarizability and the strongest inductive effect among all chemical elements [1], fluorine substitution has become a powerful tool for modulating the properties of pharmaceuticals and biologically active compounds. In this respect, the incorporation of amino acids with fluorinated side chains has been established as an efficient strategy to alter distinct properties of peptides and proteins, such as hydrophobicity, acidity/basicity, and conformation [2].

Numerous studies have focused on the incorporation of fluorinated aliphatic amino acids in helical folds [3–7], even though their intrinsic tendency to adopt this secondary structure (i.e. their α-helix propensity) was shown to be considerably reduced when compared to their canonical analogues [8–9]. Thus, if enhanced thermal stabilities of helical assemblies containing aliphatic fluorinated building blocks have been observed, this stability was mainly attributed to a higher hydrophobicity or the formation of a fluorous core [2,10]. Tirrell et al. found that the biological function of the helical GCN4 transcription factor can be retained, when utilizing racemic mixtures of 5,5,5-trifluoroisoleucine (5-F_3_Ile) as isoleucine surrogates for protein synthesis in *Escherichia coli* [11–12]. Moreover, studying the impact of global Ile substitution with 5-F_3_Ile in comparison to the substitution of Val by 4,4,4-trifluorovaline (4-F_3_Val) through protein expression, they showed that the replacement of the δ-CH_3_ group of Ile by CF_3_ resulted in an approximately eight-fold higher thermal stability of the respective GCN4 analogue than the replacement of the γ-CH_3_ group of Val by CF_3_ [12]. This finding was explained by the substantial loss of side-chain entropy of Val due to steric restriction between the significantly larger γ-CF_3_ group and the helix. In agreement with these findings, we previously showed that the replacement of a CH_3_ group by a CF_3_ substituent in close proximity to the α-carbon of aliphatic amino acids dramatically reduces their α-helix propensity [13].

Since isolated α-helices are only marginally stable in solution, they are often stabilized in proteins by being wound around each other in a superhelix, a so-called coiled-coil arrangement, where hydrophobic side chain interactions are maximized within a helical interface. Coiled-coil structures are based on a (pseudo-) repetitive sequence (abcdefg)*_n_*, the so-called heptad repeat, in which hydrophobic side chains are primarily located at the a- and d-positions on one side of the helix, while most of the other positions are hydrophilic or charged [2,14]. In a parallel helix alignment of coiled-coils, two fundamentally different packing geometries occur at hydrophobic core positions a and d. The α−β bond vector of the amino acid side chain can either point out of the hydrophobic core, termed *parallel packing*, or into the core and thus directly towards the neighboring helix (*perpendicular packing*). As a consequence, hydrophobic β-branched amino acids (Ile or Val) are the most stabilizing amino acids in parallel packing arrangements, because they project the hydrocarbon side chain from the β-carbon atom directly into the helical interface, whereas perpendicular packing precludes β-branched residues from occupying these sites and Leu is favored [14–15]. Therefore, the preparation of enantiomerically pure fluorinated isoleucine analogues with retained α-helix propensity is of general interest for site-specific modification of coiled-coil positions in parallel packing arrangements, especially when solid phase peptide synthesis is employed.

Herein, we report a flexible approach to enantiomerically pure (2*S*,3*S*)-5,5,5-trifluoroisoleucine (L-5-F_3_Ile) and (2*R*,3*S*)-5,5,5-trifluoro-*allo*-isoleucine (D-5-F_3_-*allo*-Ile). Since the relationship of side chain hydrophobicity and α-helix propensity is of crucial importance for the overall stability of helical assemblies, these two properties were examined for L-5-F_3_Ile, as one of the possible fluorinated analogues of proteinogenic isoleucine.

## Results and Discussion

### Amino acid synthesis

**Scheme 1:**
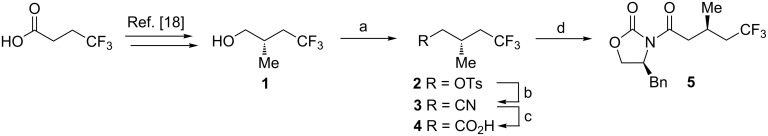
Synthesis of optically active *N*-acyloxazolidinone **5** from 4,4,4-trifluorobutanoic acid. Conditions: (a) TsCl, DMAP (cat.), pyridine, 0 °C to rt, 12 h, 79%; (b) NaCN, NaI (cat.), DMSO, 60 °C, 2.5 h, 85%; (c) HCl (conc.), reflux, 2.5 h, 80%; (d) NEt_3_, PivCl, THF, −78 °C to 0 °C, 90 min, then *n*-BuLi, (*S*)-4-benzyloxazolidin-2-one, THF, −78 °C to rt, overnight, 84%.

We have recently described a new method for the conjugate trifluoromethylation of α,β-unsaturated acyloxazolidinones [13]. Using this approach, all four diastereoisomers of *N*-Boc-protected 4,4,4-trifluorovaline as well as (2*S*,3*S*)-4,4,4-trifluoroisoleucine were prepared from the corresponding products in enantiomerically pure form via diastereoselective auxiliary-induced amination. It was found that both diastereoisomers of trifluorovaline and trifluoroisoleucine show extremely low α-helix propensities compared to their non-fluorinated analogues valine (Val) and isoleucine (Ile), which we attribute to steric clashes of the larger γ-CF_3_ group with the helix backbone [13]. We wondered whether the replacement of the δ-CH_3_ group of Ile by CF_3_ might retain α-helix propensity. In light of the fact that 5,5,5-trifluoroisoleucine has mostly been synthesized and incorporated into peptides as diastereomeric mixtures so far [11–12,16], we decided to extended our previous approach towards the synthesis of (2*S*,3*S*)-5,5,5-trifluoroisoleucine. For this, we envisioned acyloxazolidinone **5** as an intermediate for the synthesis of enantiomerically pure L-5,5,5-trifluoroisoleucine [13,17]. For the synthesis of this building block we started from enantiomerically pure alcohol **1**, which was prepared using a modified protocol reported by Wang and Resnick from commercially available 4,4,4-trifluorobutanoic acid by diastereoselective enolate alkylation followed by reduction with lithium borohydride (Scheme 1) [18].

**Scheme 2:**
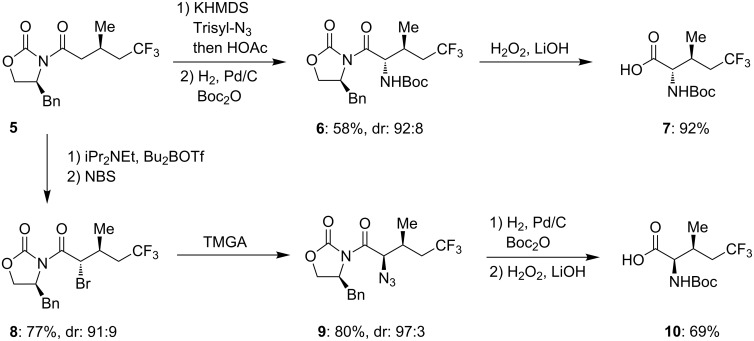
Synthesis of enantiomerically pure (2*S*,3*S*)-5-F_3_Ile and (2*R*,3*S*)-5-F_3_-*allo*-Ile. TMGA = 1,1,3,3-tetramethylguanidinium azide, Trisyl-N_3_ = 2,4,6-triisopropylbenzene-sulfonyl azide.

Fluorinated alcohol **1** was transformed into the corresponding tosylate ester **2** and subsequently reacted with sodium cyanide to afford nitrile **3**. Acid hydrolysis of **3** provided the enantiomerically pure carboxylic acid (*R*)-**4** [19], which was then coupled to the oxazolidinone via the mixed anhydride (Scheme 1). With *N*-acyloxazolidinone **5** in hand, the optically active fluorinated α-amino acids (L-5,5,5-trifluoroisoleucine **7** and D-5,5,5-trifluoro-*allo*-isoleucine **10**) were synthesized by stereoselective, auxiliary-induced amination following procedures reported by Evans and coworkers (Scheme 2) [17,20–24]. For this, acyloxazolidinone **5** was transformed into the α-azido derivative which upon reduction and auxiliary removal gave the *N*-Boc protected L-amino acid **7** (L-5-F_3_Ile). Likewise, the corresponding D-amino acid **10** (D-5-F_3_-*allo*-Ile) was prepared by α-bromination followed by nucleophilic azide displacement under stereochemical inversion (Scheme 2).

The absolute configuration of the amino acids was confirmed by crystal structure determination of intermediate **6**, based on the known auxiliary configuration (see Supporting Information File 1).

### Hydrophobicity of L-5-F_3_-Ile

We investigated the relationship between side chain volume and hydrophobicity of L-5-F_3_Ile. Since size and hydrophobicity are known to be essential factors for secondary structure formation, we compared 5-F_3_Ile with previously studied (2*S*,3*S*)-4’-F_3_Ile [13], as well as with (*S*)-5,5,5,5’,5’,5’-hexafluoroleucine (F_6_Leu), which was prepared according to procedures reported by Keese and coworkers [25].

By plotting their side chain van der Waals volume versus their retention time from an RP-HPLC experiment, we previously investigated the relationship between size and hydrophobicity of various fluorinated and non-fluorinated amino acids [26]. In this experiment the non-polar phase of a reversed-phase column serves as a mimic of a biological membrane or the kind of hydrophobic interactions, that would be present in hydrophobic cores of proteins and in ligand–receptor binding [27]. We extended these initial studies by 2-aminoheptanoic acid (Aha) as an amino acid with an unbranched aliphatic side chain. The van der Waals volumes of the amino acid side chains were calculated according to Zhao et al*.* [28].

**Figure 1:**
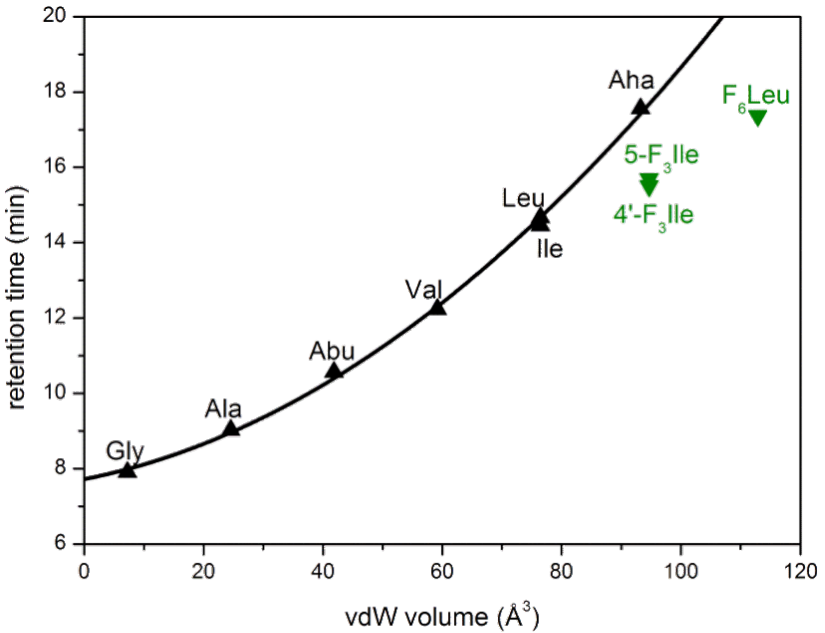
Retention times of Fmoc amino acids plotted against the van der Waals volume of their side chains. Non-ﬂuorinated amino acids are depicted as black triangles; their correlation is shown as a black line. Fluorinated amino acids are represented by green triangles.

Aha correlates very well with its smaller non-fluorinated analogues and their retention time increases non-linearly with increasing side chain volume (Figure 1). As expected, the enlargement of the aliphatic side chain results in an increase in hydrophobicity.

In agreement with previous studies that focused on other fluorinated amino acids [26], also the retention times of 5-F_3_Ile and 4’-F_3_Ile do not fit into the correlation between side chain volume and retention time (Figure 1). Although similar in size, the two fluorinated stereoisomers of Ile are less hydrophobic than Aha. F_6_Leu is similar to Aha in hydrophobicity, while exhibiting a much larger volume. In a free energy perturbation study, the hydration energy of F_6_Leu was shown to be 1.1 kcal/mol higher than that of leucine [29]. This, together with our previous and new findings, suggests that there are two factors determining the overall hydrophobicity of fluorinated amino acids [26]. On one hand, substitutions of hydrogen by fluorine increase the solvent accessible surface area and thus lead to an increase in hydration energy. On the other hand, the C–F bond is more polarized than the C–H bond, and electrostatic interactions of the fluorinated group with the solvent are energetically more favored. As a consequence, fluoroalkyl side chains possess two seemingly contrary physicochemical properties, hydrophobicity and polarity, and the combination of both leaves fluorinated amino acids to be less hydrophobic than their surface area would suggest.

### α-Helix propensity of L-5-F_3_Ile

In general, fluorination of amino acids leads to a dramatic decrease in helix propensity [8–9,13]. As the extreme of this effect, we previously reported the complete loss of helix propensity when the β-methyl group in β-branched hydrophobic amino acids is replaced by a CF_3_-substituent [13]. We now investigated the α-helix propensity of 5-F_3_Ile according to methods established by Cheng et al., who showed that when an amino acid of interest is incorporated into an α-helical polyalanine model peptide (KX), its α-helix propensity can be calculated from circular dichroism (CD) spectroscopy [8–9]. Therefore, 5-F_3_Ile was converted into its Fmoc analogue and subsequently used in solid-phase synthesis of K-5-F_3_Ile applying standard Fmoc-based chemistry (see Supporting Information File 1) [30]. The α-helix propensity [ω] was calculated from CD data (Table 1).

**Table 1:** Ellipticity [Θ] at 222 nm was taken from normalized CD data. Fraction helix [f_helix_] and helix propensities [ω] were calculated from [Θ_222 nm_] applying a modified Lifson–Roig theory [31–33].

peptide	[Θ_222 nm_]	*f*_helix_	*ω*

K-Ile	–13813 ± 156	0.40 ± 0.01	0.52 ± 0.05
K-5-F_3_Ile	–10776 ± 216	0.31 ± 0.01	0.26 ± 0.03
K-4’-F_3_Ile [13]	–3602 ± 130	0.10 ± 0.01	0

**Figure 2:**
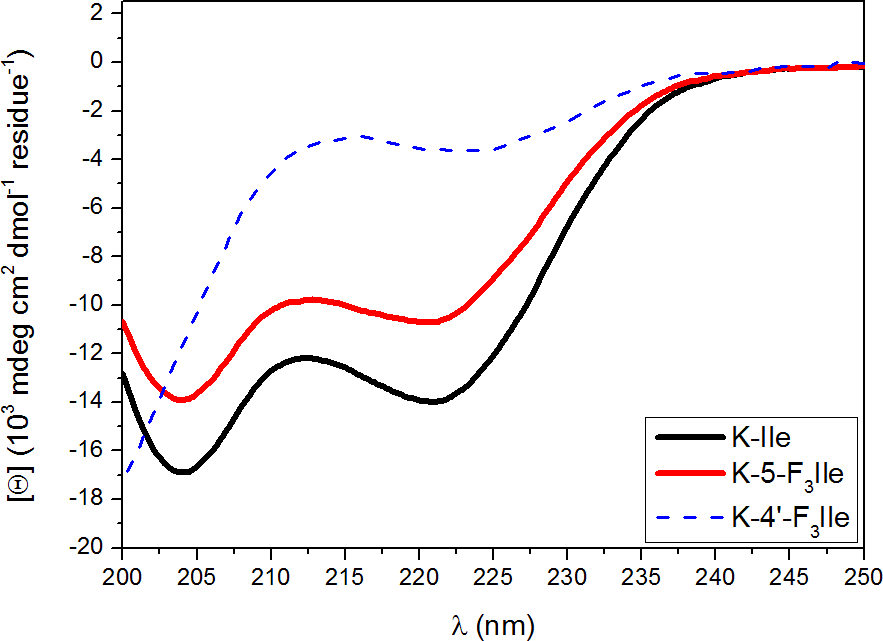
CD spectra of K-5-F_3_Ile and K-Ile peptides (KX: Ac-YGGKAAAAKA**X**AAKAAAAK-NH_2_). The K-4’-F_3_Ile spectrum [13] is shown for comparison. Spectra were recorded at pH 7 in 1 mM phosphate, borate, and citrate buffer with 1 M NaCl at 0 °C. Depicted spectra are normalized and represent the mean of three independent measurements at three different concentrations (80 µM, 50 µM, and 30 µM).

Although the helix propensity of 5-F_3_Ile is half of that for Ile, two distinct minima at 208 nm and 222 nm in the corresponding CD spectrum clearly indicate a helical structure of the model peptide, whereas the absence of these minima in the K-4’-F_3_Ile spectrum demonstrates a complete loss of helicity in the corresponding peptide (Figure 2). The drastic decrease in helix propensity upon fluorination has been previously attributed to a possible burial of fluorocarbon side chains in the unfolded state of the model peptide [8]. The exposure of these side chains in the helical state would lead to unfavourable helix formation energetics, due to the hydrophobic nature of fluorocarbon side chains. Moreover, due to its branching on the β-carbon atom, the side chain atoms of Ile come in close proximity to the peptide backbone [34], leading to a reduced α-helicity in comparison to its unbranched analogue ([ω]_Leu_= 1.06 [8]). This effect is amplified for K-4’-F_3_Ile which carries the voluminous CF_3_ group on its β-carbon atom. The close proximity of this sterically demanding group to the peptide backbone seems to prevent the formation of α-helical structures, resulting in a helix propensity of zero [13]. Here, we show that if isoleucine’s δ-methyl group is substituted with CF_3_, the α-helix propensity of this amino acid is partially retained.

## Conclusion

We synthesized two diastereoisomers of 5,5,5-trifluoroisoleucine ((2*S*,3*S*)-5-F_3_Ile and (2*R*,3*S*)-5-F_3_-*allo*-Ile) in enantiomerically pure form. The hydrophobicity of (2*S*,3*S*)-5-F_3_Ile was shown to be increased in comparison to its proteinogenic analogue, but to a lesser extend than the surface area would suggest. The α-helix propensity of 5-F_3_Ile, though lower than that of Ile, is significantly increased in comparison to (2*S*,3*S*)-4,4,4-trifluoroisoleucine. Thus, fluorinating isoleucine’s δ-position rescues α-helix propensity, while the fluorination of isoleucine’s β-branched methyl group abolishes it. It remains to be elucidated as to what extent helix propensity affects protein stability at buried positions, e.g. within hydrophobic cores of proteins, since the reduced helix propensity may in part be attributed to unfavorable solvent interactions at exposed positions of the applied monomeric model peptide. Since hydrophobic, β-branched amino acids are the most stabilizing amino acids in parallel packing arrangements within the hydrophobic core of coiled-coils [14–15], we believe that 5-F_3_Ile demonstrates a promising building block for fluorine modifications within this folding motif.

## Supporting Information

File 1 Experimental procedures, characterization data, copies of all ^1^H, ^13^C, and ^19^F NMR spectra of all new compounds.
