# Supplementary material for: Synthesis of enantiomerically pure (2S,3S)-5,5,5-trifluoroisoleucine and (2R,3S)-5,5,5-trifluoro-allo-isoleucine
Source: Beilstein J Org Chem. 2013 Oct 2;9:2009–14. doi: 10.3762/bjoc.9.236 (PMC3817528; doi:10.3762/bjoc.9.236)
Supplement: File 1 — Experimental procedures, characterization data, copies of all 1H, 13C, and 19F NMR spectra of all new compounds. [file Beilstein_J_Org_Chem-09-2009-s001.pdf]

Supporting Information

for

Synthesis of enantiomerically pure (2*S*,3*S*)-5,5,5-trifluoroisoleucine and (2*R*,3*S*)-5,5,5-trifluoro-*allo*-isoleucine

Holger Erdbrink[§], Elisabeth K. Nyakatura[§], Susanne Huhmann, Ulla I. M. Gerling, Dieter Lentz[†], Beate Koksch* and Constantin Czekelius*

Address: Department of Chemistry and Biochemistry, Freie Universität Berlin, Takustr. 3, 14195 Berlin, Germany

Email: Beate Koksch* - beate.koksch@fu-berlin.de; Constantin Czekelius* - cczekeli@chemie.fu-berlin.de

*Corresponding author

[§]These authors contributed equally; [†]Responsible for X-ray analysis.

Experimental procedures, characterization data, copies of all ^{1}H, ^{13}C, and ^{19}F NMR spectra of all new compounds

General procedures:

All syntheses involving air- and moisture-sensitive compounds were carried out using standard Schlenk-type glassware (or in a glove box) under an atmosphere of argon. Solvents were dried with the solvent purification system MB-SPS 800 from M. Braun or predistilled according to standard laboratory methods. Unless stated otherwise all chemicals were purchased from *Acros* or *Aldrich* and used without further purification.

The following instruments were used for physical characterization of the compounds: ^{1}H, ^{13}C, and ^{19}F NMR spectra were recorded at room temperature using a Bruker AC 250, JEOL ECX 400, JEOL Eclipse 500, or Bruker Avance 3 (700 MHz). ^{1}H NMR and ^{13}C NMR: chemical shift δ are referenced against the internal solvent ($CDCl_3$, δ ^{1}H = 7.26 ppm, ^{13}C = 77.0 ppm, $(CD_3)_2CO$, δ ^{1}H = 2.05 ppm, ^{13}C = 29.84 ppm). ^{19}F NMR: chemical shift δ is given relative to $CFCl_3$ (external reference). The order of citation in parentheses is a) multiplicity (s = singlet, d = doublet, t = triplet, q = quartet, m = multiplet, br = broad), b) coupling constants, c) number of protons and d) assignment. Coupling constants (*J*) are reported in Hertz (Hz). The attributions of the chemical shifts were determined by means of COSY and HMQC. Melting points (mp) were determined using a Büchi 510 apparatus and are uncorrected. High resolution mass spectra (HRMS) were determined on an Agilent 6210 ESI-TOF MS instrument with a flow rate of 4 μL/min, spray voltage 4 kV, and the desolvation gas set to 15 psi. All other parameters were optimized for maximal abundance of $[M + H]^+$ or $[M + Na]^+$. IR spectra were recorded using a Jasco 6200 FTIR spectrometer. The following abbreviations were used to distinguish between the signals: vs: very strong, s: strong, m: medium, w: weak, br: broad. The specific rotation of optically active compounds was determined with a *241 Perkin-Elmer* polarimeter using a cell with 10.0 cm diameter and a capacity of 1 mL. Measurements were performed at room temperature using a Na-lamp with a wavelength of λ = 589.3 nm. Chloroform was used as the solvent. X-ray data sets were collected with a Smart-CCD diffractometer. For structure solution and refinement the programs of the SHELXS-97 series were used (Sheldrick, G. M. *Acta Cryst.* **1990**, *A46*, 467–473; Sheldrick, G. M. *Acta Cryst.* **2008**, *A64*, 112–122). For the visualization the programs Diamond and Mercury were used. CCDC 943884 contains the supplementary crystallographic data for this paper. These data can be obtained free of charge from the Cambridge Crystallographic Data Centre via http://www.ccdc.cam.ac.uk/ data_request/cif. All reactions were monitored by thin-layer chromatography (TLC) using Merck silica gel 60 F_{254} (0.25 mm thickness). Flash chromatography was carried out using Merck silica gel 60 (0.040−0.063 mm). Preparative TLC was carried out using Merck PLC silica gel 60, F_{254}, 2 mm.

Peptide synthesis:

Peptides were synthesized on a Multi-SyntechSyro XP peptide synthesizer (MultisynTech GmbH, Witten, Germany) applying standard Fmoc strategy and using TBTU/HOBt/DIEA activation. A NovaSyn®TGR-resin was preloaded with the first amino acid Fmoc-Lys(Boc)-OH (0.240 mmol/g, 0.025 mmol scale). The fluorinated amino acid at the guest position Xaa was

coupled manually using 1.20 equiv amino acid and HOAt/DIC activation. The following one was also coupled manually using 10.0 equiv amino acid and HOBt/DIC activation. Fmoc deprotection was achieved by treatment with 2% piperidine and 2% DBU (4 × 5 min). For capping with acetic anhydride, a solution of Ac_2O (10% (v/v)), and DIEA (10% (v/v)) in DMF (3.0 mL) was applied (3 × 10 min). Peptides were cleaved from the resin by treatment with 2.0 mL of a solution containing triisopropylsilane (2.5% (v/v)), water (2.5% (v/v)), and TFA (95% (v/v)), purified by reversed-phase preparative HPLC, and identified by ESI–ToF MS using an Agilent 6210 ESIToFLC–MS spectrometer (Agilent Technologies Inc., Santa Clara, CA, USA). Analytical HPLC was performed to verify purity on a LaChrom-HPLC-system containing an Interface L-7000, two pumps L-7100, diode array detector L-7450 and an autosampler L-7200.

**Peptide**	**Peptide sequence**	**$[M + 2H]^{2+}$ calculated**	**$[M + 2H]^{2+}$ observed**
**K-5-F_3Ile:**	Ac-YGGKAAAAKA**5-F_3Ile**AAKAAAAK-NH_2	899.4735	899.5036
**K-Ile:**	Ac-YGGKAAAAKA**Ile**AAKAAAAK-NH_2	872.5177	872.0341

(a)

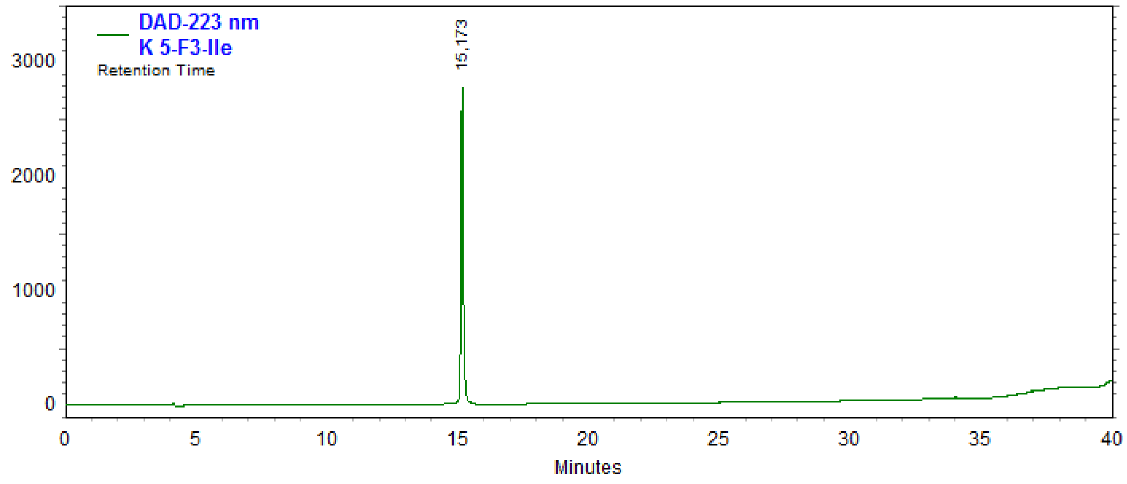

(b)

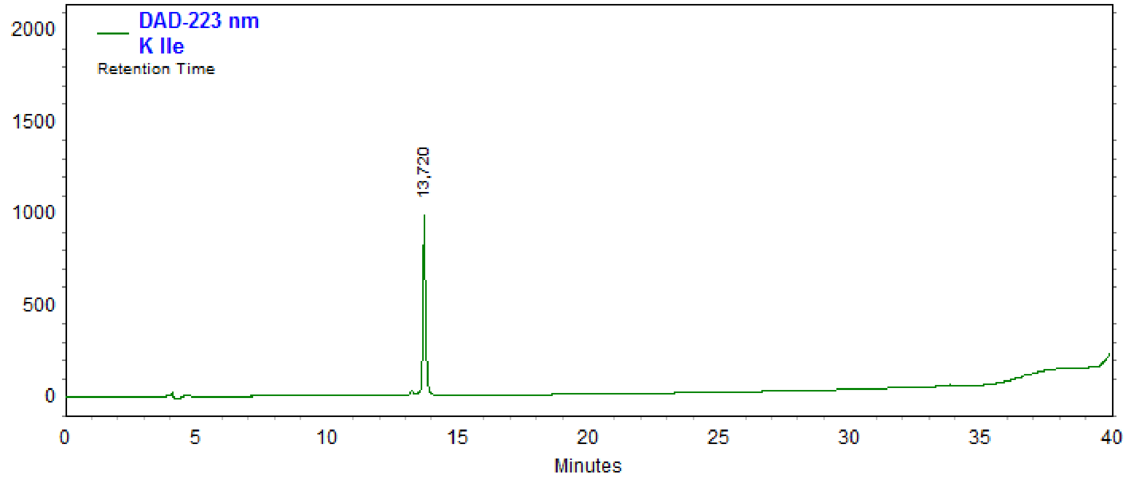

SI1: HPLC chromatograms of purified peptides

***(a) K-5-F$_3$Ile** and **(b) K-Ile**; column: Luna^{TM}C8 (5 um, 250 × 4.6 mm, Phenomenex®; Solvent A was 100% H_2O containing 0.1% TFA, solvent B was 100% acetonitrile containing 0.1% TFA. The flow rate was 1 mL/min; gradient from 5% B to 70% B in 30 min.*

Hydrophobicity studies:

N-Fmoc protected amino acids were dissolved in deionized water containing 40% CH_3CN and 0.1% TFA. Their retention times were determined on a C18 column (Capcell PAK C18, 5 µm). A linear gradient from 40 to 70% CH_3CN in 20 min was applied at room temperature. All experiments were performed in triplicate. The van der Waals volumes of the side chains were estimated starting from the β-carbon by summation of atomic increments and bond contributions (Zhao, Y. H.; Abraham, M. H.; Zissimos, A. M. *J. Org. Chem.* **2003**, *68*, 7368-7373.).

α-Helix propensity studies:

The lyophilized peptide was dissolved in 1 M NaCl, 1 mM sodium phosphate, 1 mM sodium citrate, and 1 mM sodium borate buffer (pH 7.0) and the concentration was determined via the tyrosine absorbance in 6 M guanidinium chloride (ε_{276} = 1.455 mol^{-1}cm^{-1}mL) using a Varian Cary 50 spectrophotometer (Varian Medical Systems, Palo Alto, CA, USA) and PMMA cuvettes (10 mm path length, 1.5 mL, Plastibrand®, VWR International GmbH, Darmstadt, Germany). CD measurements were performed at peptide concentrations of 30, 50, and 80 µM at pH 7.0 and 0 °C. CD spectra were recorded with a Jasco J-810 spectropolarimeter using Quartz cuvettes (1.0 mm path length). Each reported CD value represents the mean of at least 3 independent measurements. Data were collected from 250 to 200 nm at 0.2 nm intervals, 2 nm bandwidth, and 2 s response time. Spectra were background-corrected by subtraction of the corresponding buffer spectra. The measured CD data in mdeg were converted into molar ellipticity per residue [Θ]/ (10^3·mdeg·cm^2·dmol^{-1}·residue^{-1}). Thus, the mean residue molar ellipticity was independent of the peptide concentration. The fractional helical content of peptide (f_{helix}) was calculated from

the mean residue molar ellipticity at 222 nm and the number of backbone amides (N) 19 using the equation $f_{helix} = [\Theta]_{222}/ (40000(1-2.5/N))$. The helix propensity of the amino acid at the guest position was calculated from the f_{helix} of the corresponding peptide based on a modified Lifson-Roig theory (Chakrabartty, A.; Kortemme, T.; Baldwin, R. L. *Protein Sci.* **1994**, *3*, 843-852; Doig, A. J.; Chakrabartty, A.; Klingler, T. M.; Baldwin, R. L. *Biochemistry* **1994**, *33*, 3396-3403; Andersen N. H.; Tong, H. *Protein Sci.* **1997**, *6*, 1920-1936.).

Synthesis of enantiomerically pure alcohol 1

(*S*)-4-Benzyl-3-(4,4,4-trifluorobutanoyl)oxazolidin-2-one (I)[1]

A modified procedure by Wang and Resnick[2] was used: To a solution of 4,4,4-trifluorobutyric acid (15.0 g, 106 mmol. 1.00 equiv) and triethylamine (17.6 mL, 127 mmol, 1.20 equiv) in tetrahydrofuran (300 mL) at −78 °C was added trimethylacetyl chloride (13.6 mL, 111 mmol, 1.05 equiv). The resulting suspension was stirred for 5 min at −78 °C and 1 h at 0 °C. The solution was then recooled to −78 °C. In a separate flask a solution of (*S*)-4-benzyloxazolidin-2-one (22.4 g, 127 mmol, 1.20 equiv) in tetrahydrofuran (420 mL) was treated dropwise with *n*-butyllithium (2.5 M in hexane, 51.1 mL, 128 mol, 1.21 equiv) at −78 °C. The mixture was stirred for 1 h at −78 °C, before the thick slurry of the mixed anhydride was added via cannulation at −78 °C. The resulting solution was allowed to warm to rt overnight and stirred for an additional 2 d. The reaction was quenched by the addition of water. The aqueous layer was extracted with diethyl ether. The combined organic phases were washed with brine, dried over sodium sulfate, filtered, and concentrated in vacuo. The crude product was purified by flash column chromatography (SiO_2, ethyl acetate/hexane 1:4) to afford 25.7 g (81%, 85.2 mmol) of **I** as a light yellow oil which crystallized upon standing.

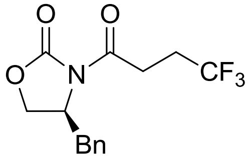

mp: 49–51 °C; ^{1}H NMR (500 MHz, $CDCl_3$): δ = 2.56 (m_c, 2 H, CH_2CF_3), 2.79 (dd, J = 13.4, 9.6 Hz, 1 H, $C\underline{H}_2Ph$), 3.16-3.31 (m, 3 H, $C\underline{H}_2Ph$, $COCH_2$), 4.19-4.25 (m, 2 H, OCH_2), 4.68 (m_c, 1 H, NCH), 7.19-7.36 (m, 5 H, Ph) ppm; ^{13}C NMR (126 MHz, $CDCl_3$): δ = 28.4 (q, J_{CF} = 27.6 Hz, $\underline{C}H_2CF_3$), 28.8 (q, J_{CF} = 2.7 Hz, $CO\underline{C}H_2$), 37.8 ($\underline{C}H_2Ph$), 55.2 (NCH), 66.5 (OCH_2), 127.5 (*p*-Ar-C), 128.4 (q, J_{CF} = 278 Hz, CF_3), 129.0 (*o*-Ar-C), 129.9 (*m*-Ar-C), 134.9 (*i*-Ar-C), 153.3 ($O\underline{C}ON$), 170.3 ($N\underline{C}OC$) ppm; ^{19}F NMR (376 MHz, $CDCl_3$): δ = -66.3 (t, J = 10.5 Hz, 3 F, CF_3) ppm.

(*S*)-4-Benzyl-3-[(*S*)-4,4,4-trifluoro-2-methylbutanoyl]oxazolidin-2-one (II)

A modified procedure by Wang and Resnick[2] was used: To a solution of sodium bis(trimethylsilyl)amide (1 M in tetrahydrofuran, 105 mL, 105 mmol. 1.10 equiv) in tetrahydrofuran (260 mL) at −78 °C was slowly added a precooled (−78 °C) solution of **I** (28.8 g, 95.7 mmol, 1.00 equiv) in tetrahydrofuran (260 mL). After the resulting yellow solution was stirred at −78 °C for 80 min, iodomethane (29.8 mL, 478 mmol, 5.00 equiv) in tetrahydrofuran (70 mL) was slowly added at that temperature. The reaction mixture was stirred at −78 °C for 4 h and an additional 10 min at rt. The reaction was quenched by the addition of aqueous

[1] Erdbrink, H.; Peuser, I.; Gerling, U. I. M.; Lentz, D.; Koksch, B.; Czekelius, C. *Org. Biomol. Chem.* **2012**, *10*, 8583-8586.
[2] Wang, Z.; Resnick, L. *Tetrahedron* **2008**, *64*, 6440-6443.

saturated ammonium chloride solution (150 mL) and water (30 mL). The aqueous layer was extracted with diethyl ether (3 × 200 mL). The combined organic phases were washed with brine, dried over sodium sulfate, filtered, and concentrated in vacuo. The crude product (dr > 95:5) was purified by flash column chromatography (SiO_2, ethyl acetate/hexane 1:4) to afford 23.3 g (77%, 73.7 mmol) of **II** as a light yellow oil.

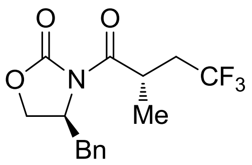

$[\alpha]_D^{22}$ = +77.6 (c = 1.00, $CHCl_3$); ^{1}H NMR (500 MHz, $CDCl_3$): δ = 1.34 (d, J = 7.0 Hz, 3 H, CH_3), 2.19 (m_c, 1 H, CH_2CF_3), 2.78-2.89* (m, 1 H, CH_2CF_3), 2.80* (dd, J = 13.4, 9.4 Hz, 1 H, $C\underline{H}_2Ph$), 3.23 (dd, J = 13.4, 3.4 Hz, 1 H, $C\underline{H}_2Ph$), 4.11 (m_c, 1 H, COCH), 4.18-4.26 (m, 2 H, OCH_2), 4.75 (m_c, 1 H, NCH), 7.20-7.35 (m, 5 H, Ph) ppm *signals overlap; ^{13}C NMR (126 MHz, $CDCl_3$): δ = 18.3 (CH_3), 32.3 (q, J_{CF} = 2.5 Hz, $CO\underline{C}H$), 36.3 (q, J_{CF} = 28.6 Hz, $\underline{C}H_2CF_3$), 37.7 ($\underline{C}H_2Ph$), 55.2 (NCH), 66.2 (OCH_2), 126.3 (q, J_{CF} = 277 Hz, CF_3), 127.4 (*p*-Ar-C), 128.9 (*o*-Ar-C), 129.4 (*m*-Ar-C), 134.9 (*i*-Ar-C), 152.8 ($O\underline{C}ON$), 174.6 ($N\underline{C}OC$) ppm; ^{19}F NMR (376 MHz, $CDCl_3$): δ = -64.7 (t, J = 10.9 Hz, 3 F, CF_3) ppm; IR (Film): ν = 704 (w), 748 (w), 977 (w), 1014 (m), 1177 (m), 1258 (m), 1358 (s), 1385 (s), 1698 (vs), 1789 (vs), 2982 (w), 3031 (w) cm^{-1}; HRMS (ESI): *m/z* $[M + Na]^+$ calcd. for $[C_{15}H_{16}F_3NNaO_3]^+$: 338.0974; found: 338.0984.

(S)-4,4,4-Trifluoro-2-methylbutan-1-ol (1)[2]

To a solution of **II** (23.3 g, 73.8 mmol, 1.00 equiv) in diethyl ether (246 mL) and methanol (6.1 mL) at 0 °C was slowly added lithium borohydride (2 M in tetrahydrofuran, 92.3 mL, 185 mmol, 2.50 equiv). The resulting solution was stirred at 0 °C for 3 h and then allowed to warm to rt overnight. The reaction mixture was quenched by slow addition of saturated aqueous Rochelle salt solution (90 mL). Stirring was continued at rt for an additional 2 h, until bubbling ceased. The aqueous layer was extracted with diethyl ether (3 × 100 mL). The combined organic phases were dried over sodium sulfate, filtered and concentrated on the rotary evaporator (40 °C, ~550 mbar). The resulting solution was filtered over a short plug of silica gel (diethyl ether/pentane 1:1) and concentrated on the rotary evaporator (40 °C, ~550 mbar) to afford 12.4 g of a solution of **1** in diethyl ether (24% diethyl ether by ^{1}H NMR, estimated yield: 9.40 g, 66.1 mmol, 89%) that was used directly in the next step.

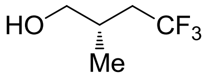

^{1}H NMR (400 MHz, $CDCl_3$): δ = 1.01 (d, J = 6.7 Hz, 3 H, CH_3), 1.80-1.90 (m, 1 H, CH_2CF_3), 1.93-2.20 (m, 1 H, $C\underline{H}CH_3$), 2.26-2.37 (m, 1 H, CH_2CF_3), 2.75 (br s, 1 H, OH), 3.36-3.44 (m, 1 H, $C\underline{H}_2OH$), 3.46-3.53 (m, 1 H, $C\underline{H}_2OH$) ppm; ^{19}F NMR (376 MHz, $CDCl_3$): δ = -63.6 (t, J = 11.4 Hz, 3 F, CF_3) ppm.

(*S*)-4,4,4-Trifluoro-2-methylbutyl-4-methylbenzenesulfonate (2).

To a stirred solution of the primary alcohol **1** (9.02 g, 63.5 mmol, 1.00 equiv) in pyridine (109 mL) at 0 °C was added *p*-toluenesulfonyl chloride (20.6 g, 108 mmol, 1.70 equiv) and DMAP (300 mg, 2.46 mmol). The mixture was stirred at 0 °C for 12 h and then allowed to warm to rt. The solution was treated with cold water (100 mL) followed by extraction with dichloromethane (4 × 130 mL). The combined organic phases were washed with brine, dried over sodium sulfate, filtered, and concentrated in vacuo. The crude product was purified by flash column chromatography (SiO_2, ethyl acetate/hexane 1:9) to afford 14.9 g (79%, 50.3 mmol) of **2** as a light yellow oil.

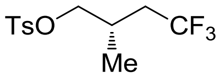

$[\alpha]_D^{22}$ = −2.4 (c = 2.00, $CHCl_3$); ^{1}H NMR (500 MHz, $CDCl_3$): δ = 1.02 (d, J = 6.7 Hz, 3 H, CH_3), 1.93 (m_c, 1 H, CH_2CF_3), 2.13-2.28 (m, 2 H, CH_2CF_3, $C\underline{H}CH_3$), 2.44 (s, 3 H, Ar-$C\underline{H}_3$), 3.86 (dd, J = 9.7, 5.8 Hz, 1 H, $C\underline{H}_2OTs$), 3.91 (dd, J = 9.7, 5.1 Hz, 1 H, $C\underline{H}_2OTs$), 7.35 (d, J = 8.3 Hz, 2 H, Ar), 7.77 (d, J = 8.3 Hz, 2 H, Ar) ppm; ^{13}C NMR (126 MHz, $CDCl_3$): δ = 16.4 (CH_3), 21.5 (Ar-$\underline{C}H_3$), 27.9 (q, J_{CF} = 2.3 Hz, $\underline{C}HCH_3$), 36.3 (q, J_{CF} = 28.3 Hz, $\underline{C}H_2CF_3$), 73.1 ($\underline{C}H_2OTs$), 126.5 (q, J_{CF} = 277 Hz, CF_3), 127.8 (*o*-Ar-C), 128.9 (*m*-Ar-C), 132.5 (*p*-Ar-C), 145.0 (*i*-Ar-C) ppm; ^{19}F NMR (376 MHz, $CDCl_3$): δ = -63.6 (t, J = 10.9 Hz, 3 F, CF_3) ppm; IR (Film): ν = 666 (w), 814 (w), 939 (w), 997 (m), 1177 (m), 1256 (m), 1362 (m), 1598 (w), 2978 (w) cm^{-1}; HRMS (ESI): *m/z* $[M + Na]^+$ calcd. for $[C_{12}H_{15}F_3NaO_3S]^+$: 319.0586; found: 319.0584.

(*R*)-5,5,5-Trifluoro-3-methylpentanenitrile (3).

A modified procedure by Molinski[3] was used: A mixture of **2** (13.5 g, 45.6 mmol, 1.00 equiv), NaCN (11.2 g, 229 mmol, 5.00 equiv), and NaI (682 mg, 4.55 mmol, 10 mol %) in dimethylsulfoxide (113 mL) was heated to 60 °C for 2.5 h. The reaction mixture was cooled to ambient temperature and water (110 mL) was added. The aqueous phase was extracted with diethylether (4 × 200 mL). The combined organic phases were washed with brine (2 × 150 mL), dried over sodium sulfate, filtered, and concentrated on the rotary evaporator (40 °C, ~700 mbar). The excess Et_2O was removed by distillation at 100 °C to afford 5.93 g (85%, 39.2 mmol) of the nitrile **3** as a yellow to orange oil, which was used directly in the next step.

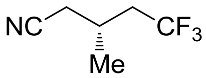

$[\alpha]_D^{22}$ = +8.6 (c = 2.24, $CHCl_3$); ^{1}H NMR (400 MHz, $CDCl_3$): δ = 1.12 (d, J = 6.3 Hz, 3 H, CH_3), 2.00-2.24 (m, 3 H, CH_2CF_3, $C\underline{H}CH_3$), 2.36 (m_c, 2 H, $NCCH_2$) ppm; ^{13}C NMR (101 MHz, $CDCl_3$): δ = 19.1 (CH_3), 23.7 (q, J_{CF} = 1.2 Hz, $NC\underline{C}H_2$), 25.0 (q, J_{CF} = 2.6 Hz, $\underline{C}HCH_3$), 38.4 (q, J_{CF} =

[3] Pigza, J. A.; Quach, T.; Molinski, T. F. *J. Org. Chem.* **2009**, *74*, 5510-5515.

28.2 Hz, $\underline{C}H_2CF_3$), 117.3 (N$\underline{C}$CH$_2$), 126.1 (q, J_{CF} = 277 Hz, CF_3) ppm; ^{19}F NMR (376 MHz, $CDCl_3$): δ = -63.9 (t, J = 10.5 Hz, 3 F, CF_3) ppm; IR (Film): ν = 685 (w), 744 (w), 914 (w), 1041 (m), 1140 (s), 1171 (s), 1257 (s), 1387 (m), 2249 (w), 2945 (w), 2976 (w) cm^{-1}. HRMS (ESI) or EI could not be generated.

(*R*)-5,5,5-Trifluoro-3-methylpentanoic acid (4).

A modified procedure by Molinski[3] was used: A solution of **3** (5.93 g, 39.2 mmol) in concentrated HCl (120 mL) was heated to reflux for 2.5 h. The reaction mixture was cooled to ambient temperature and water (100 mL) was added. The aqueous phase was extracted with dichloromethane (4 × 200 mL). The combined organic phases were dried over sodium sulfate, filtered and concentrated under reduced pressure to afford 5.39 g (80%, 31.7 mmol) of the acid **4** as a brownish oil, which was used directly in the next step.

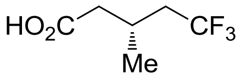

$[\alpha]_D^{22}$ = +4.0 (c = 1.00, $CHCl_3$); ^{1}H NMR (500 MHz, $CDCl_3$): δ = 1.10 (d, J = 6.5 Hz, 3 H, CH_3), 2.04 (m$_c$, 1 H, CH_2CF_3), 2.17-2.28 (m, 1 H, CH_2CF_3), 2.31* (dd, J = 15.1, 7.2 Hz, 1 H, $COCH_2$), 2.33-2.40* (m, 1 H, C$\underline{H}$CH$_3$), 2.46 (dd, J = 15.1, 5.4 Hz, 1 H, $COCH_2$), 12.1 (br s, 1 H, COOH) ppm *signal overlap; ^{13}C NMR (126 MHz, $CDCl_3$): δ = 19.7 (CH_3), 24.8 (q, J_{CF} = 2.3 Hz, $\underline{C}HCH_3$), 39.2 (q, J_{CF} = 27.6 Hz, $\underline{C}H_2CF_3$), 40.5 (CO$\underline{C}$H$_2$), 126.8 (q, J_{CF} = 277 Hz, CF_3), 178.6 (COOH) ppm; ^{19}F NMR (376 MHz, $CDCl_3$): δ = -63.6 (t, J = 11.2 Hz, 3 F, CF_3) ppm; IR (Film): ν = 665 (w), 913 (w), 1040 (s), 1169 (s), 1256 (s), 1417 (m), 1441 (m), 1714 (vs), 2683 (w), 2945 (m), 2975 (m) cm^{-1}; HRMS (ESI): *m/z* $[M-H]^-$ calcd. for $[C_6H_8F_3O_2]^-$: 169.0482; found: 169.0480.

(*S*)-4-Benzyl-3-[(*R*)-5,5,5-trifluoro-3-methylpentanoyl]oxazolidin-2-one (5).

To a solution of acid **4** (3.50 g, 20.6 mmol, 1.00 equiv) and triethylamine (3.42 mL, 24.7 mmol, 1.20 equiv) in tetrahydrofuran (95 mL) at −78 °C was added trimethylacetyl chloride (2.66 mL, 21.6 mmol, 1.05 equiv). The resulting suspension was stirred for 5 min at −78 °C and 90 min at 0 °C. The solution was then recooled to −78 °C. In a separate flask a solution of (*S*)-4-benzyloxazolidin-2-one (4.56 g, 25.7 mmol, 1.20 equiv) in tetrahydrofuran (83 mL) was treated dropwise with *n*-butyllithium (2.5 M in hexane, 10.4 mL, 25.9 mol, 1.21 equiv) at −78 °C. The mixture was stirred for 1 h at −78 °C before the thick slurry of the mixed anhydride was added *via* cannulation at −78 °C. The resulting solution was allowed to warm to rt overnight and stirred for an additional 5 h at rt. The reaction was quenched by the addition of water (100 mL). The aqueous layer was extracted with diethyl ether (4 × 120 mL). The combined organic phases were washed with brine, dried over sodium sulfate, filtered, and concentrated in vacuo. The crude product was purified by flash column chromatography (SiO_2, ethyl acetate/hexane 1:4) to afford 5.76 g (84%, 17.5 mmol) of **5** as a light yellow oil.

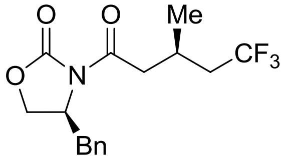

$[\alpha]_D^{22}$ = +48.2 (c = 1.00, $CHCl_3$); ^{1}H NMR (500 MHz, $CDCl_3$): δ = 1.13 (d, *J* = 6.8 Hz, 3 H, CH_3), 2.01-2.13 (m, 1 H, CH_2CF_3), 2.24-2.35 (m, 1 H, CH_2CF_3), 2.51 (m_c, 1 H, C<u>H</u>CH_3), 2.77 (dd, *J* = 13.4, 9.6 Hz, 1 H, C<u>H</u>$_2$Ph), 2.91 (dd, *J* = 17.3, 7.1 Hz, 1 H, COC<u>H</u>$_2$), 3.02 (dd, *J* = 17.3, 6.2 Hz, 1 H, COC<u>H</u>$_2$), 3.28 (dd, *J* = 13.4, 3.4 Hz, 1 H, C<u>H</u>$_2$Ph), 4.15-4.21 (m, 2 H, OCH_2), 4.68 (m_c, 1 H, NCH), 7.20-7.35 (m, 5 H, Ph) ppm; ^{13}C NMR (126 MHz, $CDCl_3$): δ = 19.9 (CH_3), 24.2 (q, J_{CF} = 2.4 Hz, <u>C</u>HCH_3), 37.8 (<u>C</u>H$_2$Ph), 39.3 (q, J_{CF} = 27.4 Hz, <u>C</u>H$_2$CF$_3$), 41.7 (CO<u>C</u>H$_2$), 55.0 (NCH), 66.2 (OCH_2), 126.8 (q, J_{CF} = 277 Hz, CF_3), 127.3 (*p*-Ar-C), 128.8 (*o*-Ar-C), 129.3 (*m*-Ar-C), 135.1 (*i*-Ar-C), 153.3 (O<u>C</u>ON), 171.2 (N<u>C</u>OC) ppm; ^{19}F NMR (376 MHz, $CDCl_3$): δ = -63.2 (t, *J* = 11.3 Hz, 3 F, CF_3) ppm; IR (Film): ν = 701 (m), 749 (w), 763 (w), 1039 (m), 1212 (s), 1254 (m), 1387 (s), 1455 (m), 1699 (s), 1782 (vs), 2941 (w), 2973 (w) cm^{-1}; HRMS (ESI): *m/z* $[M + Na]^+$ calcd. for $[C_{16}H_{18}F_3NNaO_3]^+$: 352.1131; found: 352.1124.

(*S*)-3-[(2*S*,3*S*)-2-Azido-5,5,5-trifluoro-3-methylpentanoyl]-4-benzyloxazolidin-2-one (5') and (*S*)-3-[(2*R*,3*S*)-2-Azido-5,5,5-trifluoro-3-methylpentanoyl]-4-benzyloxazolidin-2-one (9).

To a stirred solution of tetrahydrofuran (14.0 mL) at −78 °C was added potassium bis(trimethylsilyl)amide (0.5 M in toluene, 9.83 mL, 4.92 mmol, 1.10 equiv). To the resulting solution was slowly added *via* cannula a precooled (−78 °C) solution of **5** (1.47 g, 4.46 mmol, 1.00 equiv) in tetrahydrofuran (13.0 mL). The reaction mixture was stirred at −78 °C for 30 min. To the above solution of the potassium enolate, stirred at −78 °C, was then added via cannula a precooled (−78 °C) solution of trisyl azide (0.4 M in tetrahydrofuran, 13.7 mL, 5.49 mmol, 1.23 equiv). After a reaction time of 5.5 min glacial acetic acid (1.15 mL, 20.1 mmol, 4.60 equiv) was added and the cooling bath was removed. The reaction was stirred at rt for 13 h. Brine was added followed by extraction with dichloromethane (4 × 25 mL). The combined organic phases were washed with saturated sodium bicarbonate solution, dried over sodium sulfate, filtered, and concentrated in vacuo. The crude product (dr = 92:8) was purified by flash column chromatography (SiO_2, ethyl acetate/hexane 1:4) to afford 1.17 g (70%, 3.16 mmol) of **5'** as a colorless oil and 83.0 mg (5.0%, 0.224 mmol) of **9** as a colorless oil.

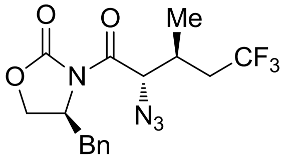

$[\alpha]_D^{22}$ = +46.1 (c = 2.00, $CHCl_3$), ^{1}H NMR (500 MHz, $CDCl_3$): δ = 1.20 (d, *J* = 6.8 Hz, 3 H, CH_3), 1.98-2.09 (m, 1 H, CH_2CF_3), 2.39-2.50 (m, 2 H, CH_2CF_3, C<u>H</u>CH_3), 2.88 (dd, *J* = 13.5, 9.2 Hz, 1 H, C<u>H</u>$_2$Ph), 3.31 (dd, *J* = 13.5, 3.3 Hz, 1 H, C<u>H</u>$_2$Ph), 4.25-4.26 (m, 2 H, OCH_2), 4.71 (m_c, 1 H, NCH), 5.16 (d, *J* = 5.5 Hz, CHN_3), 7.21-7.37 (m, 5 H, Ph) ppm; ^{13}C NMR (126 MHz, $CDCl_3$): δ =

17.2 (CH_3), 30.3 (q, J_{CF} = 2.4 Hz, $\underline{C}HCH_3$), 34.9 (q, J_{CF} = 28.2 Hz, $\underline{C}H_2CF_3$), 37.4 ($\underline{C}H_2Ph$), 55.4 (NCH), 64.8 (CHN_3), 66.7 (OCH_2), 126.9 (q, J_{CF} = 277 Hz, CF_3), 127.5 (*p*-Ar-C), 129.0 (*o*-Ar-C), 129.3 (*m*-Ar-C), 134.5 (*i*-Ar-C), 152.9 ($O\underline{C}ON$), 168.9 ($N\underline{C}OC$) ppm; ^{19}F NMR (376 MHz, $CDCl_3$): δ = -63.8 (t, *J* = 11.1 Hz, 3 F, CF_3) ppm; IR (Film): ν = 703 (w), 749 (w), 913 (w), 1038 (m), 1212 (s), 1254 (s), 1390 (s), 1704 (s), 1781 (vs), 2111 (s), 2979 (w) cm^{-1}; HRMS (ESI): *m/z* $[M + Na]^+$ calcd. for $[C_{16}H_{17}F_3N_4NaO_3]^+$: 393.1145; found: 393.1154.

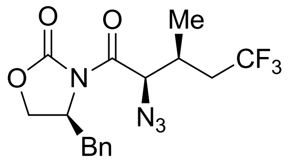

$[\alpha]_D^{22}$ = +77.8 (c = 1.00, $CHCl_3$); ^{1}H NMR (500 MHz, $CDCl_3$): δ = 1.10 (d, *J* = 6.9 Hz, 3 H, CH_3), 2.14-2.25 (m, 1 H, CH_2CF_3), 2.32-2.43 (m, 1 H, CH_2CF_3), 2.60 (m_c, 1 H, $C\underline{H}CH_3$), 2.74 (dd, *J* = 13.3, 9.9 Hz, 1 H, $C\underline{H}_2Ph$), 3.35 (dd, *J* = 13.3, 3.6 Hz, 1 H, $C\underline{H}_2Ph$), 4.22-4.31 (m, 2 H, OCH_2), 4.79 (m_c, 1 H, NCH), 5.18 (d, *J* = 4.0 Hz, CHN_3), 7.22-7.36 (m, 5 H, Ph) ppm; ^{13}C NMR (126 MHz, $CDCl_3$): δ = 14.4 (m_c, CH_3), 30.4 (q, J_{CF} = 2.4 Hz, $\underline{C}HCH_3$), 37.4 (q, J_{CF} = 28.3 Hz, $\underline{C}H_2CF_3$), 38.0 ($\underline{C}H_2Ph$), 55.0 (NCH), 64.7 (CHN_3), 67.0 (OCH_2), 126.5 (q, J_{CF} = 277 Hz, CF_3), 127.5 (*p*-Ar-C), 129.0 (*o*-Ar-C), 129.2 (*m*-Ar-C), 134.5 (*i*-Ar-C), 152.9 ($O\underline{C}ON$), 168.7 ($N\underline{C}OC$) ppm; ^{19}F NMR (376 MHz, $CDCl_3$): δ = -63.3 (t, *J* = 10.8 Hz, 3 F, CF_3) ppm; IR (Film): ν = 703 (m), 763 (m), 912 (m), 1044 (s), 1172 (s), 1255 (s), 1392 (s), 1705 (s), 1780 (vs), 2112 (s), 2945 (w), 2981 (w) cm^{-1}; HRMS (ESI): *m/z* $[M + Na]^+$ calcd. for $[C_{16}H_{17}F_3N_4NaO_3]^+$: 393.1145; found: 393.1182.

(*S*)-3-{(2*S*,3*S*)-2-[*tert*-Butoxycarbonyl]amino-5,5,5-trifluoro-3-methylpentanoyl}-4-benzyloxazolidin-2-one (6).

Palladium on charcoal (10% Pd, 313 mg, 0.295 mmol) was suspended in ethyl acetate (20.0 mL) and saturated with hydrogen gas (1 bar) for 20 minutes. Afterwards, di-*tert*-butyl dicarbonate (2.26 g, 10.4 mmol, 3.53 equiv) in ethyl acetate (2.0 mL) and a solution of **5'** (1.09 g, 2.95 mmol, 1.00 equiv) in ethyl acetate (3.0 mL) were added at rt. The reaction mixture was stirred at rt for 2.5 h under 1 bar (balloon) of hydrogen gas. The reaction mixture was filtered over celite and concentrated to dryness. The crude material was purified by flash column chromatography (SiO_2, ethyl acetetate/hexane 1:5) to afford 1.02 g (78%, 2.30 mmol) of the title compound **6** as colorless solid.

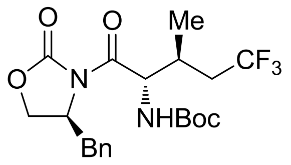

mp 115–117 °C; $[\alpha]_D^{22}$ = +64.6 (c = 1.00, $CHCl_3$), ^{1}H NMR (500 MHz, $CDCl_3$): δ = 1.20 (d, *J* = 6.8 Hz, 3 H, CH_3), 1.46 (s, 9 H, $CO_2C(C\underline{H}_3)_3$), 1.87-1.98 (m, 1 H, CH_2CF_3), 2.18-2.34 (m,

2 H, CH_2CF_3, C<u>H</u>CH_3), 2.78 (dd, *J* = 13.5, 9.8 Hz, 1 H, C<u>H</u>$_2$Ph), 3.33 (dd, *J* = 13.5, 2.9 Hz, 1 H, C<u>H</u>$_2$Ph), 4.20-4.24 (m, 2 H, OCH_2), 4.61-4.66 (m, 1 H, NCH), 5.23 (br d, *J* = 9.2 Hz, 1 H, NH), 5.55 (dd, *J* = 9.2, 4.2 Hz, 1 H, C<u>H</u>NHBoc), 7.20-7.36 (m, 5 H, Ph) ppm; ^{13}C NMR (126 MHz, $CDCl_3$): δ = 18.0 (CH_3), 28.2 (CO_2C(<u>C</u>H_3)$_3$), 31.2 (m, <u>C</u>HCH_3), 34.7 (q, J_{CF} = 28.3 Hz, <u>C</u>H_2CF_3), 37.5 (<u>C</u>H_2Ph), 55.6 (NCH), 56.7 (<u>C</u>HNHBoc), 66.5 (OCH_2), 80.3 (CO_2<u>C</u>(CH_3)$_3$), 127.0 (q, J_{CF} = 277 Hz, CF_3), 127.5 (*p*-Ar-C), 129.0 (*o*-Ar-C), 129.4 (*m*-Ar-C), 134.9 (*i*-Ar-C), 152.7 (O<u>C</u>ON), 155.6 (<u>C</u>$O_2C(CH_3)_3$), 172.2 (N<u>C</u>OC) ppm; ^{19}F NMR (376 MHz, $CDCl_3$): δ = -63.9 (t, *J* = 11.0 Hz, 3 F, CF_3) ppm; IR (Film): ν = 702 (w), 761 (m), 1044 (m), 1169 (s), 1212 (m), 1389 (s), 1498 (m), 1698 (s), 1785 (vs), 2979 (w), 3376 (w) cm^{-1}; HRMS (ESI): *m/z* [M + Na]$^+$ calcd. for $[C_{21}H_{27}F_3N_2NaO_5]^+$: 467.1764; found: 467.1784.

(2*S*,3*S*)-2-[(*tert*-Butoxycarbonyl)amino]-5,5,5-trifluoro-3-methylpentanoic acid (7).

To a stirred solution of **6** (1.00 g, 2.25 mmol) in tetrahydrofuran/water 3:1 (22.5 mL) at 0 °C was added a precooled (0 °C) solution of LiOH (283 mg, 6.75 mol, 3.00 equiv) and H_2O_2 (30%, 0.417 mL, 13.5 mmol, 6.00 equiv) in tetrahydrofuran/water 3:1 (7.0 mL). After stirring at 0 °C for 2.5 h, the reaction was slowly treated with aqueous Na_2SO_3 (1 M, 6.60 equiv) and allowed to warm to rt. After removal of the tetrahydrofuran on the rotary evaporator, the residue was washed with dichloromethane (3 × 10 mL). The aqueous phase was carefully acidified with HCl (2 N) to pH ~3 and extracted with ethyl acetate (4 × 12 mL). The combined organic phases were dried over sodium sulfate, filtered, and concentrated in vacuo to afford 594 mg of pure **7** (92%, 2.08 mmol) as a colorless solid.

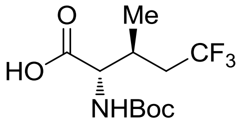

mp 105–108 °C; $[\alpha]_D^{22}$ = +28.7 (c = 1.00, $CHCl_3$); ^{1}H NMR (500 MHz, C_3D_6O): δ = 1.11 (d, *J* = 6.8 Hz, 3 H, CH_3), 1.40 (s, 9 H, CO_2C(C<u>H</u>$_3$)$_3$), 2.16-2.28 (m, 1 H, CH_2CF_3), 2.34-2.45 (m, 2 H, CH_2CF_3, C<u>H</u>CH_3), 4.30 (d, *J* = 4.5 Hz, 1 H, C<u>H</u>NHBoc), 6.29 (br s, 1 H, NH), 11.34 (br s, 1 H, COOH) ppm; ^{13}C NMR (126 MHz, C_3D_6O): δ = 16.6 (CH_3), 28.5 (CO_2C(<u>C</u>H_3)$_3$), 31.4 (q, J_{CF} = 2.3 Hz, <u>C</u>HCH_3), 36.7 (q, J_{CF} = 27.6 Hz, <u>C</u>H_2CF_3), 58.4 (<u>C</u>HNHBoc), 79.6 (CO_2<u>C</u>(CH_3)$_3$), 128.3 (q, J_{CF} = 276 Hz, CF_3), 156.6 (<u>C</u>$O_2C(CH_3)_3$), 172.7 (COOH) ppm; ^{19}F NMR (376 MHz, C_3D_6O): δ = -64.1 (t, *J* = 11.3 Hz, 3 F, CF_3) ppm; IR (Film): ν = 650 (w), 682 (w), 778 (w), 867 (w), 1045 (m), 1171 (s), 1256 (s), 1396 (m), 1509 (m), 1715 (vs), 2555 (w), 2981 (m), 3317 (w) cm^{-1}; HRMS (ESI): *m/z* [M − H]$^-$ calcd. for $[C_{11}H_{17}F_3NO_4]^-$: 284.1115; found: 284.1216.

(*S*)-4-Benzyl-3-[(2*S*,3*S*)-2-bromo-5,5,5-trifluoro-3-methylpentanoyl]oxazolidin-2-one (8a) and (*S*)-4-Benzyl-3-[(2*R*,3*S*)-2-bromo-5,5,5-trifluoro-3-methylpentanoyl]oxazolidin-2-one (8b).

To a stirred solution of **5** (350 mg, 1.06 mmol, 1.00 equiv) in dichloromethane (10.0 mL) at −78 °C was slowly added freshly destilled *N*,*N*-diisopropylethylamine (218 µL, 1.28 mmol, 1.20

equiv), followed by dropwise addition of dibutylboryl trifluoromethanesulfonate (1 M in dichloromethane, 1.12 mL, 1.12 mmol, 1.06 equiv). The resulting solution was stirred at −78 °C for 15 min and then at 0 °C for 70 min. The pale yellow solution was cooled to −78 °C and added via cannula to a precooled (−78 °C) suspension of *N*-bromosuccinimide (209 mg, 1.17 mmol, 1.11 equiv) in dichloromethane (3.0 mL). The reaction mixture was stirred at −78 °C for 2 h and was then allowed to warm to rt. The solution was quenched by pouring into an aqueous sodium bisulfate-brine-solution (0.5 N). The aqueous phase was extracted with ethyl acetate (3 × 10 mL). The combined organic phases were washed with aqueous sodium thiosulfate-brine-solution (0.5 N), and with brine, dried over sodium sulfate, filtered, and concentrated in vacuo. The crude product (dr = 91:9) was purified by flash column chromatography (SiO_2, ethyl acetate/hexane 1:5.5) to afford 321 mg (74%, 0.786 mmol) of **8a** as light yellow oil and 16.8 mg (3.8%, 0.041 mmol) of **8b** as light yellow oil.

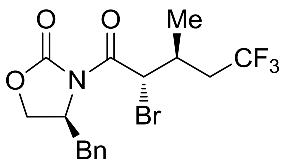

$[\alpha]_D^{22}$ = +39.0 (c = 2.00, $CHCl_3$); ^{1}H NMR (500 MHz, $CDCl_3$): δ = 1.22 (d, *J* = 6.6 Hz, 3 H, CH_3), 2.07 (m_c, 1 H, CH_2CF_3), 2.57 (m_c, 1 H, C<u>H</u>CH_3), 2.76-2.87* (m, 1 H, CH_2CF_3), 2.81* (dd, *J* = 13.5, 3.4 Hz, 1 H, C<u>H</u>$_2$Ph), 3.31 (dd, *J* = 13.5, 3.4 Hz, 1 H, C<u>H</u>$_2$Ph), 4.21-4.27 (m, 2 H, OCH_2), 4.73 (m_c, 1 H, NCH), 5.62 (d, *J* = 7.2 Hz, CHBr), 7.22-7.36 (m, 5 H, Ph) ppm *signals overlap; ^{13}C NMR (126 MHz, $CDCl_3$): δ = 18.1 (CH_3), 31.1 (m, <u>C</u>HCH_3), 36.9 (<u>C</u>H$_2$Ph), 37.3 (q, J_{CF} = 28.1 Hz, <u>C</u>H$_2CF_3$), 49.7 (CHBr), 55.2 (NCH), 66.3 (OCH_2), 126.9 (q, J_{CF} = 277 Hz, CF_3), 127.5 (*p*-Ar-C), 129.0 (*o*-Ar-C), 129.4 (*m*-Ar-C), 134.6 (*i*-Ar-C), 152.5 (O<u>C</u>ON), 168.0 (N<u>C</u>OC) ppm; ^{19}F NMR (376 MHz, $CDCl_3$): δ = -63.2 (t, *J* = 11.5 Hz, 3 F, CF_3) ppm; IR (Film): ν = 703 (w), 760 (w), 1033 (m), 1138 (m), 1214 (m), 1256 (m), 1390 (s), 1704 (s), 1782 (vs), 2925 (w), 2980 (w), 3030 (w) cm^{-1}; HRMS (ESI): *m/z* $[M + Na]^+$ calcd. for $[C_{16}H_{17}BrF_3NNaO_3]^+$: 430.0236; found: 430.0226.

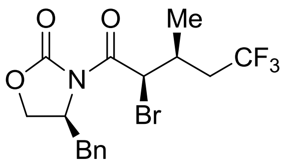

$[\alpha]_D^{22}$ = +33.1 (c = 0.92, $CHCl_3$); ^{1}H NMR (400 MHz, $CDCl_3$): δ = 1.26 (d, *J* = 6.7 Hz, 3 H, CH_3), 2.22-2.36 (m, 1 H, CH_2CF_3), 2.42-2.60 (m, 2 H, CH_2CF_3, C<u>H</u>CH_3), 2.78 (dd, *J* = 13.3, 9.8 Hz, 1 H, C<u>H</u>$_2$Ph), 3.32 (dd, *J* = 13.3, 3.5 Hz, 1 H, C<u>H</u>$_2$Ph), 4.21-4.30 (m, 2 H, OCH_2), 4.71 (m_c, 1 H, NCH), 5.74 (d, *J* = 5.2 Hz, CHBr), 7.20-7.36 (m, 5 H, Ph) ppm; ^{13}C NMR (101 MHz, $CDCl_3$): δ = 16.8 (CH_3), 31.5 (q, J_{CF} = 2.4 Hz <u>C</u>HCH_3), 37.9* (q, J_{CF} = 27.8 Hz, <u>C</u>H$_2CF_3$), 38.1* (<u>C</u>H$_2$Ph), 49.9 (CHBr), 55.8 (NCH), 66.6 (OCH_2), 127.6 (*p*-Ar-C), 127.9 (m, CF_3), 129.1 (*o*-Ar-C), 129.3 (*m*-Ar-C), 134.7 (*i*-Ar-C), 152.6 (O<u>C</u>ON), 167.8 (N<u>C</u>OC) ppm *signal overlap; ^{19}F NMR (376 MHz,

$CDCl_3$): δ = -63.3 (t, J = 11.0 Hz, 3 F, CF_3) ppm; IR (Film): ν = 702 (w), 760 (w), 1034 (w), 1195 (m), 1256 (s), 1382 (s), 1704 (s), 1782 (vs), 2853 (w), 2924 (w) cm^{-1}; HRMS (ESI): *m/z* [M + Na]$^+$ calcd. for $[C_{16}H_{17}BrF_3NNaO_3]^+$: 430.0236; found: 430.0266.

(*S*)-3-[(2*R*,3*S*)-2-Azido-5,5,5-trifluoro-3-methylpentanoyl]-4-benzyloxazolidin-2-one (9) (*S*)-3-[(2*S*,3*S*)-2-Azido-5,5,5-trifluoro-3-methylpentanoyl]-4-benzyloxazolidin-2-one (5') from (8a).

To a stirred solution **8a** (293 mg, 0.718 mmol, 1.00 equiv) in dichloromethane (7.0 mL) at 0 °C was added tetramethylguanidinium azide (569 mg, 3.60 mmol, 5.01 equiv) in one portion. The resulting solution was stirred at 0 °C for 19 h and was quenched by addition of saturated aqueous sodium hydrogensulfate (4 mL). The aqueous phase was extracted with dichloromethane (3 × 4 mL). The combined organic phases were washed with brine, dried over sodium sulfate, filtered, and concentrated in vacuo. The crude product (dr = 97:3) was purified by preparative TLC (SiO_2, dichloromethane/toluene 2:1) to afford 212 mg (79%, 0.572 mmol) of **9** and 3.0 mg (1.1%, 8.10 µmol) of **5'**.

(*S*)-3-{(2*R*,3*S*)-2-[*tert*-Butoxycarbonyl]amino-5,5,5-trifluoro-3-methylpentanoyl}-4-benzyloxazolidin-2-one (9').

Palladium on charcoal (10% Pd, 54.0 mg, 51.0 µmol) was suspended in ethyl acetate (3.0 mL) and saturated with hydrogen gas (1 bar) for 30 minutes. Afterwards, di-*tert*-butyl dicarbonate (335 mg, 1.53 mmol, 3.00 equiv) in ethyl acetate (1.0 mL) and a solution of **9** (191 mg, 0.516 mmol, 1.00 equiv) in ethyl acetate (1.0 mL) were added at rt. The reaction mixture was stirred at rt for 2.5 h under 1 bar (balloon) of hydrogen gas. The reaction mixture was filtered over celite and concentrated to dryness. The crude material was purified by flash column chromatography (SiO_2, ethyl acetetate/hexane 1:4) to afford 186 mg (81%, 0.419 mmol) of the title compound **9'** as colorless oil.

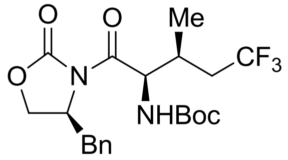

$[\alpha]_D^{22}$ = +5.9 (c = 0.89, $CHCl_3$); ^{1}H NMR (500 MHz, $CDCl_3$): δ = 0.96 (d, J = 6.8 Hz, 3 H, CH_3), 1.44 (s, 9 H, $CO_2C(C\underline{H}_3)_3$), 2.05-2.14 (m, 1 H, CH_2CF_3), 2.44-2.58 (m, 2 H, CH_2CF_3, $C\underline{H}CH_3$), 2.76 (dd, J = 13.4, 9.6 Hz, 1 H, $C\underline{H}_2Ph$), 3.25 (dd, J = 13.4, 3.6 Hz, 1 H, $C\underline{H}_2Ph$), 4.20-4.28 (m, 2 H, OCH_2), 4.70-4.75 (m, 1 H, NCH), 5.29 (br d, J = 9.7 Hz, 1 H, NH), 5.42 (dd, J = 9.7, ~1.6 Hz, 1 H, C$\underline{H}$NHBoc), 7.18-7.20 (m, 2 H, Ph), 7.25-7.34 (m, 3 H, Ph) ppm; ^{13}C NMR (126 MHz, $CDCl_3$): δ = 12.6 (CH_3), 28.2 ($CO_2C(\underline{C}H_3)_3$), 30.2 (m, $\underline{C}HCH_3$), 37.9* (q, J_{CF} = 28.0 Hz, $\underline{C}H_2CF_3$), 38.1* ($\underline{C}H_2Ph$), 54.8 (NCH), 56.5 ($\underline{C}$HNHBoc), 66.8 (OCH_2), 80.3 ($CO_2\underline{C}(CH_3)_3$), 126.7 (q, J_{CF} = 277 Hz, CF_3), 127.5 (*p*-Ar-C), 129.0 (*o*-Ar-C), 129.2 (*m*-Ar-C), 134.6 (*i*-Ar-C), 152.7 (O$\underline{C}$ON), 155.7 ($\underline{C}O_2C(CH_3)_3$), 171.3 (N$\underline{C}$OC) ppm *signal overlap; ^{19}F NMR (376 MHz, $CDCl_3$):

δ = -63.0 (t, J = 10.5 Hz, 3 F, CF_3) ppm; IR (Film): ν = 702 (w), 762 (m), 1048 (m), 1169 (m), 1255 (m), 1368 (s), 1498 (m), 1697 (s), 1784 (vs), 2933 (w), 2979 (w) cm^{-1}; HRMS (ESI): *m/z* $[M + Na]^+$ calcd. for $[C_{21}H_{27}F_3N_2NaO_5]^+$: 467.1764; found: 467.1793.

(2*R*,3*S*)-2-[(*tert*-Butoxycarbonyl)amino]-5,5,5-trifluoro-3-methylpentanoic acid (10).

To a stirred solution of **9'** (182 mg, 0.409 mmol) in tetrahydrofuran/water 3:1 (4.0 mL) at 0 °C was added a precooled (0 °C) solution of LiOH (51.5 mg, 1.23 mol, 3.01 equiv) and H_2O_2 (30%, 75.2 µL, 2.45 mmol, 6.00 equiv) in tetrahydrofuran/water 3:1 (2.0 mL). After stirring at 0 °C for 1.5 h, the reaction was slowly treated with aqueous Na_2SO_3 (1 M, 6.60 equiv) and allowed to warm to rt. After removal of the tetrahydrofuran on the rotary evaporator, the residue was washed with dichloromethane (3 × ~1.5 mL). The aqueous phase was carefully acidified with HCl (2 N) to pH ~3 and extracted with ethyl acetate (3 × 2 mL). The combined organic phases were dried over sodium sulfate, filtered and concentrated in vacuo to afford 101 mg (86%, 0.354 mmol) of **10** as a colorless oil.

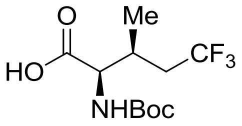

$[\alpha]_D^{22}$ = −27.5 (c = 0.82, $CHCl_3$); ^{1}H NMR (500 MHz, C_3D_6O): δ = 1.06 (d, J = 6.8 Hz, 3 H, CH_3), 1.41 (s, 9 H, $CO_2C(C\underline{H}_3)_3$), 2.07-2.18 (m, 1 H, CH_2CF_3), 2.44-2.58 (m, 2 H, CH_2CF_3, $C\underline{H}CH_3$), 4.41 (dd, J = 9.1, 3.6 Hz, 1 H, $C\underline{H}$NHBoc), 6.18 (br s, 1 H, NH) ppm signal for COOH could not be detected; ^{13}C NMR (126 MHz, C_3D_6O): δ = 15.1 (CH_3), 28.5 ($CO_2C(\underline{C}H_3)_3$), 31.1 ($m_c$, $\underline{C}HCH_3$), 37.4 (q, J_{CF} = 27.6 Hz, $\underline{C}H_2CF_3$), 57.9 ($\underline{C}$HNHBoc), 79.6 ($CO_2\underline{C}(CH_3)_3$), 128.4 (q, J_{CF} = 276 Hz, CF_3), 156.9 ($\underline{C}O_2C(CH_3)_3$), 172.7 (COOH) ppm; ^{19}F NMR (376 MHz, C_3D_6O): δ = -64.3 (t, J = 11.3 Hz, 3 F, CF_3) ppm; IR (Film): ν = 629 (w), 778 (w), 833 (w), 1050 (m), 1167 (s), 1255 (s), 1370 (s), 1396 (s), 1513 (m), 1714 (vs), 2559 (w), 2942 (w), 2981 (m), 3320 (w) cm^{-1}; HRMS (ESI): *m/z* $[M + Na]^+$ calcd. for $[C_{11}H_{18}F_3NNaO_4]^+$: 308.1080; found: 308.1072.

Fmoc Protection of (2*S*,3*S*)-5,5,5-trifluoroisoleucine, (2*S*)-5,5,5,5',5',5'-hexafluoroleucine and (2*S*)-aminoheptanoic acid

(2*S*,3*S*)-2-{[((9*H*-Fluoren-9-yl)methoxy)carbonyl]amino}-5,5,5-trifluoro-3-methylpentanoic acid

The *N*-Boc protected amino acid **7** (149 mg, 0.522 mmol, 1.00 equiv) was dissolved in CH_2Cl_2/TFA 1:1 (6.0 mL) and stirred at rt for 3 h. The solvent was then removed in vacuo. The remaining residue was dissolved in 10% Na_2CO_3 solution (2.0 mL) and dioxane (1.0 mL) and Fmoc-OSu (192 mg, 0.569 mmol, 1.10 equiv) were added. The mixture was allowed to warm to rt and stirred overnight. The reaction was quenched by the addition of water (30.0 mL). The aqueous layer was washed with diethyl ether (1 × 50 mL). The aqueous layer was acidified with HCl to pH = 2 and extracted with dichloromethane (4 × 50 mL). The combined organic phases were dried over sodium sulfate, filtered, and concentrated in vacuo. The crude product was purified by reversed-phase preparative HPLC using linear CH_3CN/H_2O gradients containing 0.1% trifluoroacetic acid (TFA). Identification was carried out by ESI-ToF MS and NMR spectroscopy. The *N*-Fmoc protected amino acid was obtained as white solid. Yield: 145 mg (0.356 mmol, 68%).

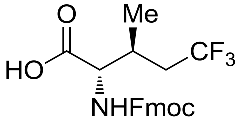

^{1}H NMR (700 MHz, $CDCl_3$): δ = 1.10 (d, *J* = 6.4 Hz, 3 H, CH_3), 2.02-2.09 (m, 1 H, CH_2CF_3), 2.29-2.36 (m, 1 H, CH_2CF_3), 2.39-2.45 (m, 1 H, $C\underline{H}CH_3$), 4.22 (t, *J* = 6.4 Hz, 1 H, $COOCH_2C\underline{H}$), 4.39-4.53 (m, 3 H, $COOC\underline{H}_2CH$, $C\underline{H}CO_2H$), 5.40 (d, *J* = 7.7 Hz, 1 H, NH), 7.30-7.34 (m, 2 H, Ar), 7.39-7.42 (m, 2 H, Ar), 7.58 (d, *J* = 7.8 Hz, 2 H, Ar), 7.76 (d, *J* = 7.4 Hz, 2 H, Ar) ppm signal for COOH was not found; ^{13}C NMR (176 MHz, $CDCl_3$): δ = 16.0 (CH_3), 31.2 (m, $\underline{C}HCH_3$), 36.5 (q, *J* = 28.7 Hz, $\underline{C}H_2CF_3$), 47.2 ($COOCH_2\underline{C}H$), 57.8 ($\underline{C}HCO_2H$), 67.2 ($COO\underline{C}H_2CH$), 120.1 (Ar-C), 125.0 (Ar-C), 126.6 (q, *J* = 276 Hz, CF_3), 127.1 (Ar-C), 127.8 (Ar-C), 141.3 (Ar-C), 143.5 (Ar-C), 156.1 ($\underline{C}OOCH_2$), 174.9 (COOH) ppm; ^{19}F NMR (376 MHz, $CDCl_3$): δ = -63.6 (t, *J* = 10.6 Hz, 3 F, CF_3) ppm; HRMS (ESI): *m/z* $[M + Na]^+$ calcd. for $[C_{21}H_{20}F_3NNaO_4]^+$: 430.1237; found *m/z*: 430.1246.

(*S*)-2-{[((9*H*-Fluoren-9-yl)methoxy)carbonyl]amino}-5,5,5-trifluoro-4-(trifluoromethyl)-pentanoic acid

(2*S*)-Hexafluoroleucine (148 mg, 0.619 mmol, 1.00 equiv) was dissolved in 10% Na_2CO_3 solution (2.0 mL) and dioxane (1.0 mL) and Fmoc-OSu (162 mg, 0.480 mmol, 1.10 equiv) were added. The mixture was allowed to warm to rt and stirred overnight. The reaction was quenched by the addition of water (30.0 mL). The aqueous layer was washed with diethyl ether (1 × 50 mL). The aqueous layer was acidified with HCl to pH = 2 and extracted with

dichloromethane (4 × 50 mL). The combined organic phases were dried over sodium sulfate, filtered, and concentrated in vacuo. The crude product was purified by reversed-phase preparative HPLC using linear CH_3CN/H_2O gradients containing 0.1% trifluoroacetic acid (TFA). Identification was carried out by ESI-ToF MS and NMR spectroscopy. The *N*-Fmoc protected amino acid was obtained as white solid. Yield: 140 mg (0.303 mmol, 48%).

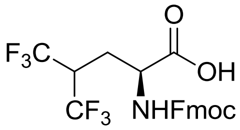

^{1}H NMR (700 MHz, $CDCl_3$): δ = 2.07-2.14 (m, 1 H, C<u>H</u>$_2$CH(CF_3)$_2$), 2.42-2.49 (m, 1 H, C<u>H</u>$_2$CH(CF_3)$_2$), 3.11-3.21 (m, 1 H, CH(CF_3)$_2$), 4.21 (t, *J* = 6.4 Hz, 1 H, $COOCH_2$C<u>H</u>), 4.41-4.68 (m, 3 H, C<u>H</u>CO_2H, COOC<u>H</u>$_2$CH), 5.34 (br s, 1 H, NH), 7.28-7.33 (m, 2 H, Ar), 7.38-7.42 (m, 2 H, Ar), 7.56 (d, *J* = 6.9 Hz, 2 H, Ar), 7.76 (d, *J* = 9.1 Hz, 2 H, Ar) ppm signal for COOH was not found; ^{13}C NMR (176 MHz, $CDCl_3$): δ = 26.7 (m, <u>C</u>H$_2$CH(CF_3)$_2$), 44.3 (m, <u>C</u>H(CF_3)$_2$), 47.1 ($COOCH_2$<u>C</u>H), 51.6 (<u>C</u>HCO_2H), 67.4 (COO<u>C</u>H$_2$CH), 120.1 (Ar-C), 125.0 (Ar-C), 127.1 (Ar-C), 127.9 (Ar-C), 141.4 (Ar-C), 143.3 (Ar-C), 156.4 (<u>C</u>OOCH$_2$), 174.6 (COOH) ppm signal for CF_3 was not found; ^{19}F NMR (376 MHz, $CDCl_3$): δ = -67.3 (m, 3 F, CF_3), -67.58 (m, 3 F, CF_3) ppm; HRMS (ESI): *m/z* $[M + Na]^+$ calcd. for $[C_{21}H_{17}F_6NNaO_4]^+$ 484.0954; found *m/z*: 484.0958.

(*S*)-2-{[((9*H*-Fluoren-9-yl)methoxy)carbonyl]amino}heptanoic acid

The unprotected aminoheptanoic acid derivative (100 mg, 0.689 mmol, 1.00 equiv) was dissolved in 10% Na_2CO_3 solution (2.0 mL) and dioxane (1.0 mL) and Fmoc-OSu (256 mg, 0.759 mmol, 1.10 equiv) was added. The mixture was allowed to warm to rt and stirred overnight. The reaction was quenched by the addition of water (30 mL). The aqueous layer was washed with diethyl ether (1 × 50 mL). The aqueous layer was acidified with HCl to pH = 2 and extracted with dichloromethane (4 × 50 mL). The combined organic phases were dried over sodium sulfate, filtered, and concentrated in vacuo. The crude product was purified by reversed-phase preparative HPLC using linear CH_3CN/H_2O gradients containing 0.1% trifluoroacetic acid (TFA). Identification was carried out by ESI-ToF MS and NMR spectroscopy. The *N*-Fmoc protected amino acid was obtained as white solid. Yield: 165 mg (0.449 mmol, 65%).

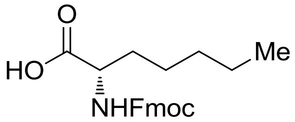

^{1}H NMR (500 MHz, C_3D_6O): δ = 0.88-0.91 (m, 3 H, CH_3), 1.28-1.38 (m, 4 H, (C<u>H</u>$_2$)$_2$$CH_3$), 1.42-1.50 (m, 2 H, C<u>H</u>$_2$$CH_2CHCO_2$H), 1.70-1.78 (m, 1 H, CH_2C<u>H</u>$_2$CHCO_2H), 1.84-1.91 (m, 1 H, CH_2C<u>H</u>$_2$CHCO_2H), 4.21-4.27 (m, 2 H, $COOCH_2$C<u>H</u>), C<u>H</u>CO_2H), 4.33 (d, *J* = 7.2 Hz, 2 H, COOC<u>H</u>$_2$CH), 7.30-7.34 (m, 2 H, Ar), 7.39-7.42 (m, 2 H, Ar), 7.71-7.73 (m, 2 H, Ar), 7.86 (d, *J* = 7.5 Hz, 2 H, Ar) ppm signals for NH and COOH were not found; ^{13}C NMR (126 MHz,

C_3D_6O): δ = 14.3 (CH_3), 23.1 ($CH_2\underline{C}H_2CH_3$), 26.2 ($\underline{C}H_2CH_2CHCO_2H$), 32.1 ($\underline{C}H_2CH_2CH_3$), 32.5 ($\underline{C}H_2CHCO_2H$), 48.0 ($COOCH_2\underline{C}H$), 54.7 ($\underline{C}HCO_2H$), 67.1 ($COO\underline{C}H_2CH$), 120.8 (Ar-C), 126.2 (Ar-C), 127.9 (Ar-C), 128.5 (Ar-C), 142.1 (Ar-C), 145.0 (Ar-C), 157.1 ($\underline{C}OOCH_2$), 174.1 (COOH) ppm; HRMS (ESI): *m/z* $[M + Na]^+$ calcd. for $[C_{22}H_{25}NNaO_4]^+$: 390.1676; found: 390.1684.

X-Ray Crystal Structure Analysis

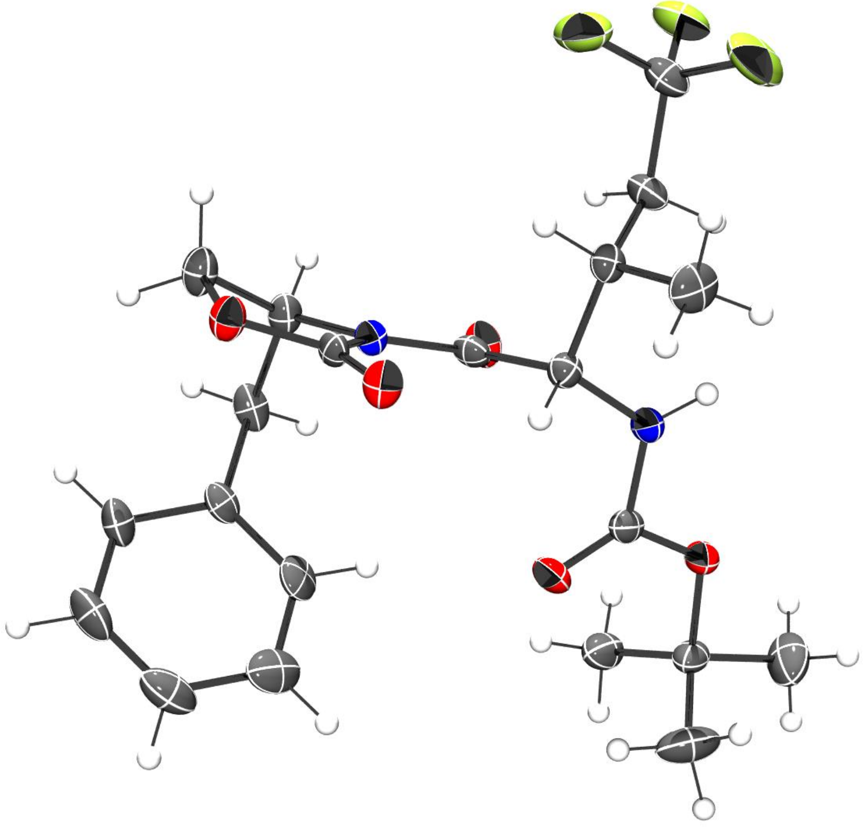

Molecular structure ORTEP[4] of protected 5'-F_3-Ile intermediate **6**.

[4] ORTEP-3 for Windows: Farrugia, L. J. *J. Appl. Crystallogr.* **1997**, *30*, 565.

Compound **2**

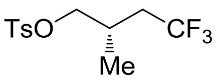

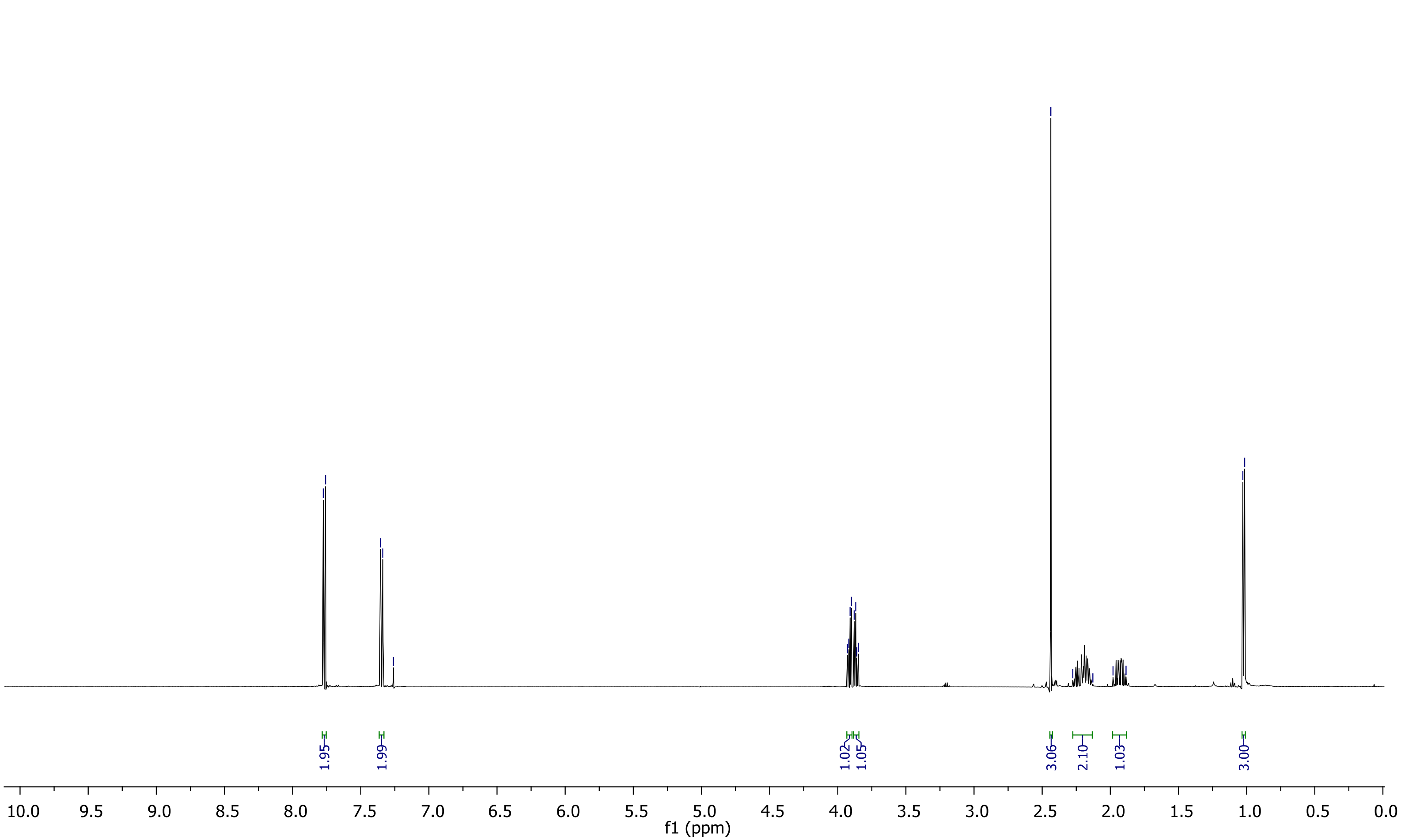

^{1}H NMR (500 MHz, $CDCl_3$)

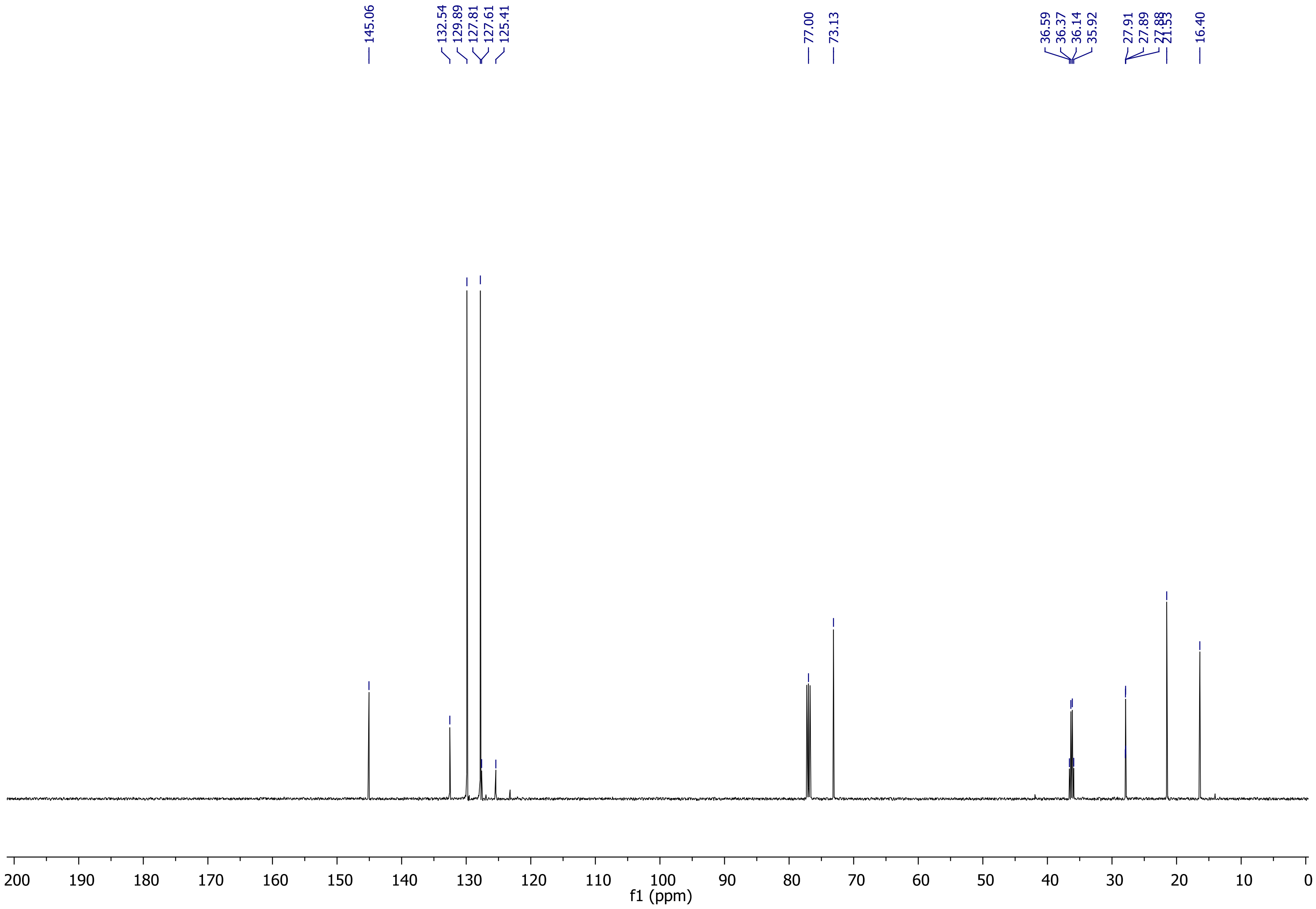

^{13}C NMR (126 MHz, $CDCl_3$)

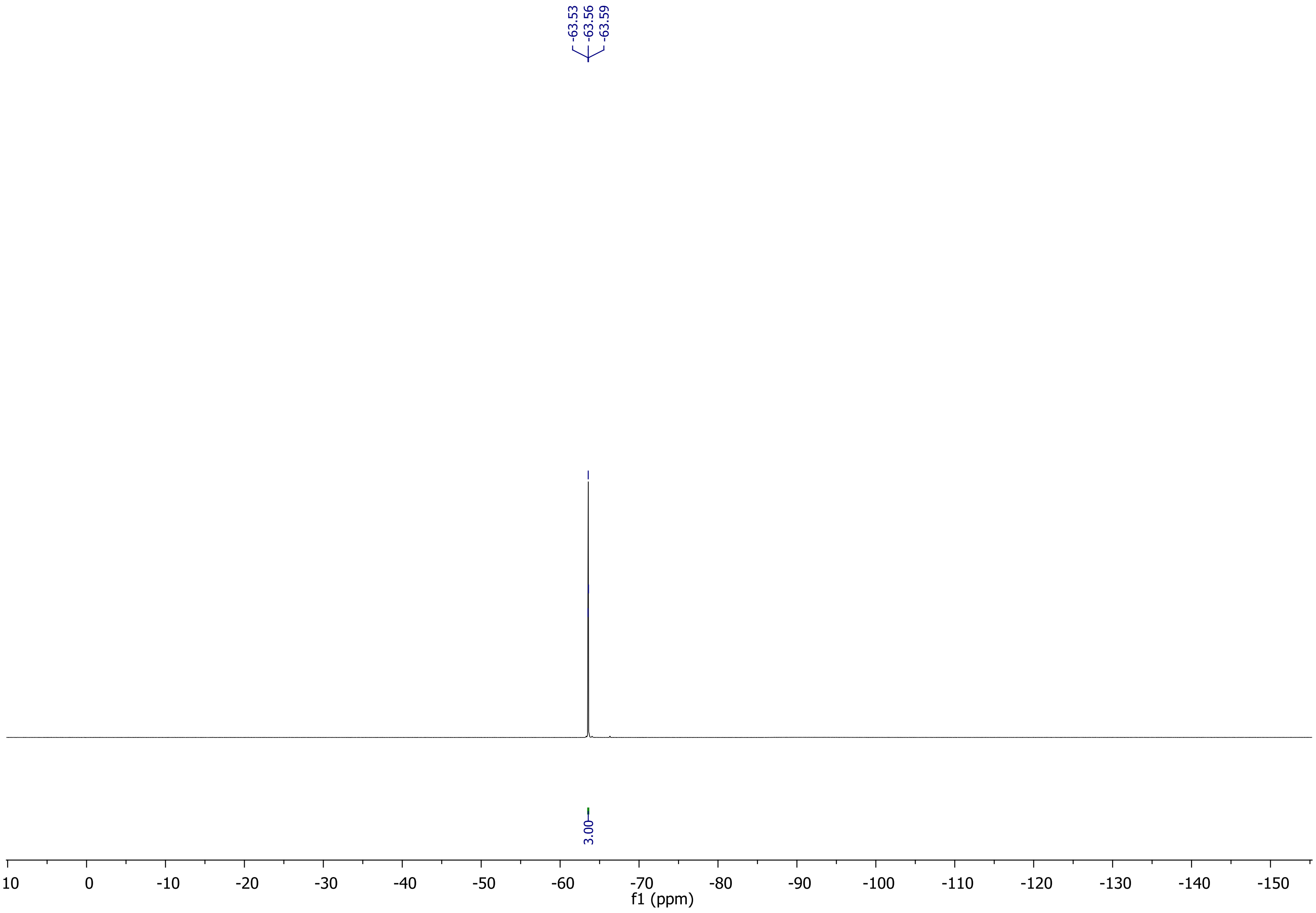

^{19}F NMR (376 MHz, $CDCl_3$)

Compound **3**

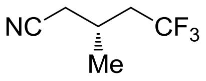

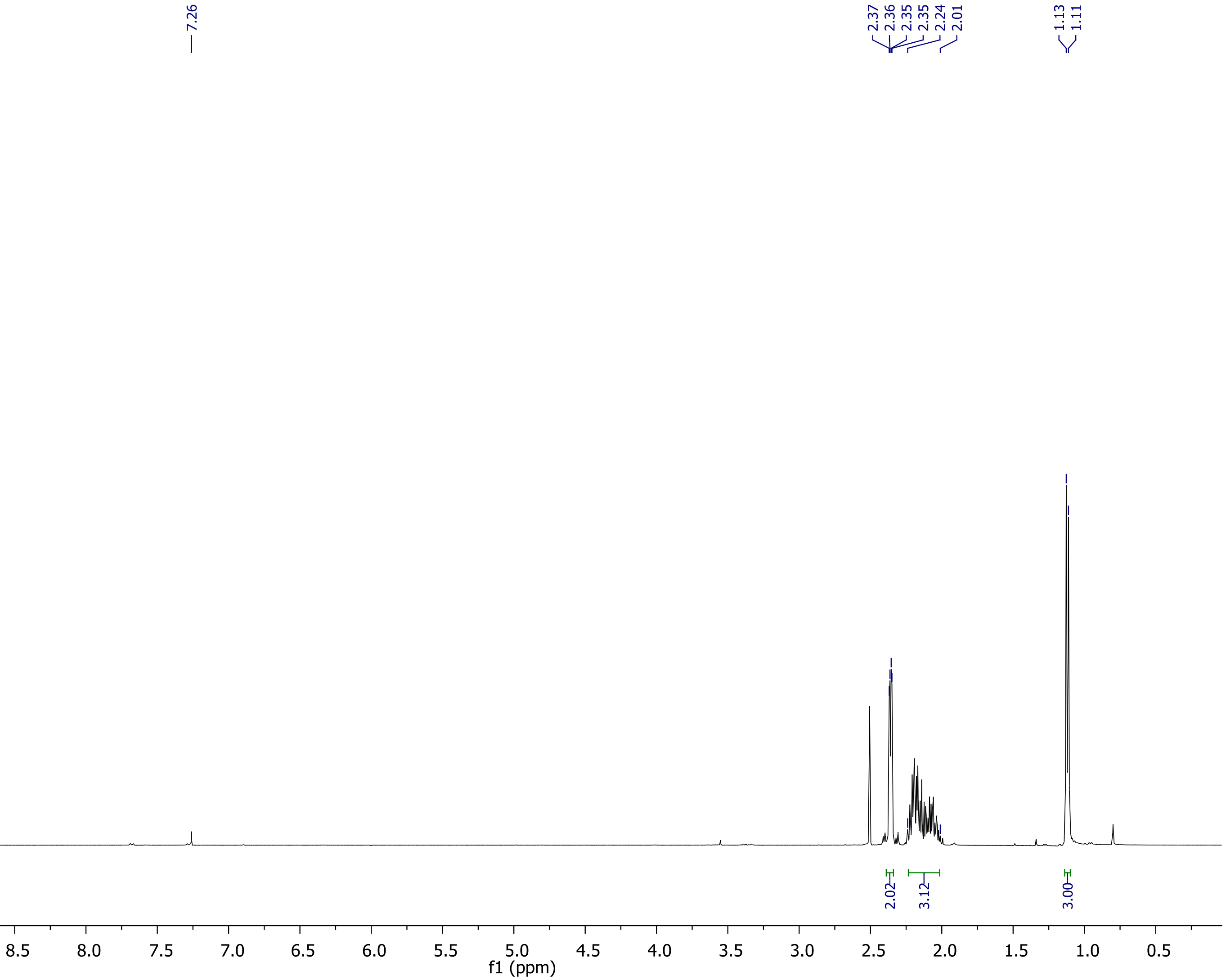

^{1}H NMR (400 MHz, $CDCl_3$)

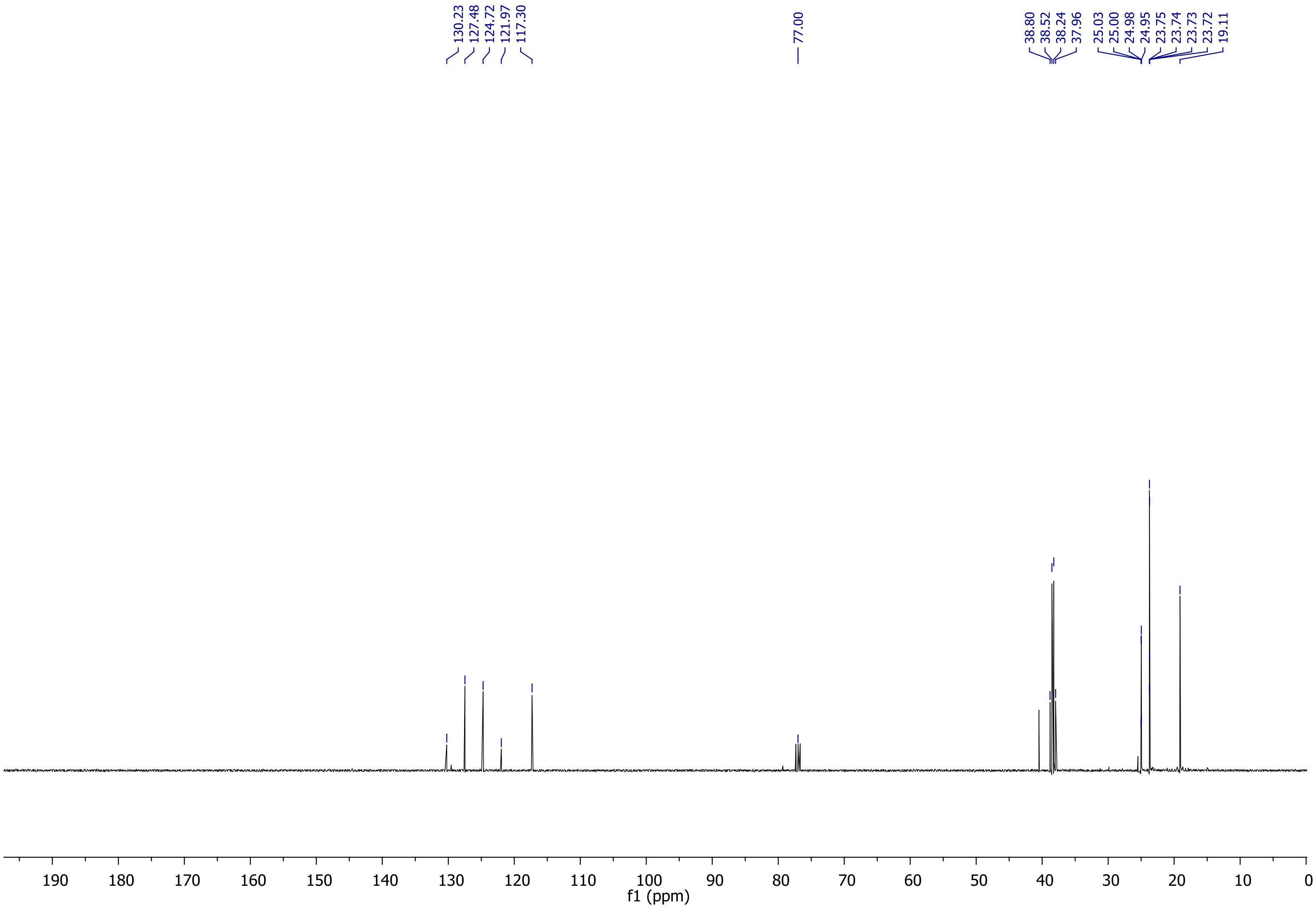

^{13}C NMR (101 MHz, $CDCl_3$)

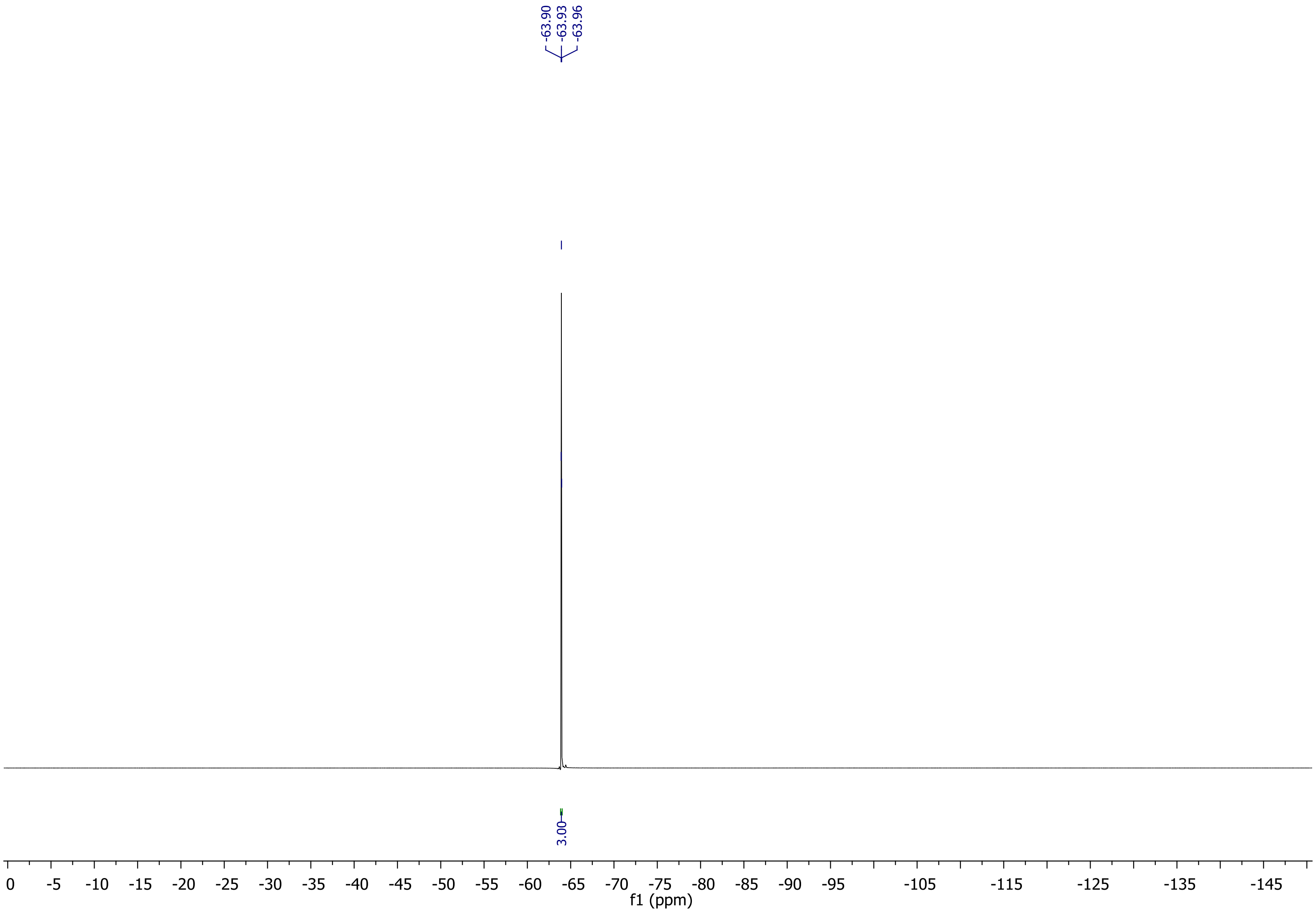

^{19}F NMR (376 MHz, $CDCl_3$)

Compound **4**

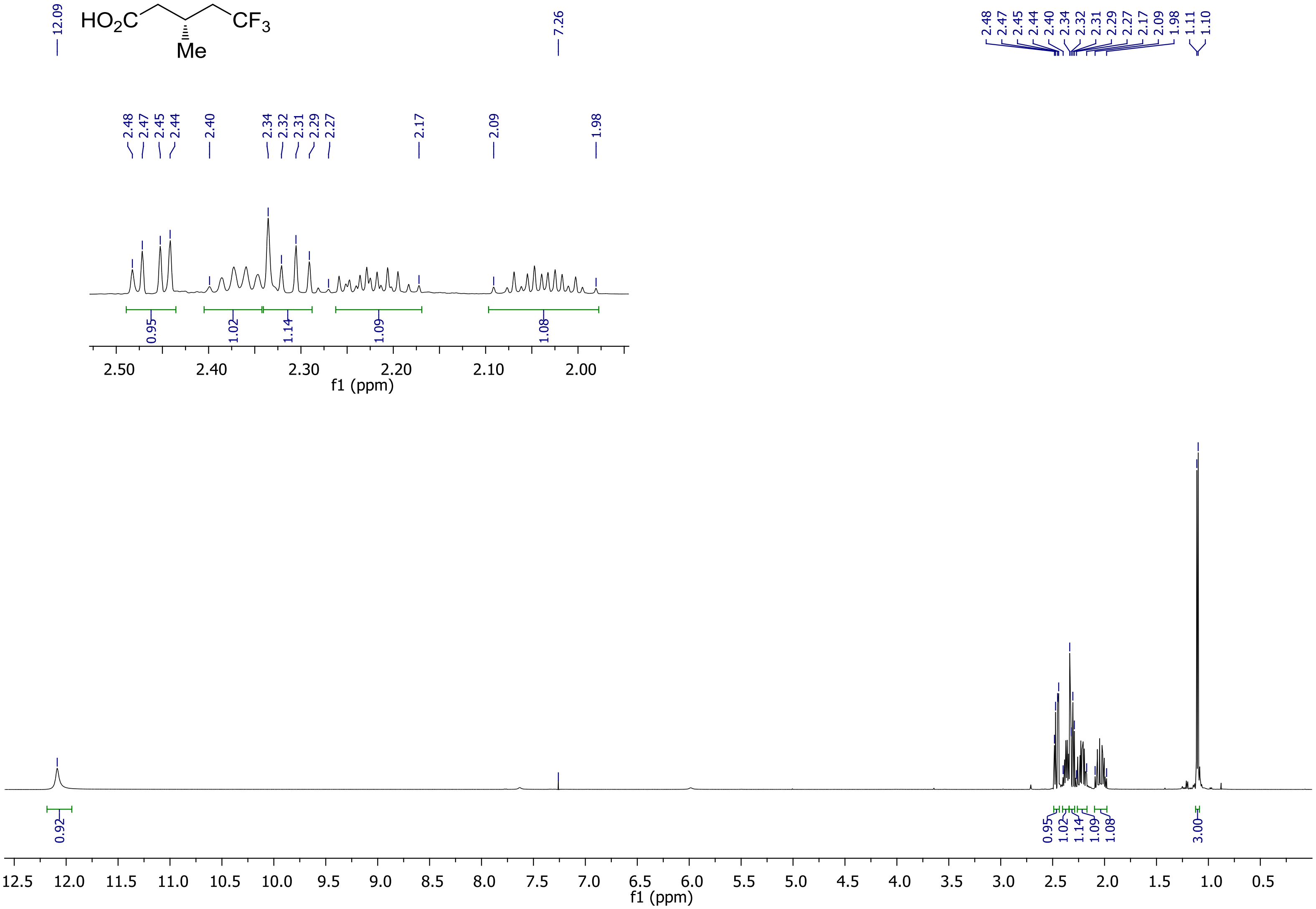

^{1}H NMR (500 MHz, $CDCl_3$)

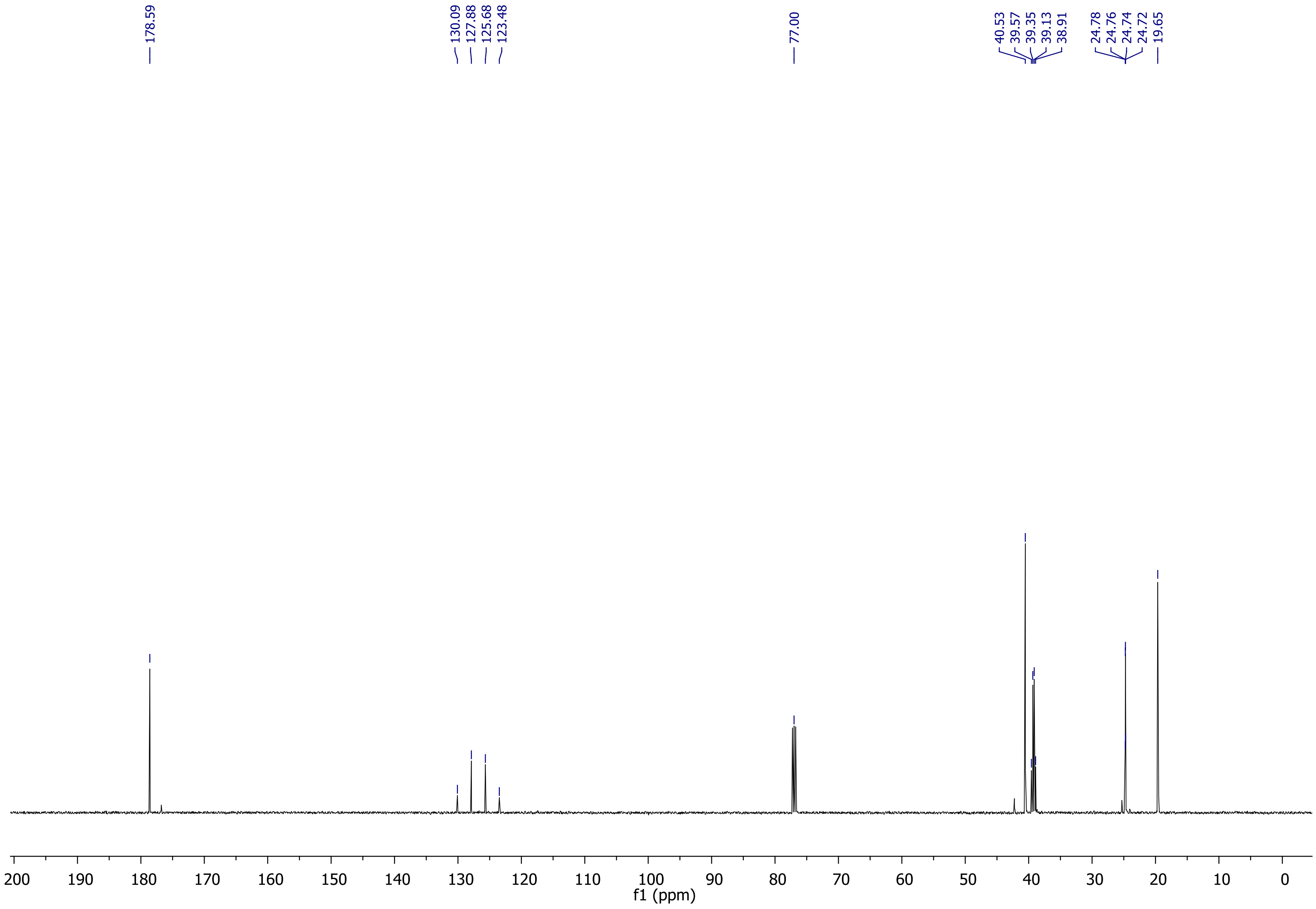

^{13}**C NMR** (126 MHz, $CDCl_3$)

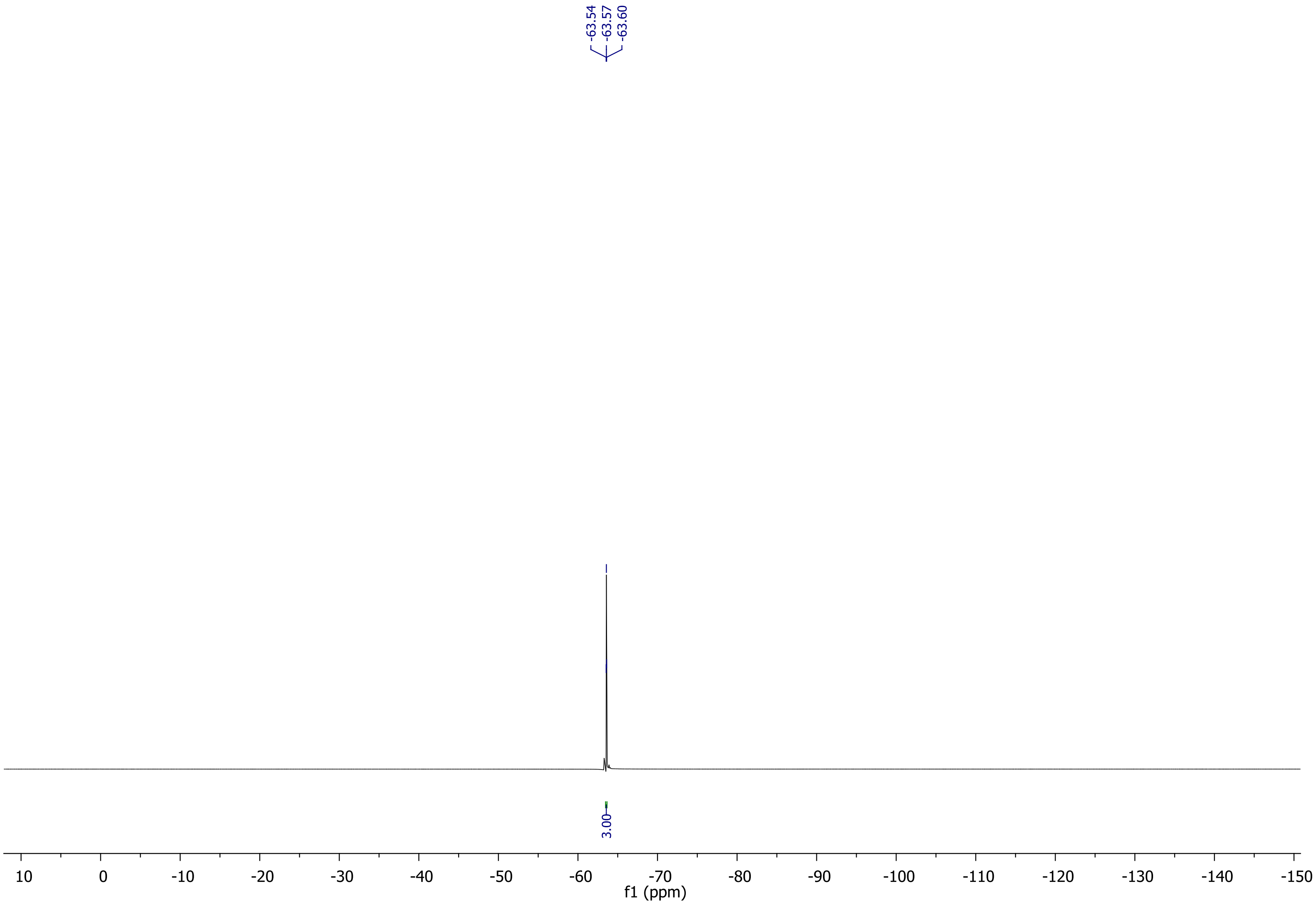

^{19}F NMR (376 MHz, $CDCl_3$)

Compound **5**

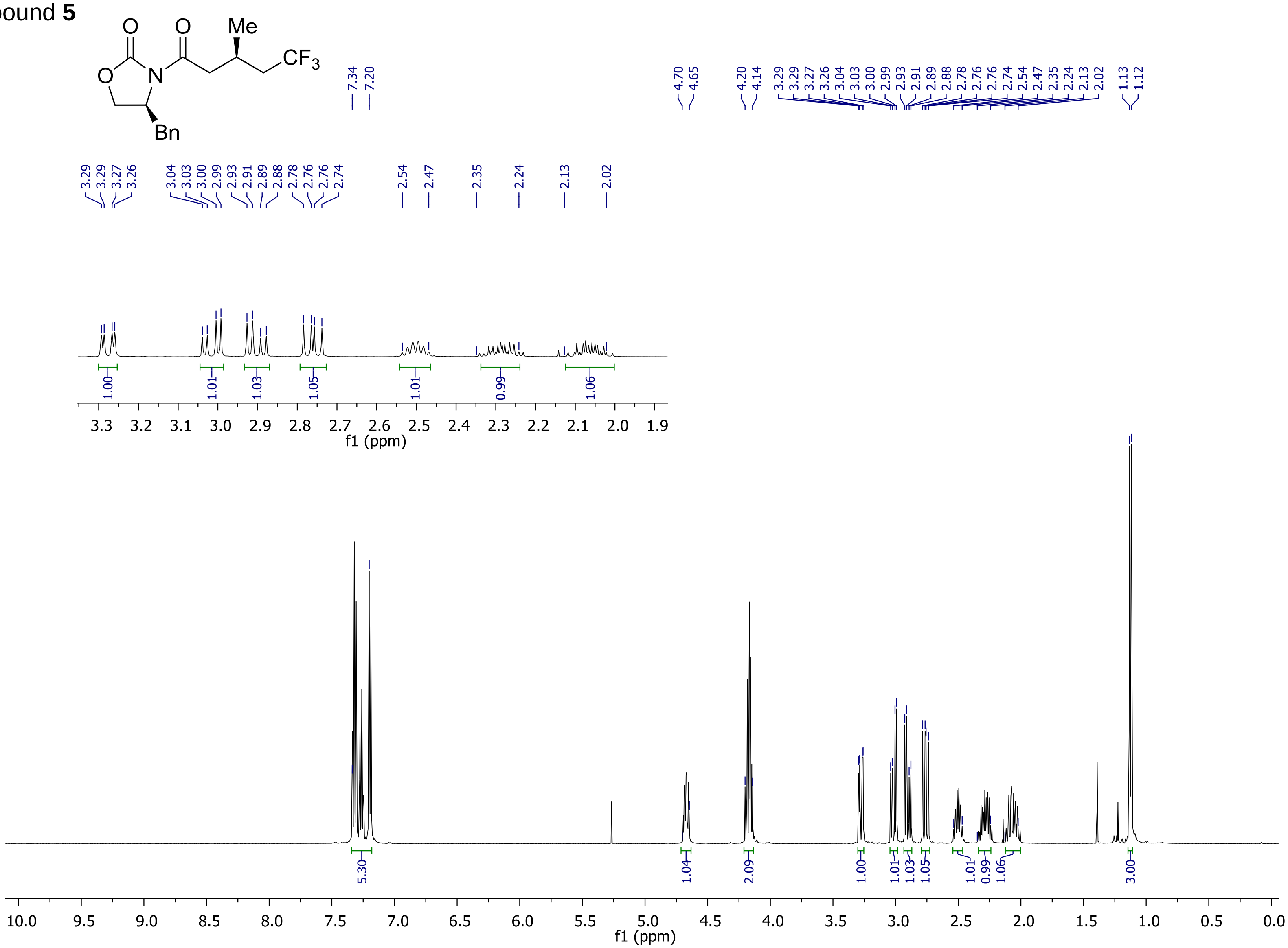

^{1}H NMR (500 MHz, $CDCl_3$)

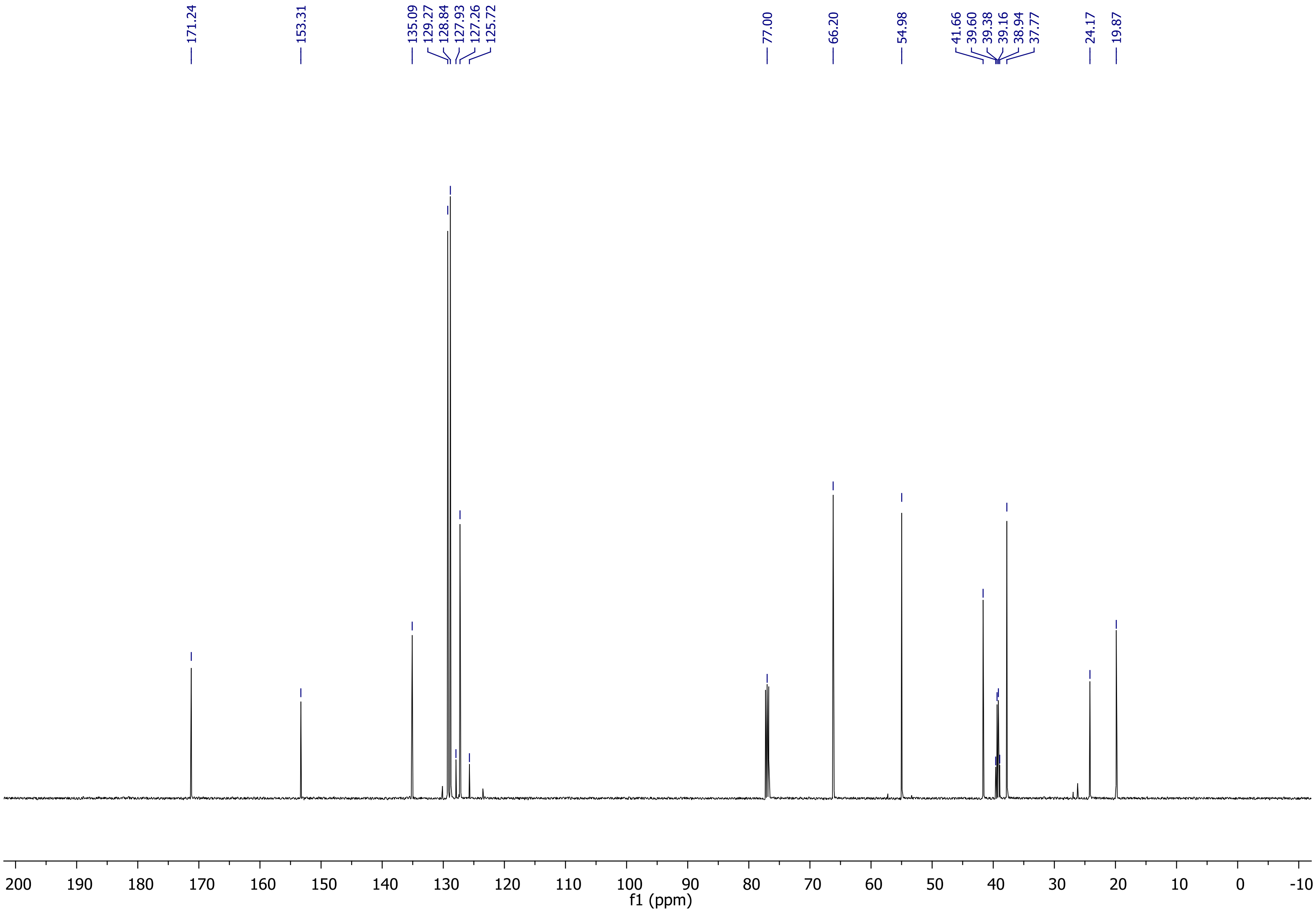

^{13}C NMR (126 MHz, $CDCl_3$)

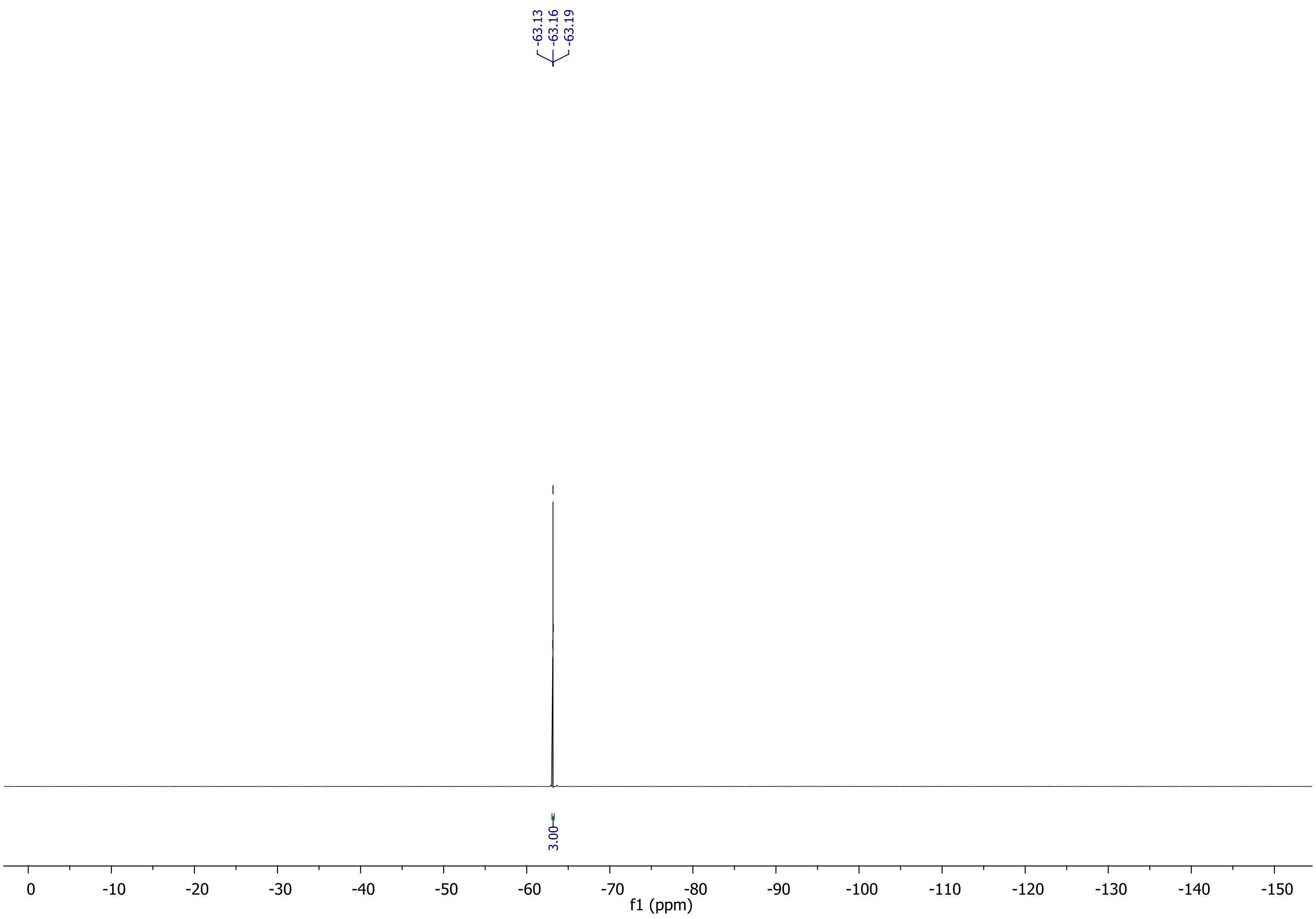

^{19}F NMR (376 MHz, $CDCl_3$)

Compound **5'**

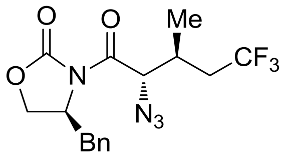

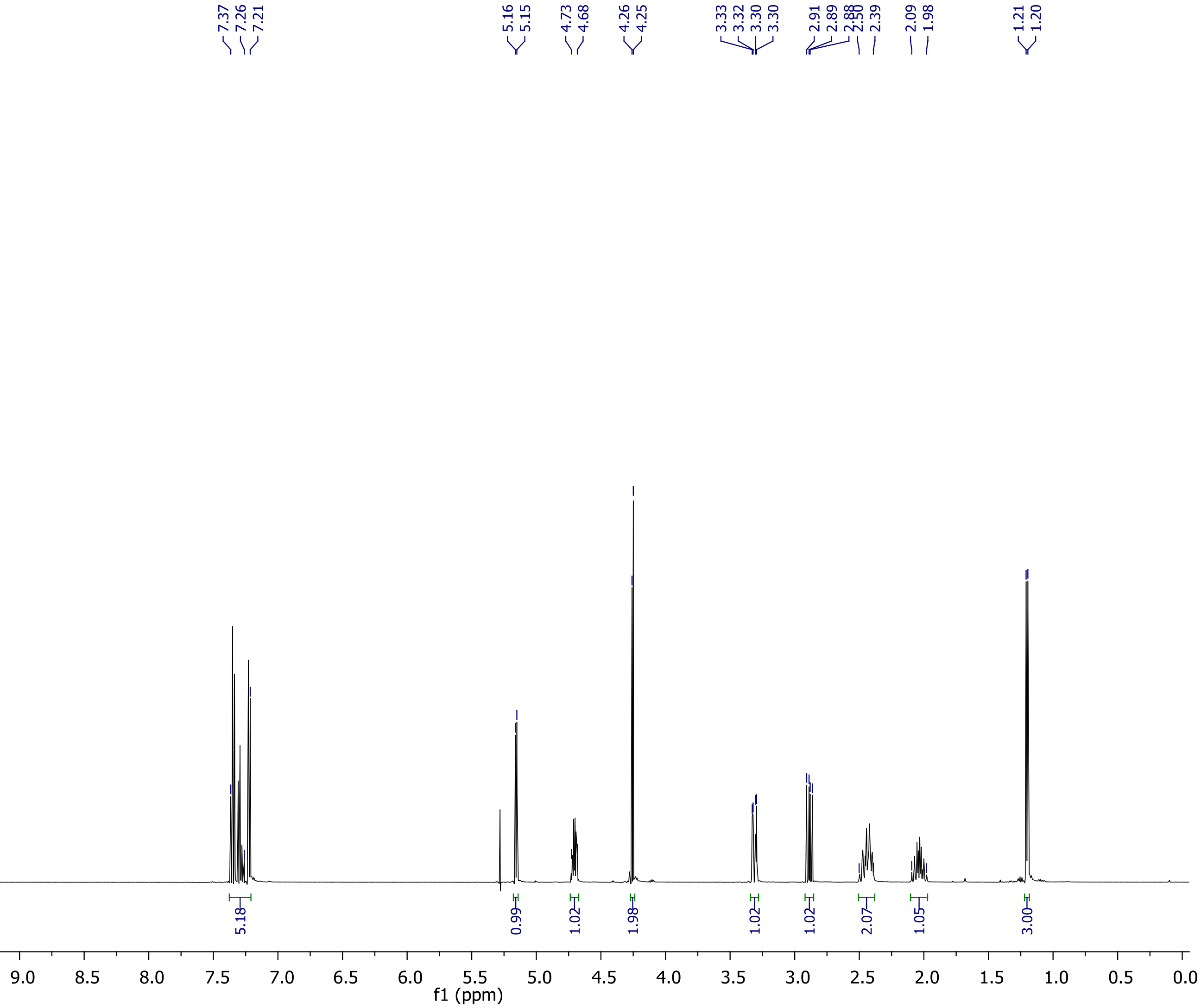

^{1}H NMR (500 MHz, $CDCl_3$)

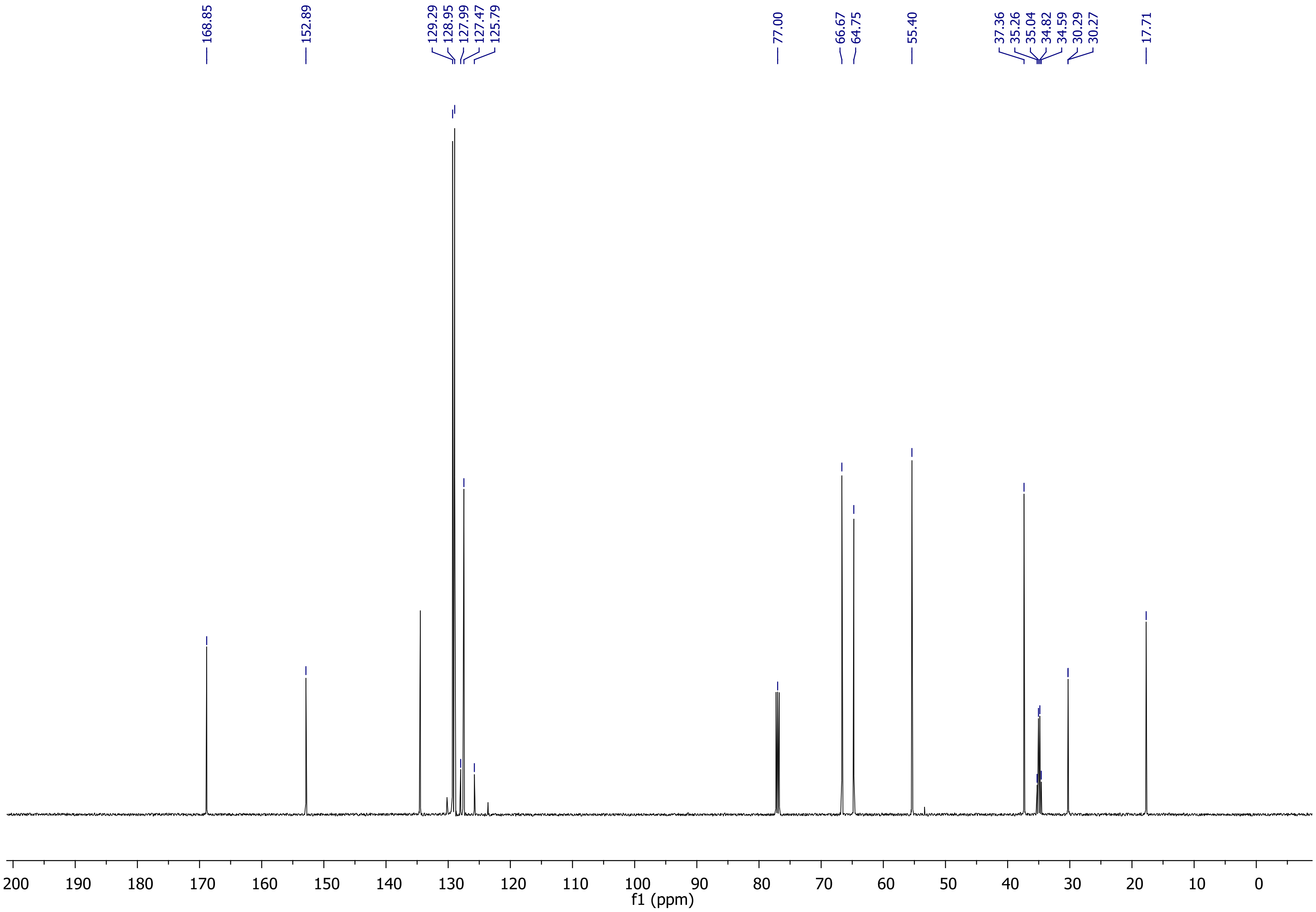

^{13}C NMR (126 MHz, $CDCl_3$)

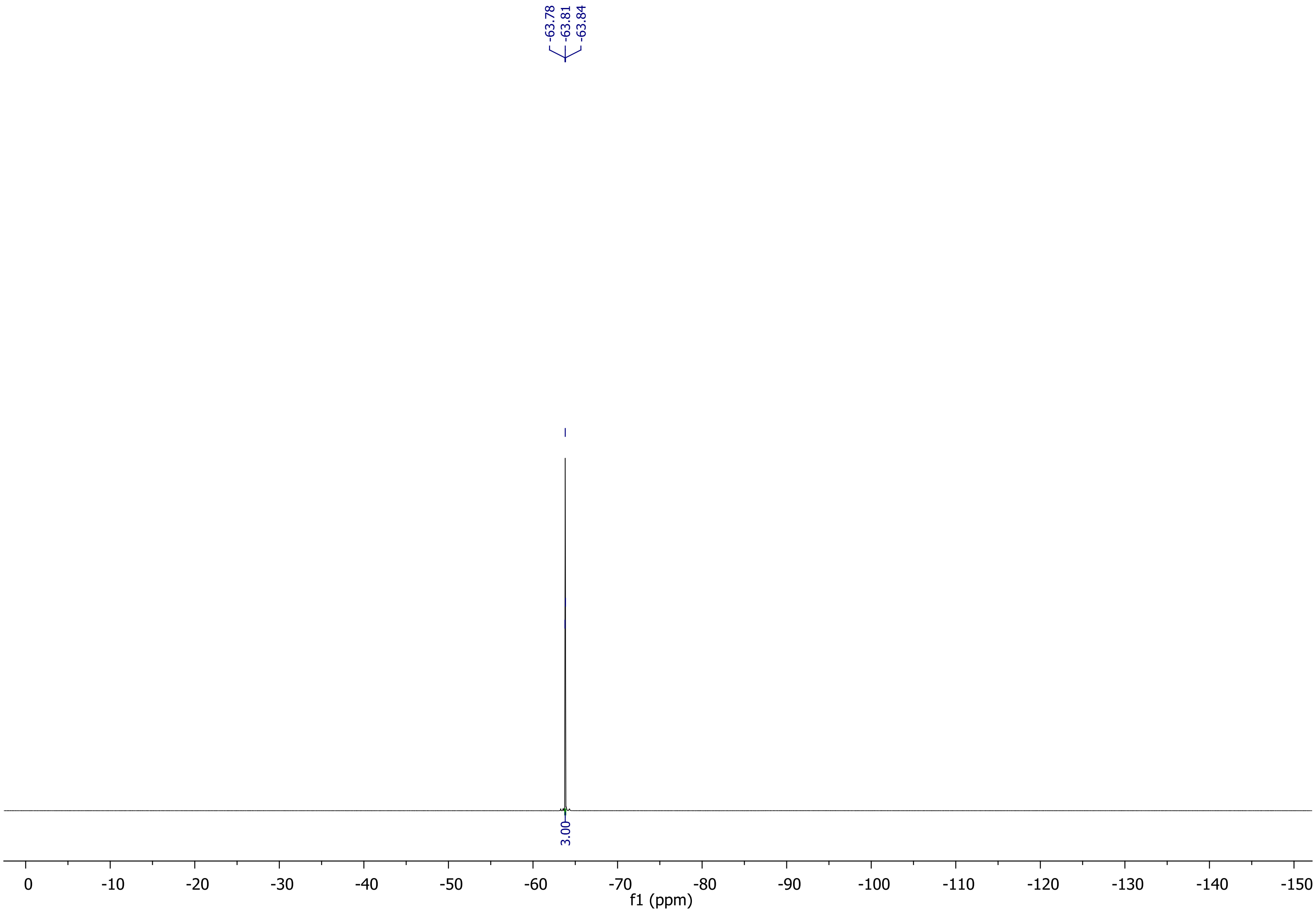

^{19}**F NMR** (376 MHz, $CDCl_3$)

Compound **9**

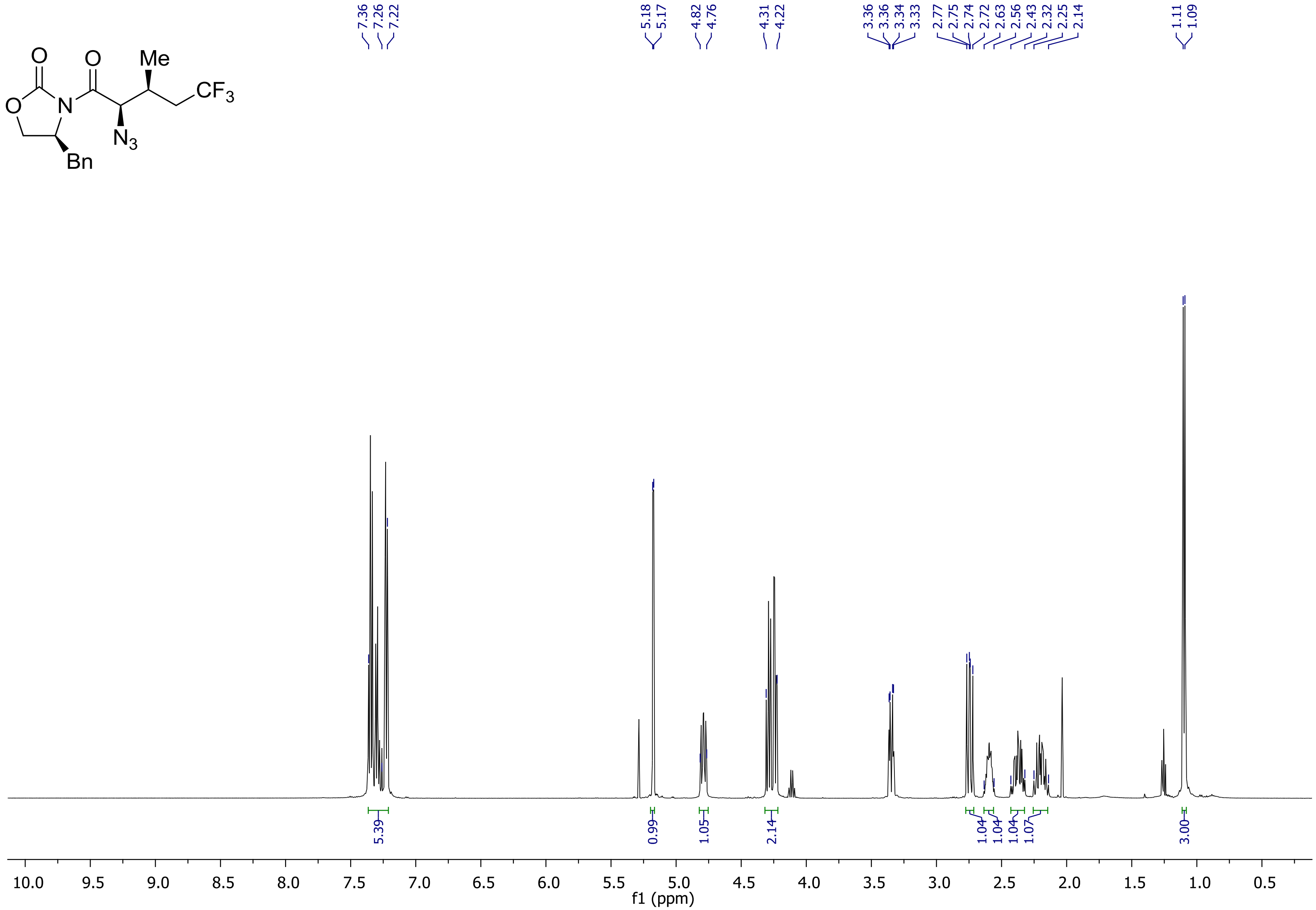

^{1}H NMR (500 MHz, $CDCl_3$)

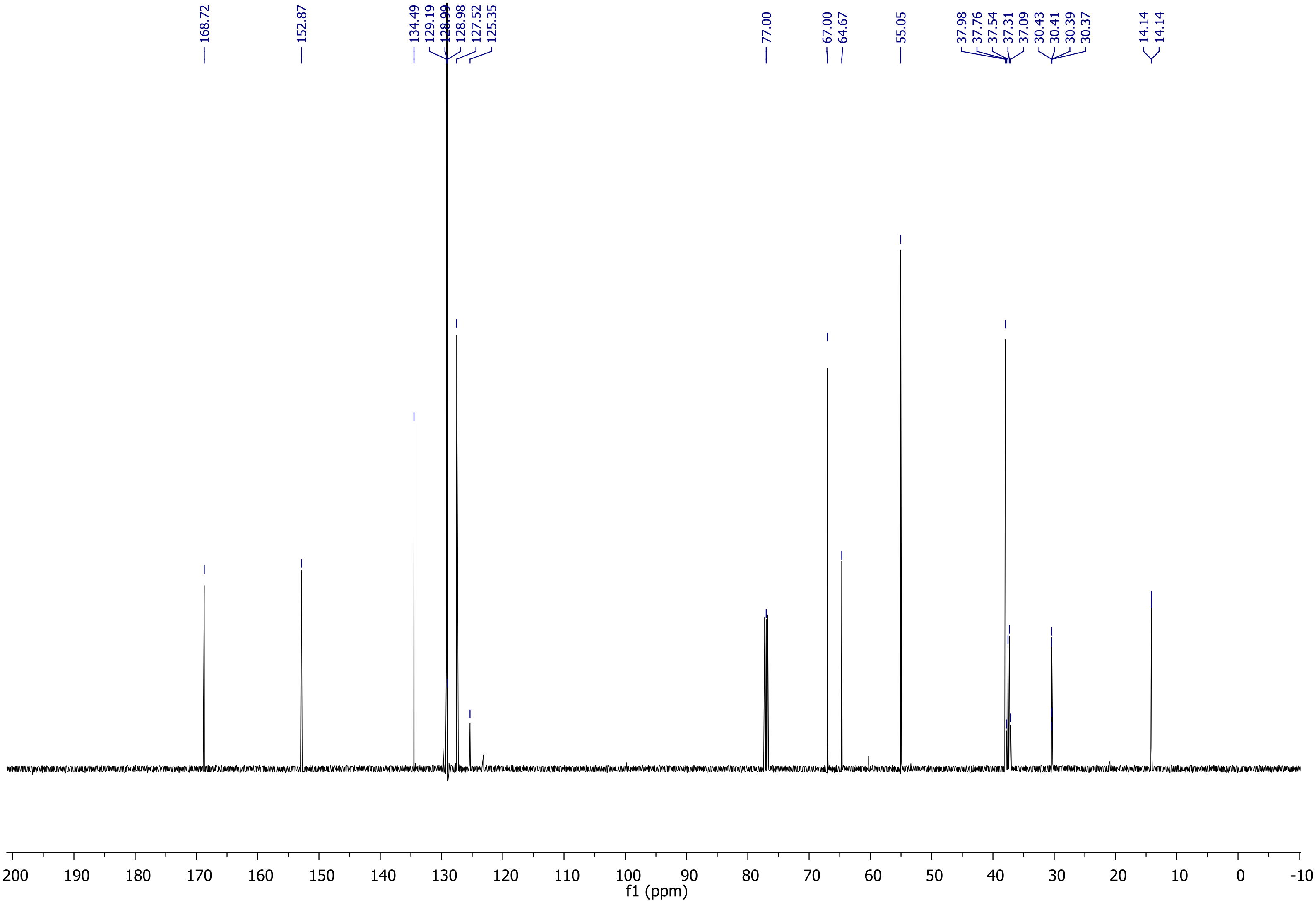

^{13}C NMR (126 MHz, $CDCl_3$)

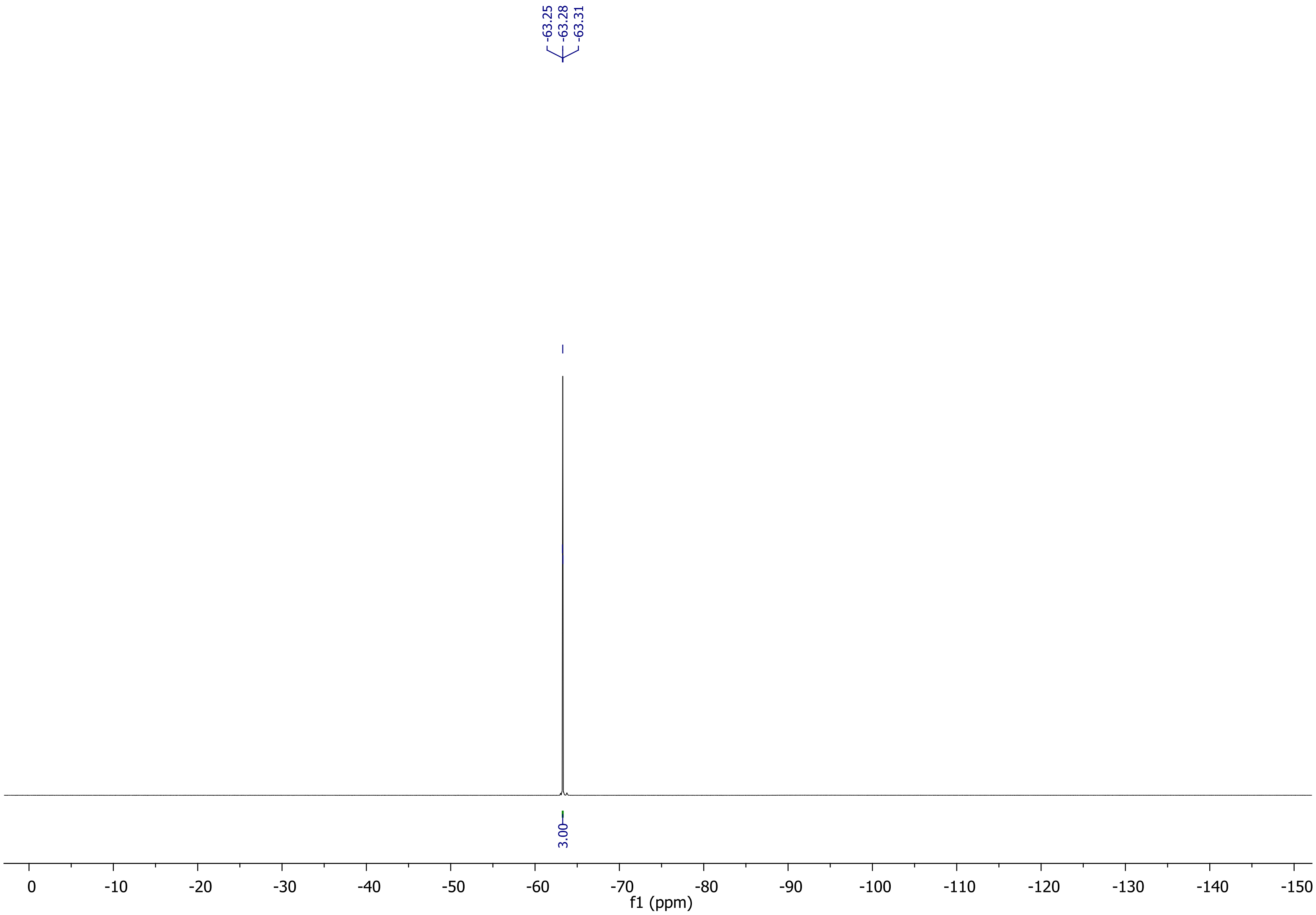

^{19}F NMR (376 MHz, $CDCl_3$)

Compound **8a**

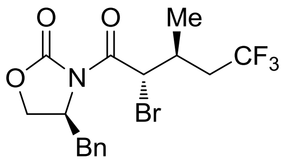

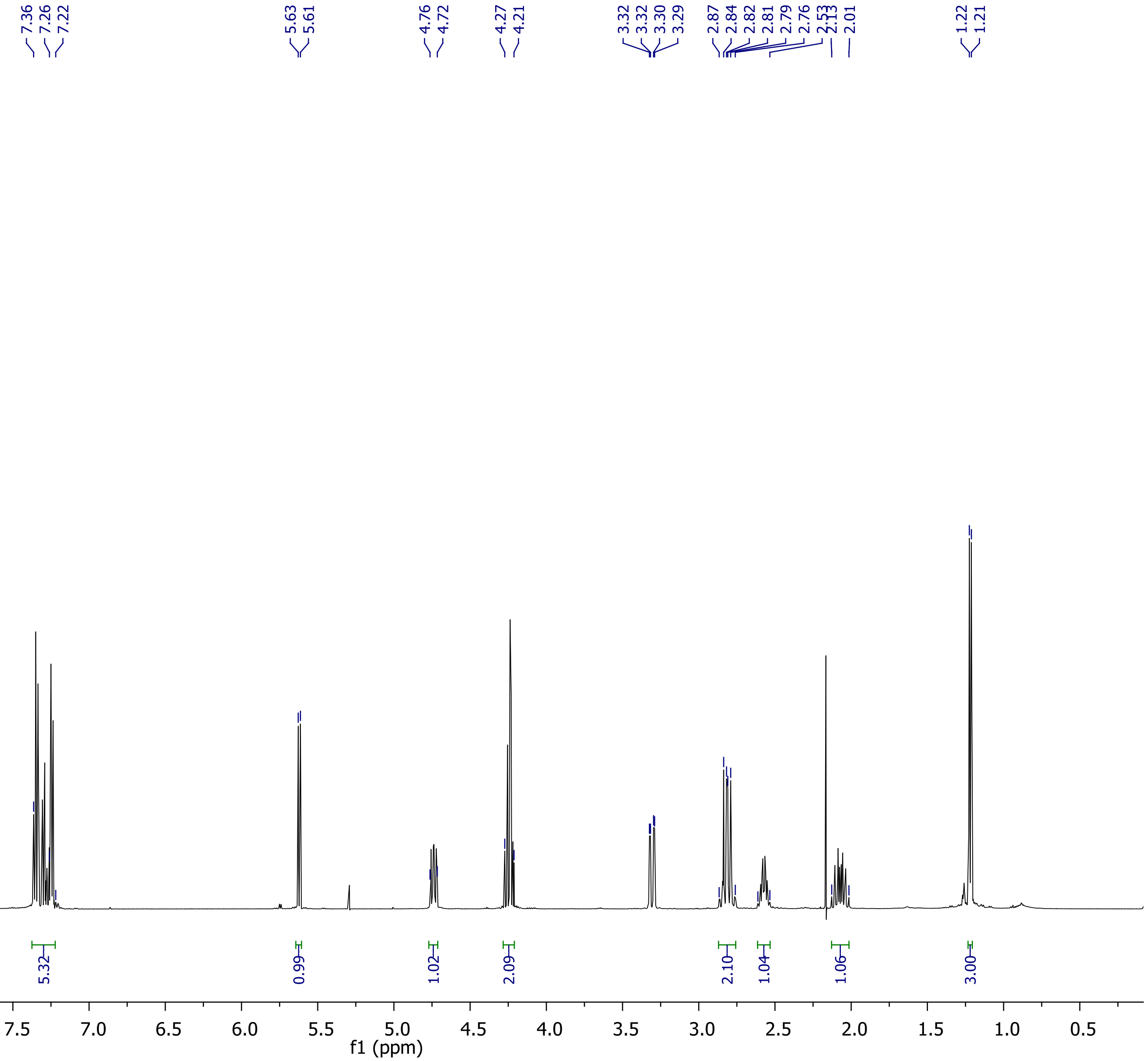

^{1}H NMR (500 MHz, $CDCl_3$)

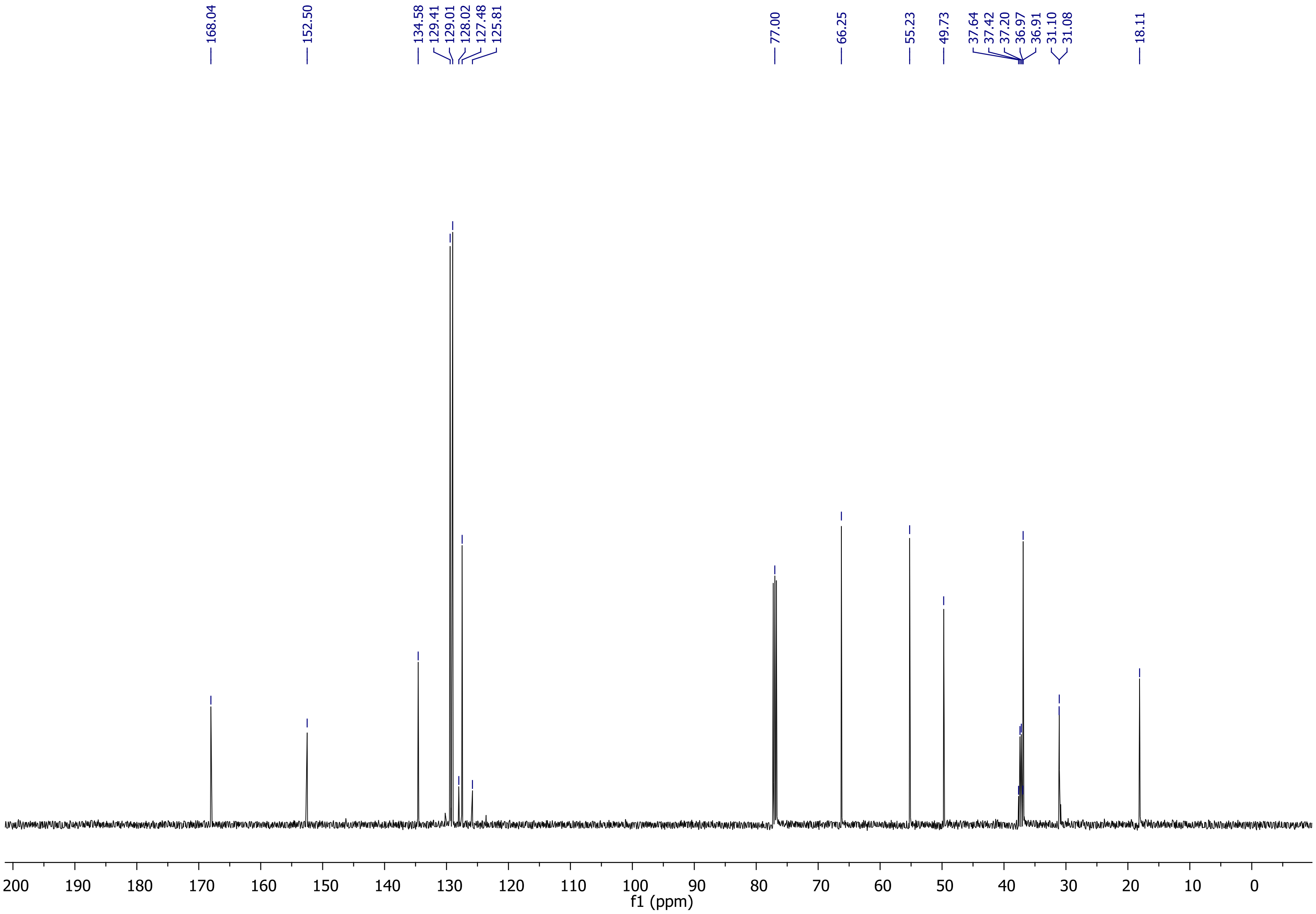

^{13}C NMR (126 MHz, $CDCl_3$)

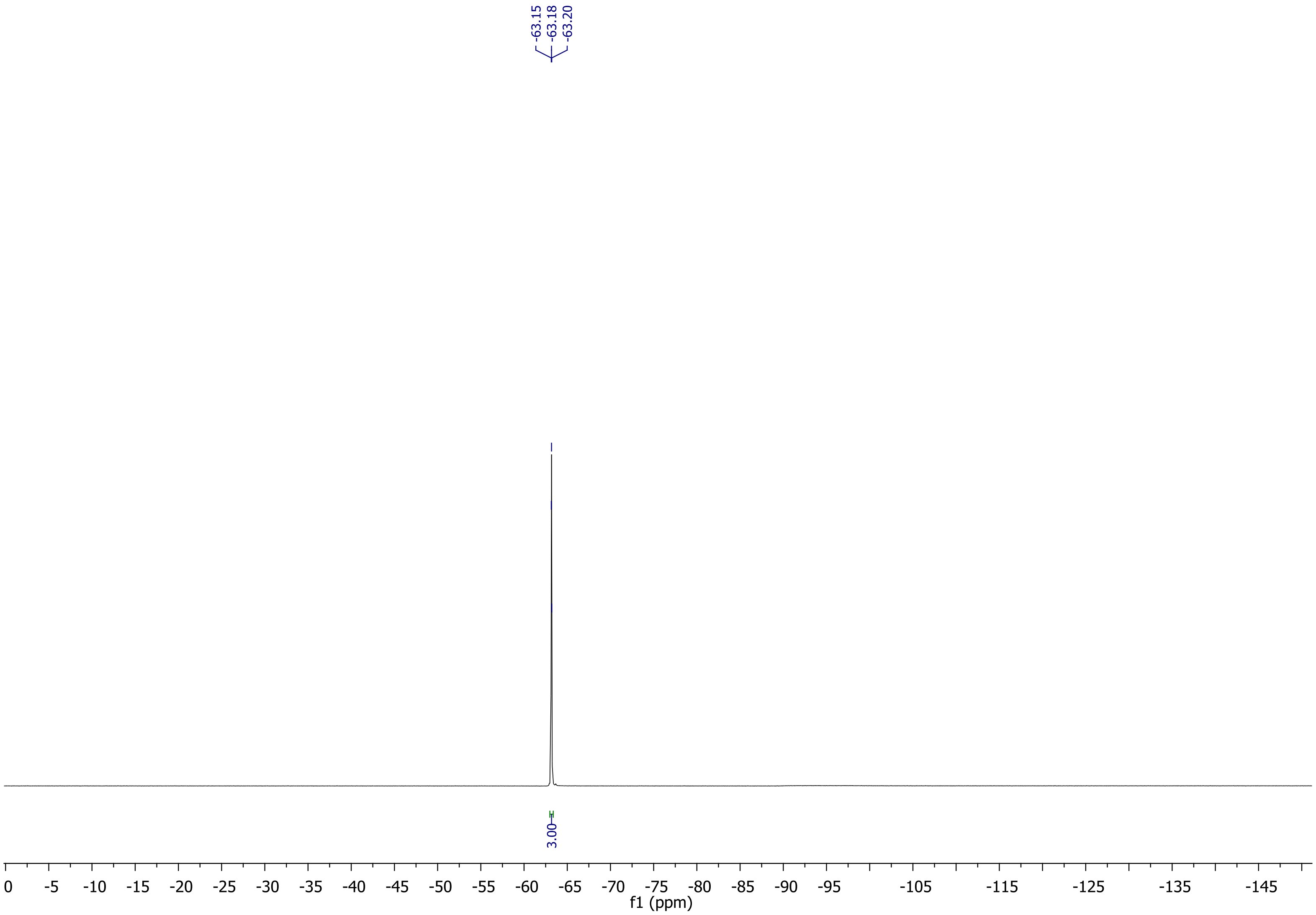

^{19}F NMR (376 MHz, $CDCl_3$)

Compound **8b**

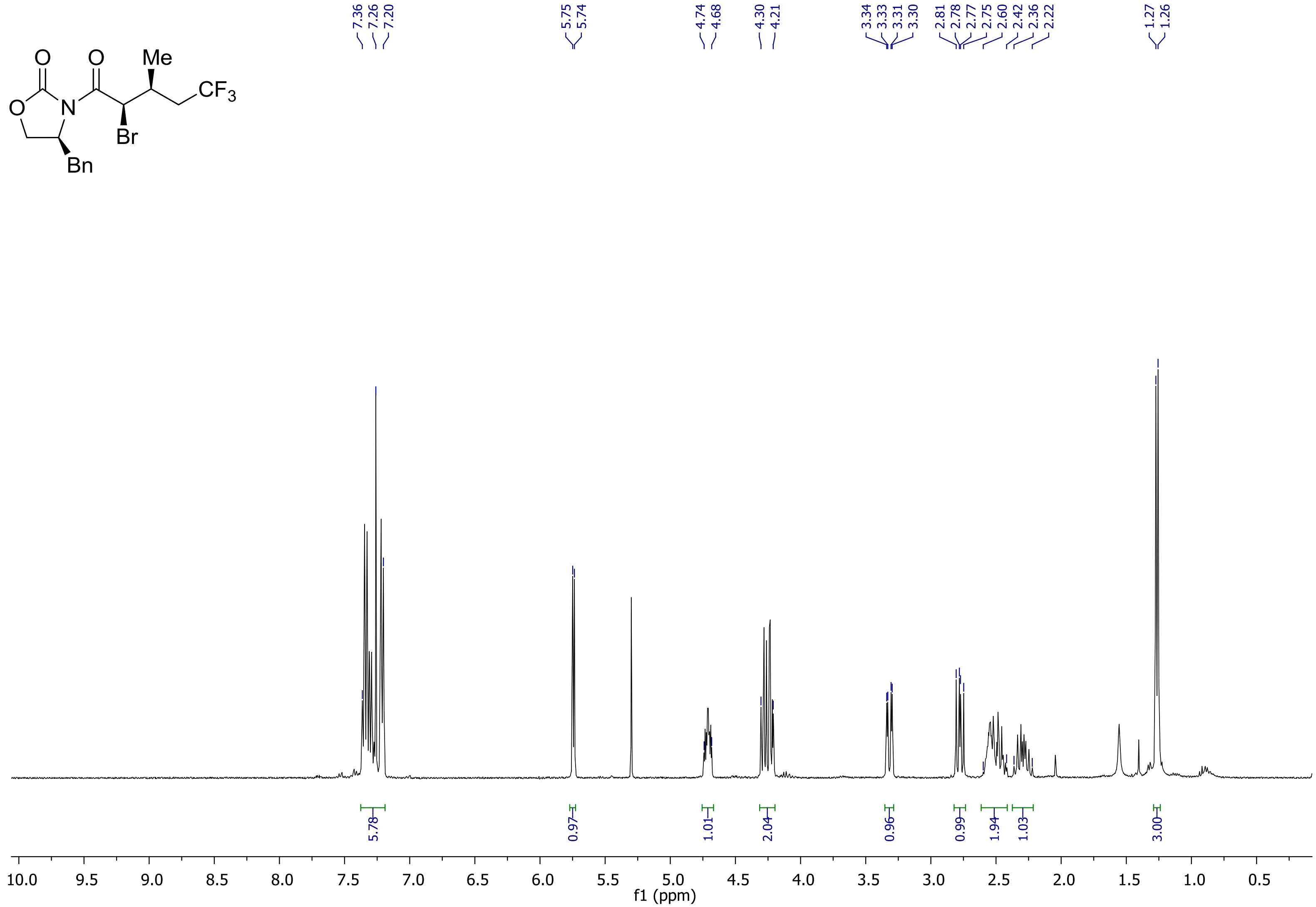

^{1}H NMR (400 MHz, $CDCl_3$)

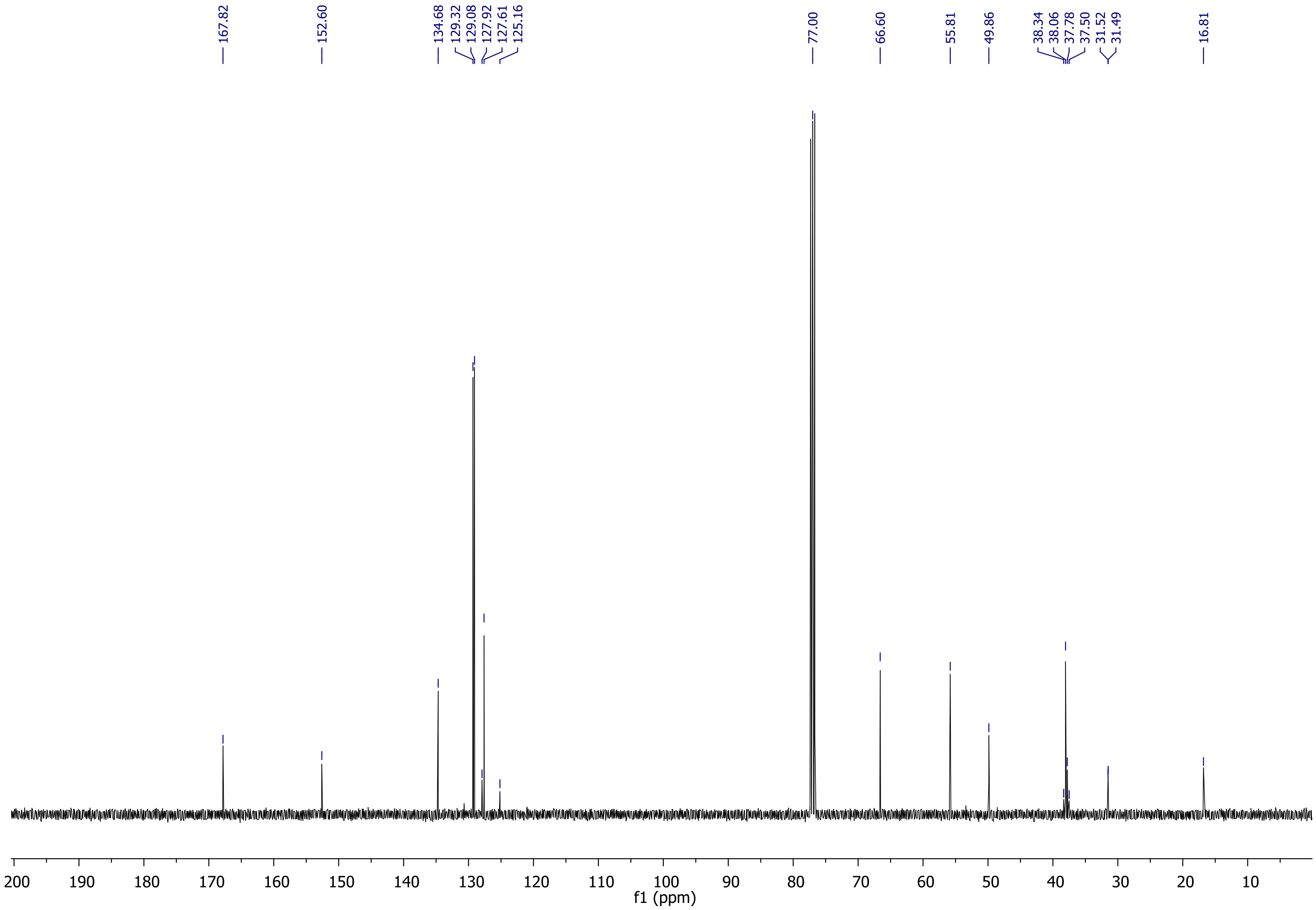

^{13}C NMR (101 MHz, $CDCl_3$)

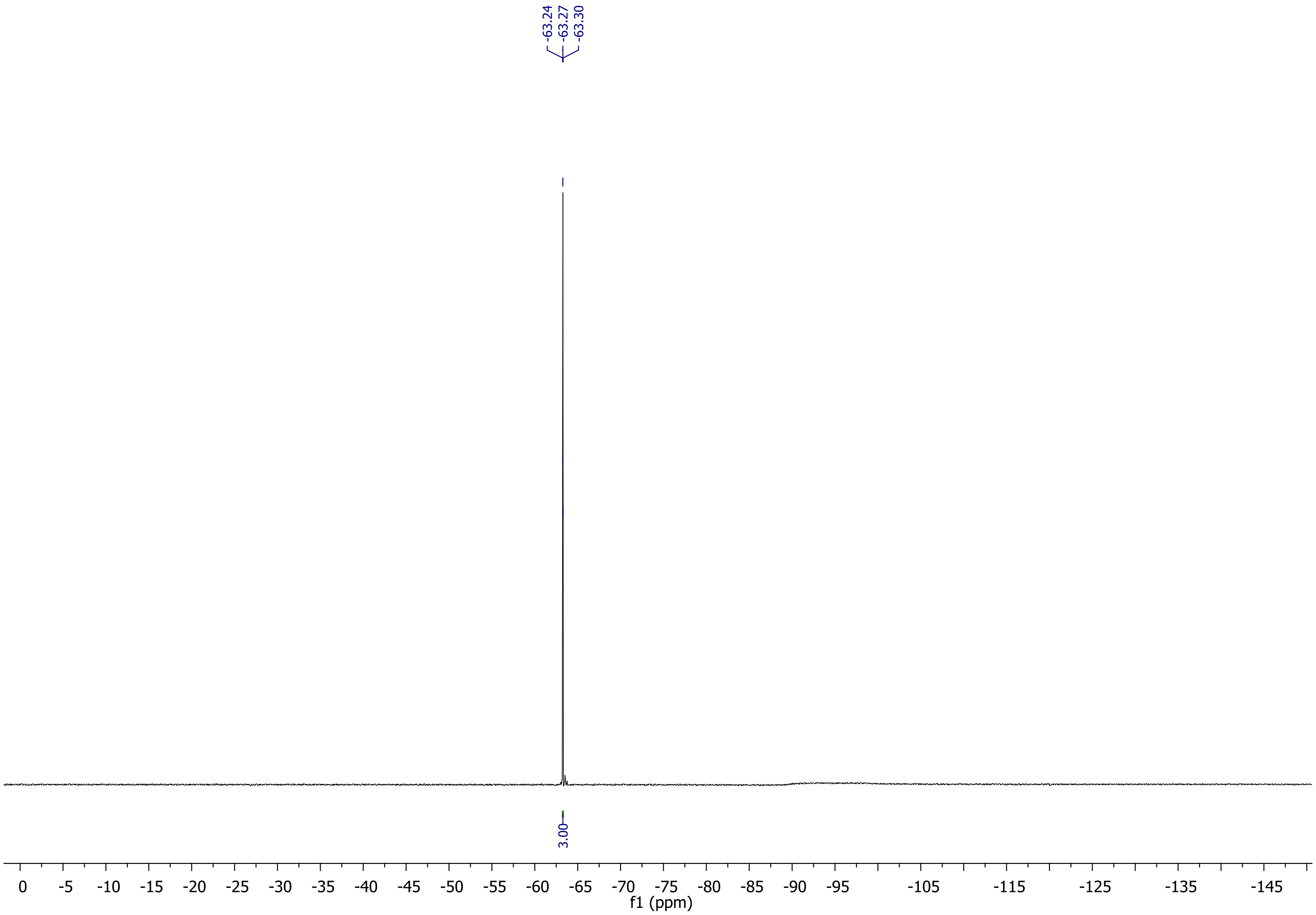

^{19}F NMR (376 MHz, $CDCl_3$)

Compound **6**

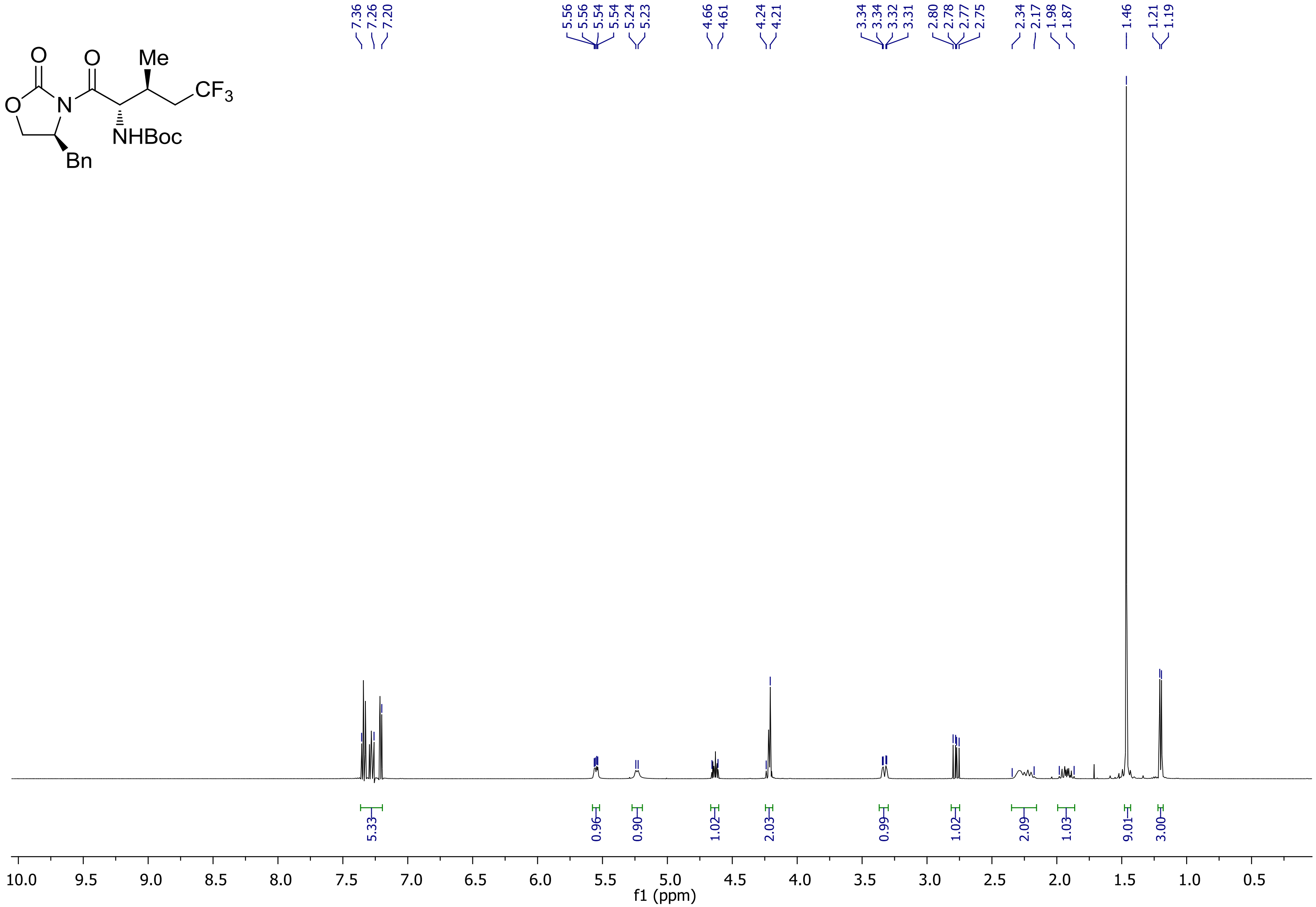

^{1}H NMR (500 MHz, $CDCl_3$)

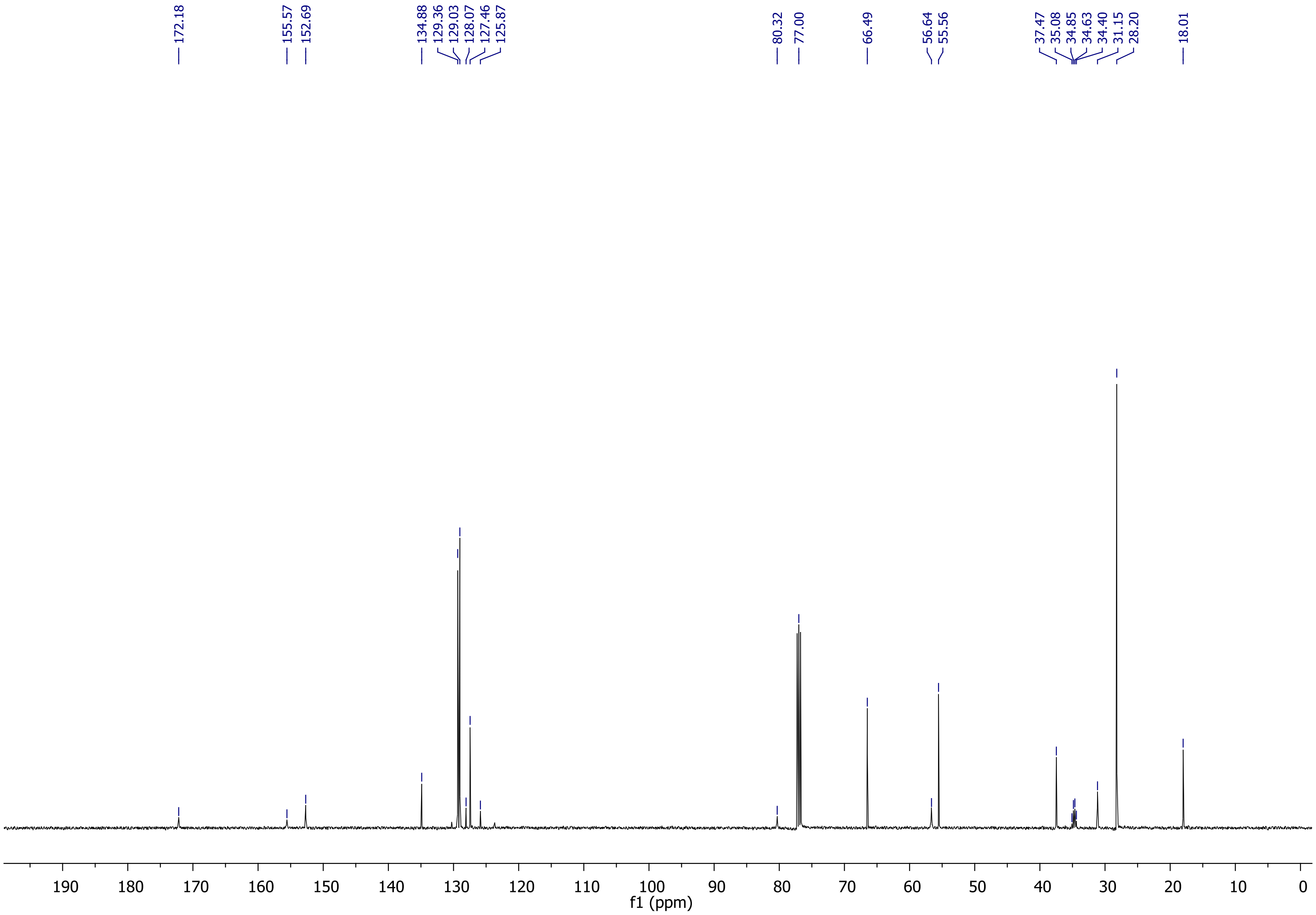

^{13}C NMR (126 MHz, $CDCl_3$)

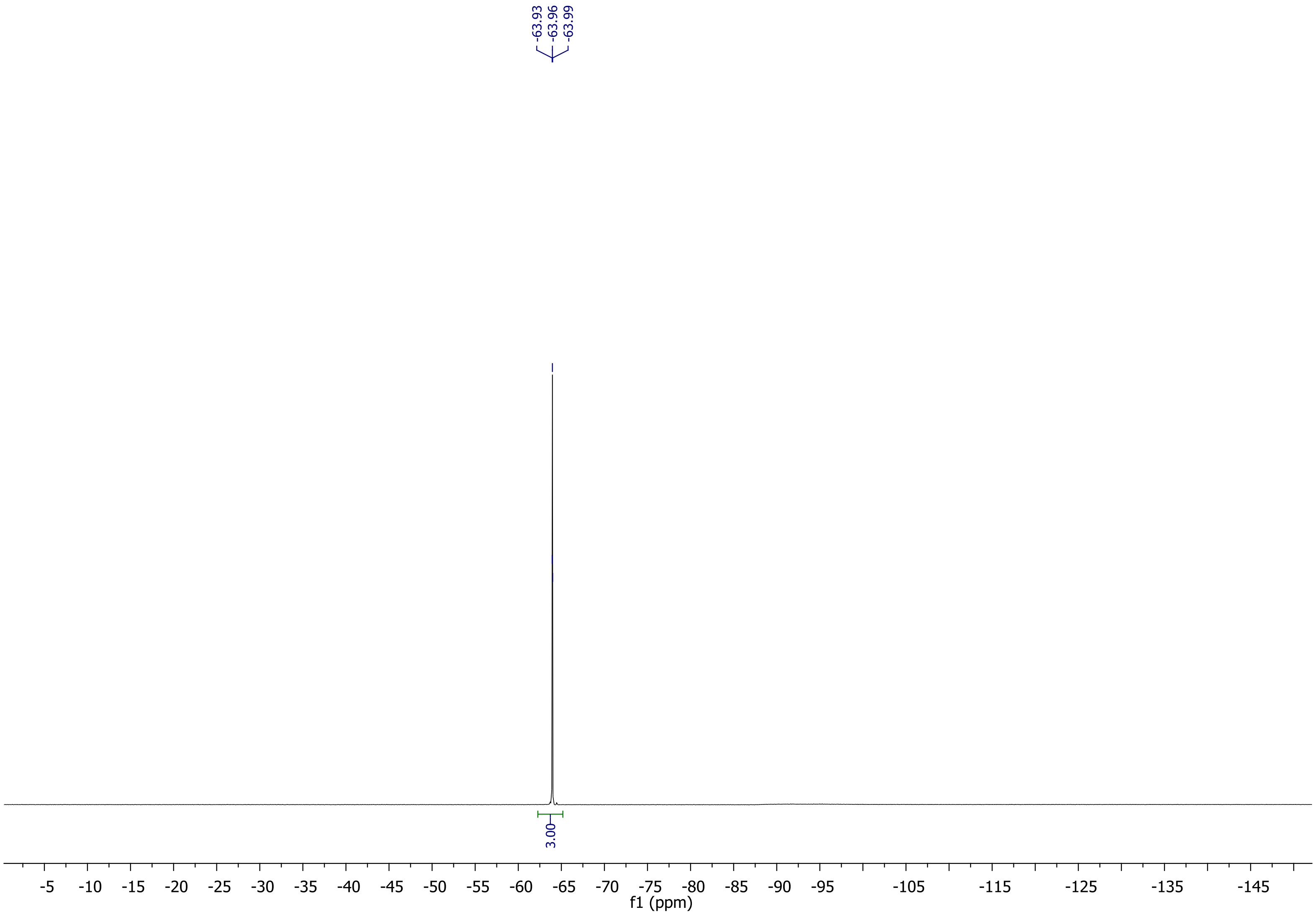

^{19}F NMR (376 MHz, $CDCl_3$)

Compound **9'**

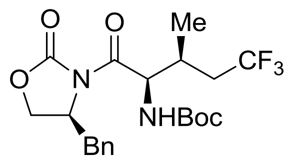

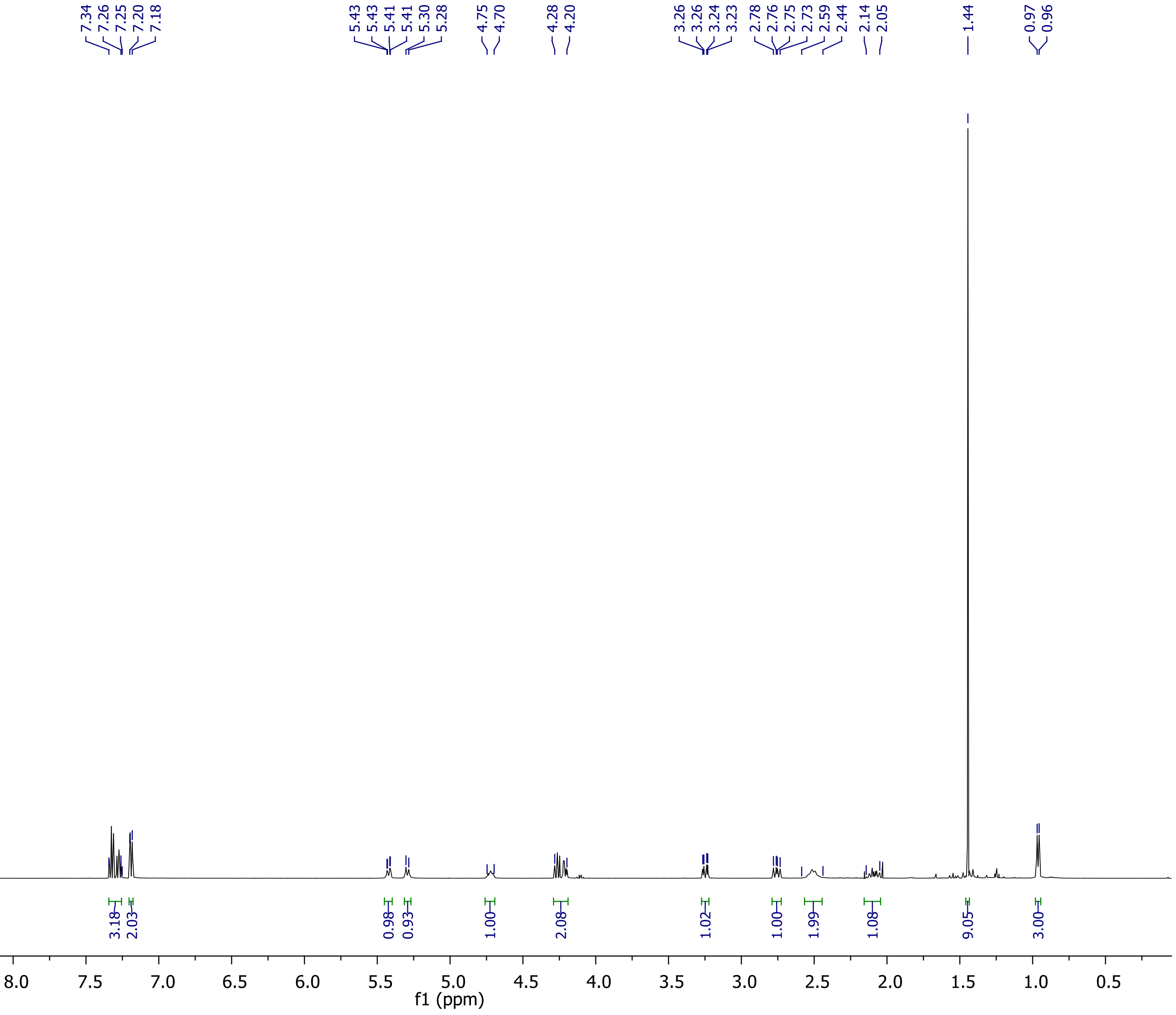

^{1}H NMR (500 MHz, $CDCl_3$)

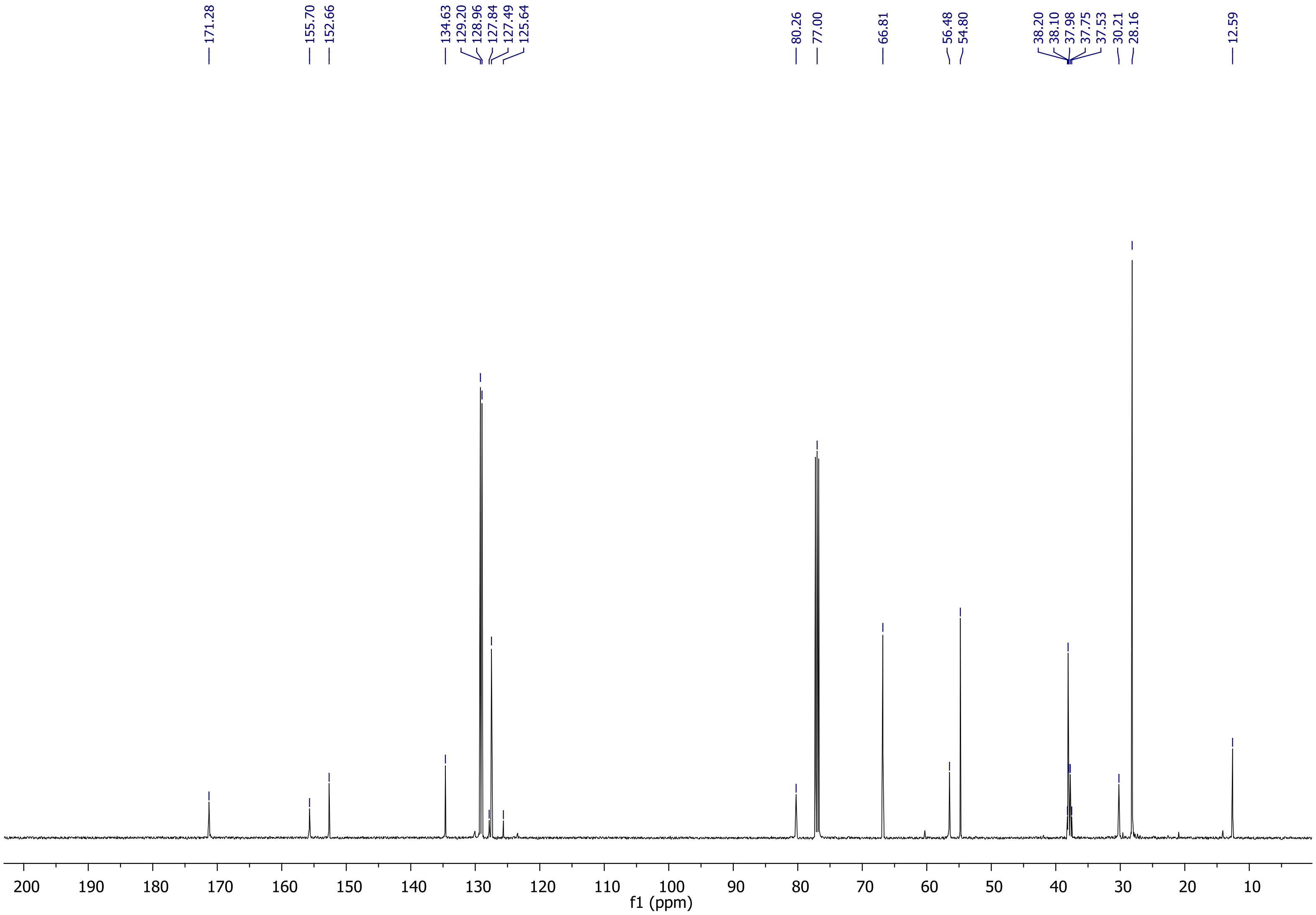

^{13}**C NMR** (126 MHz, $CDCl_3$)

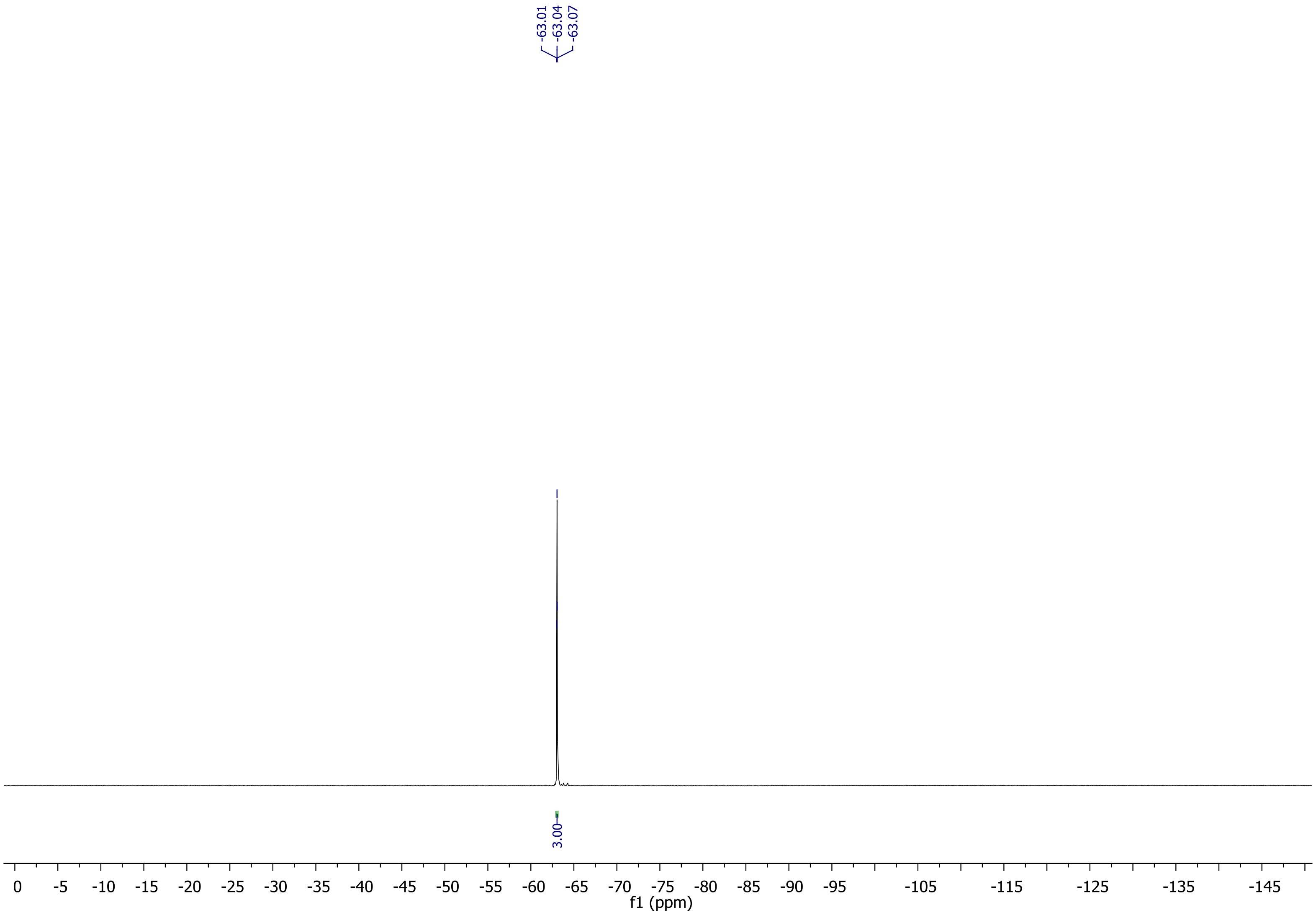

^{19}F NMR (376 MHz, $CDCl_3$)

Compound **7**

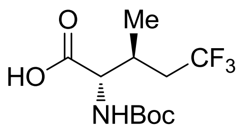

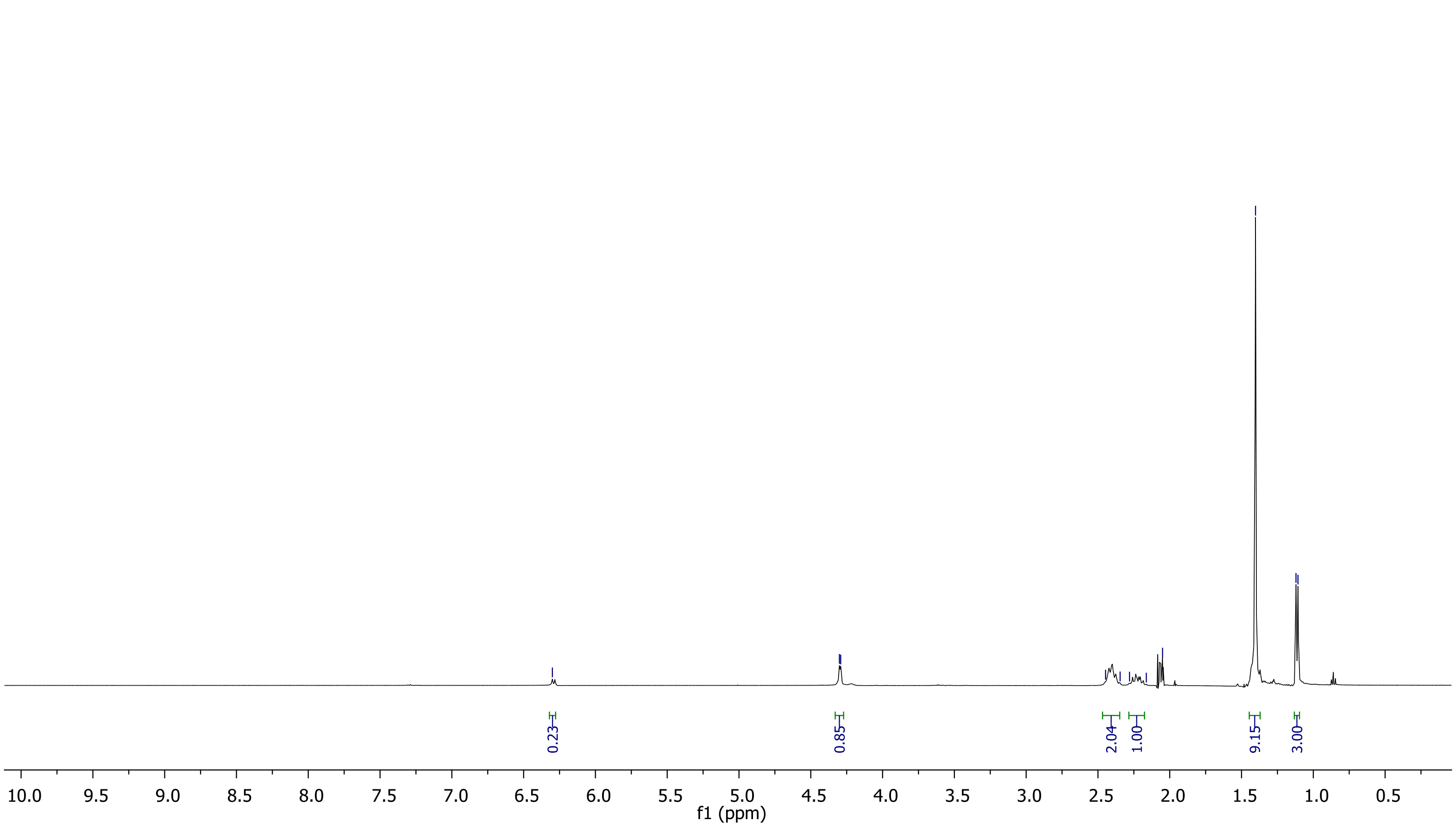

^{1}H NMR (500 MHz, $CDCl_3$)

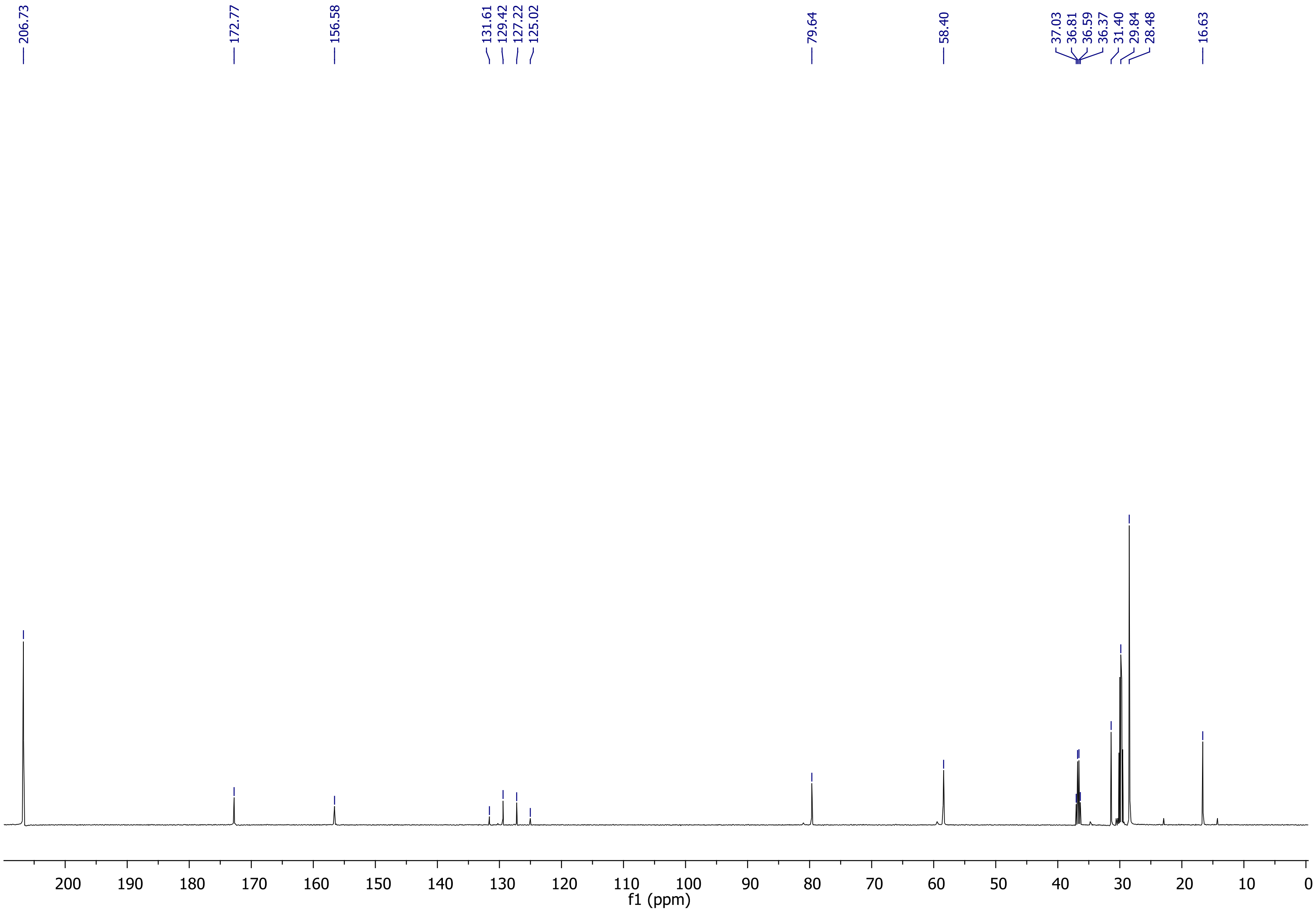

^{13}C NMR (126 MHz, $CDCl_3$)

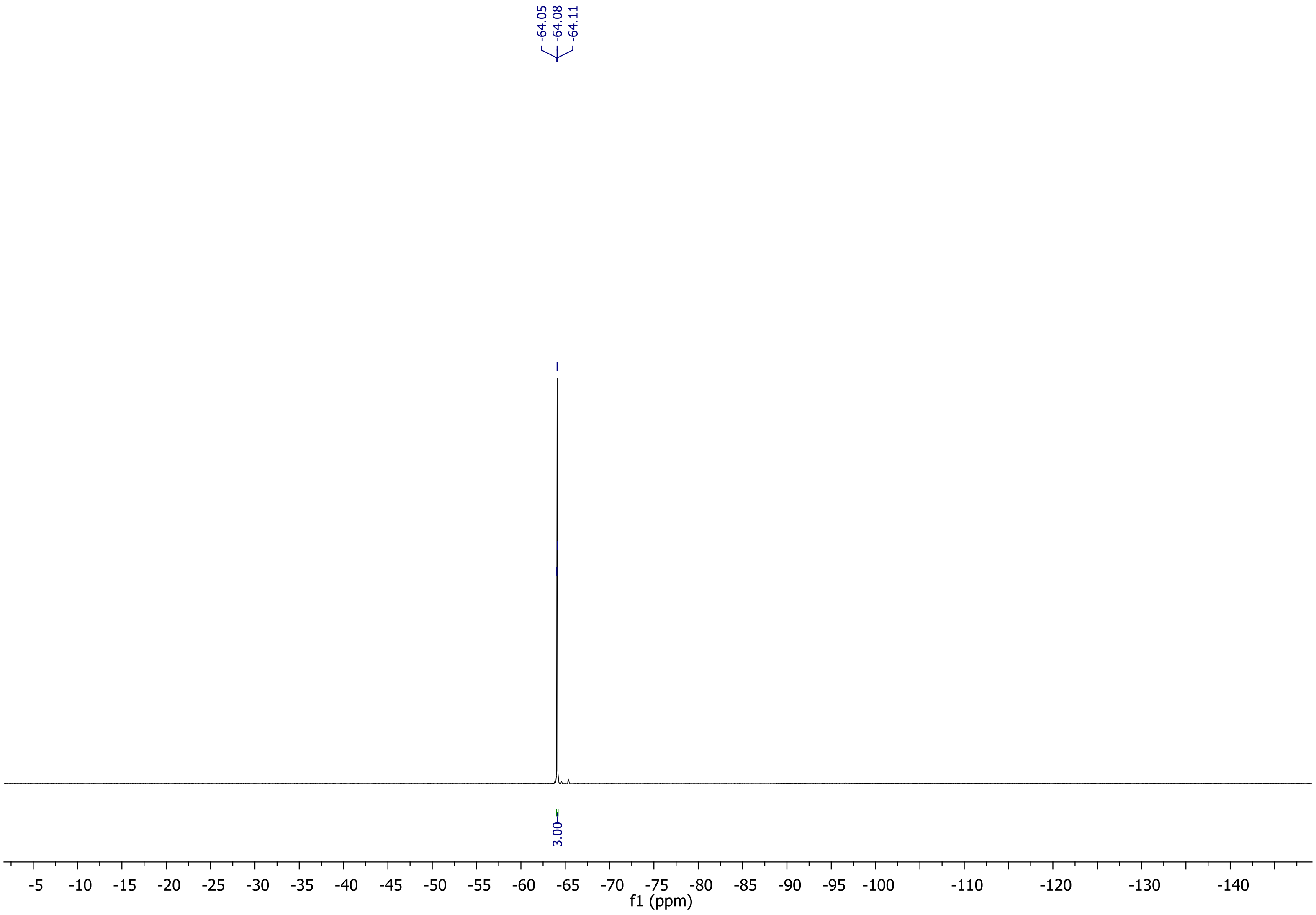

^{19}F NMR (376 MHz, $CDCl_3$)

Compound **10**

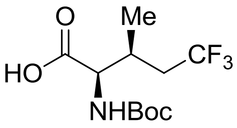

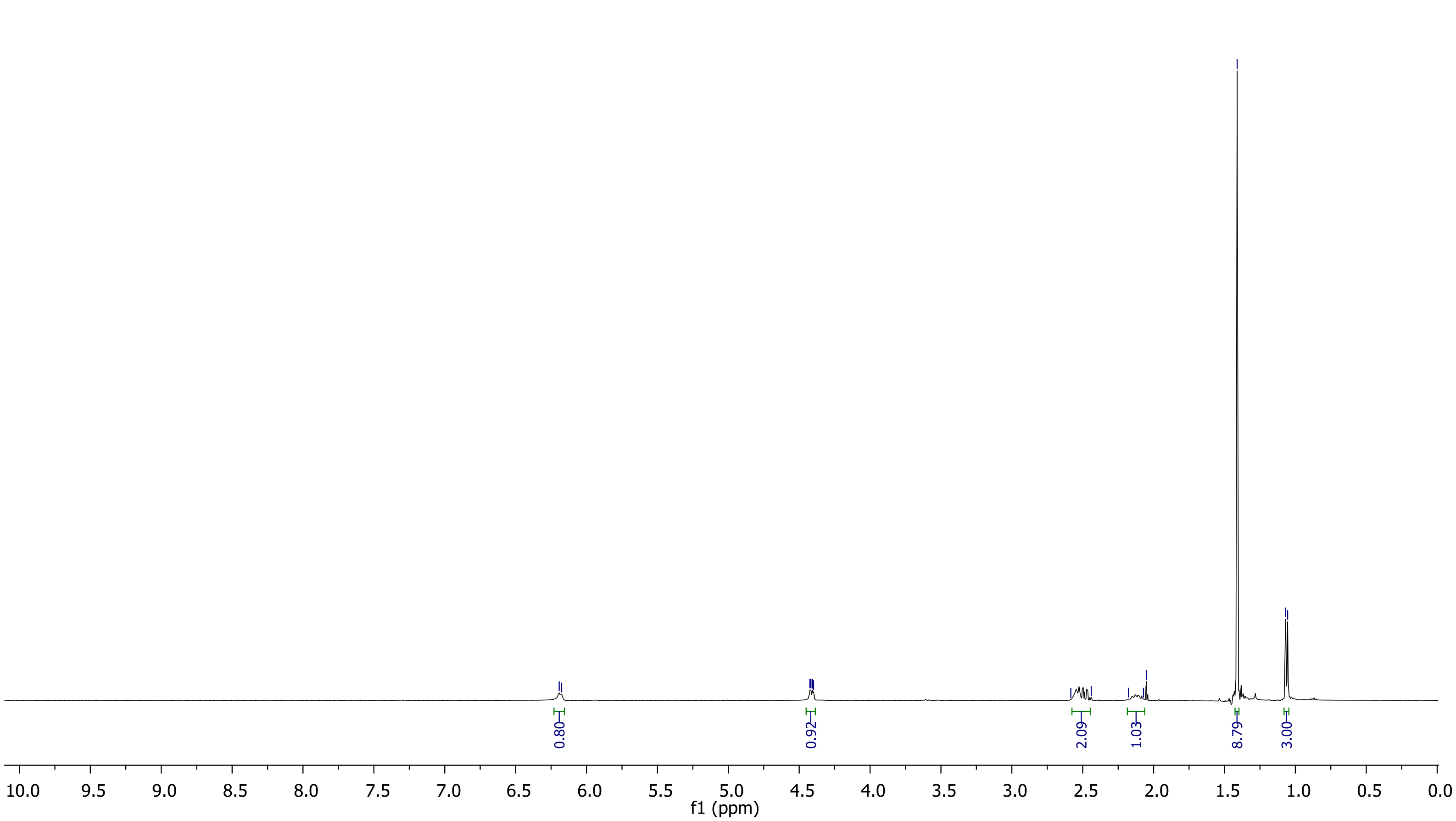

^{1}H NMR (500 MHz, $CDCl_3$)

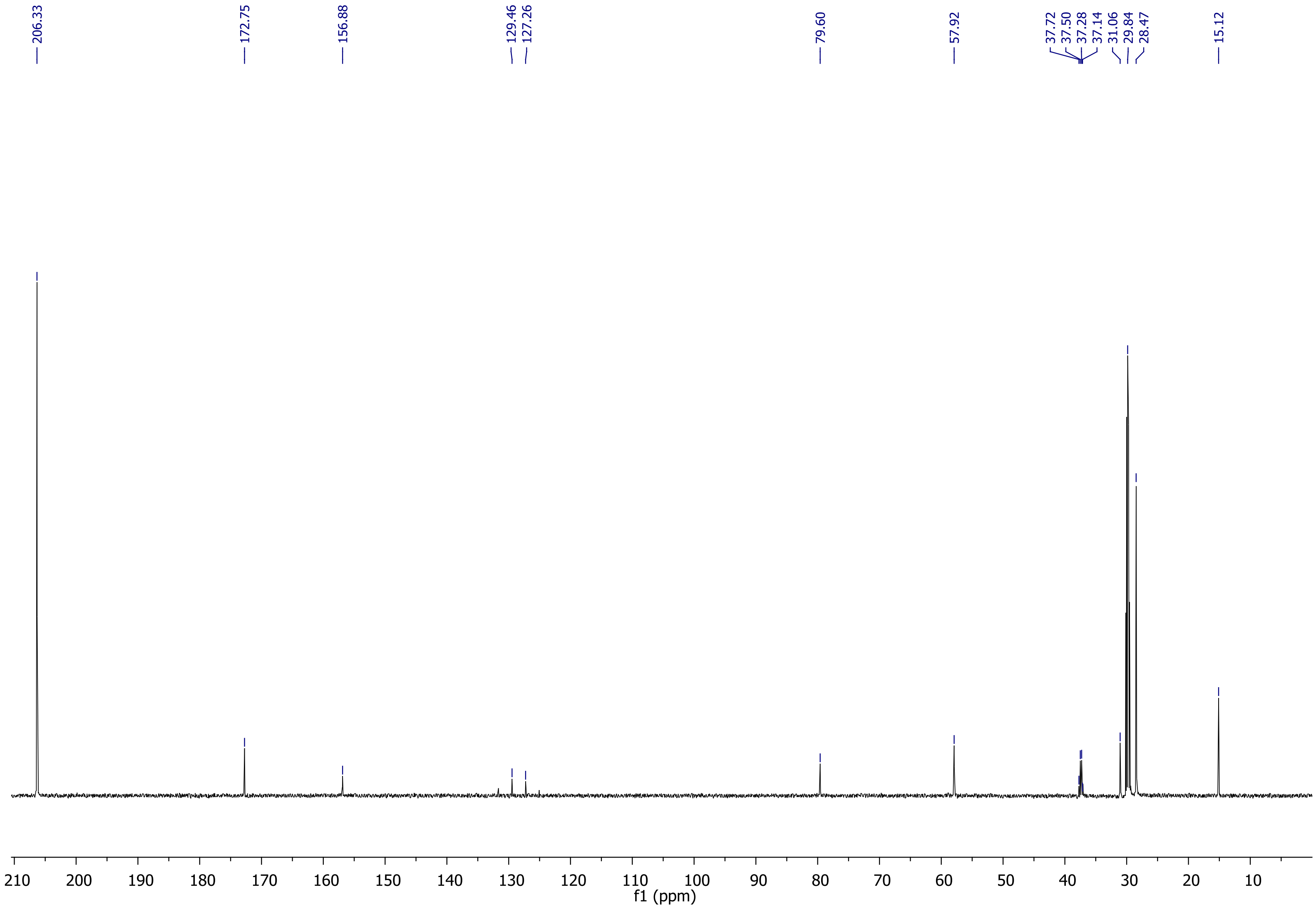

^{13}C NMR (126 MHz, $CDCl_3$)

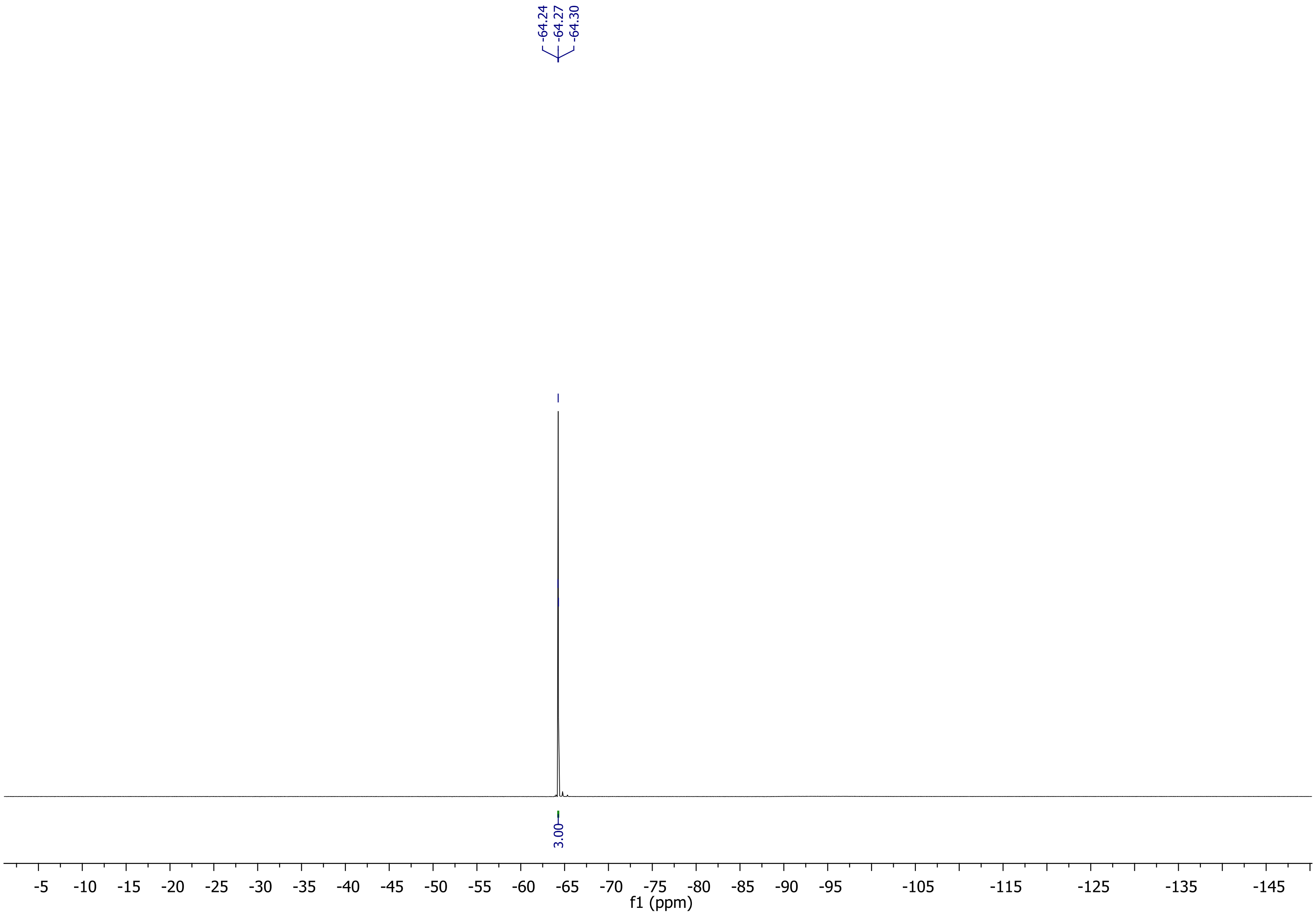

^{19}F NMR (376 MHz, $CDCl_3$)

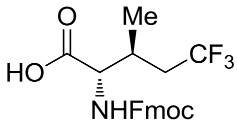

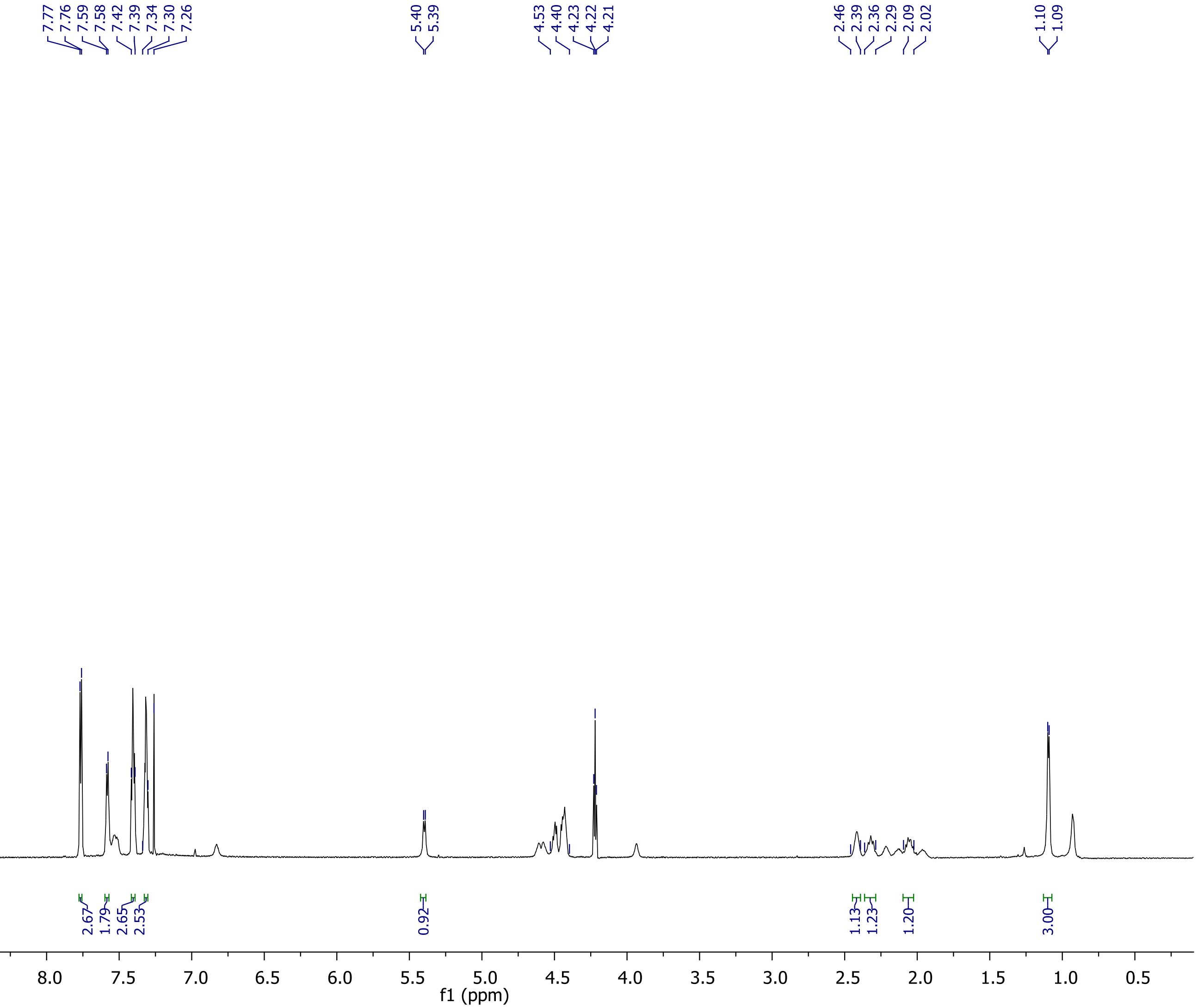

^{1}H NMR (700 MHz, $CDCl_3$)

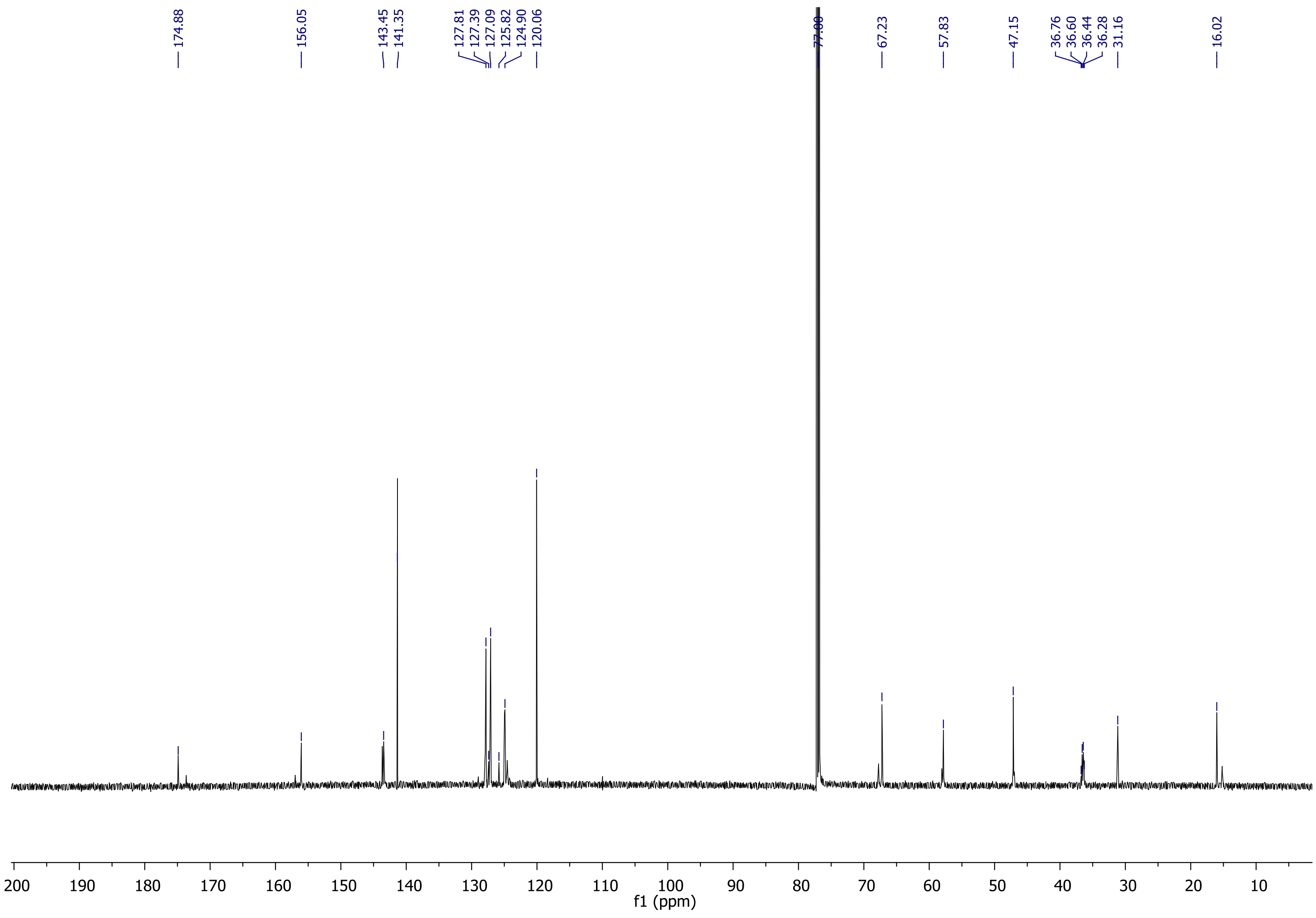

^{13}C NMR (176 MHz, $CDCl_3$)

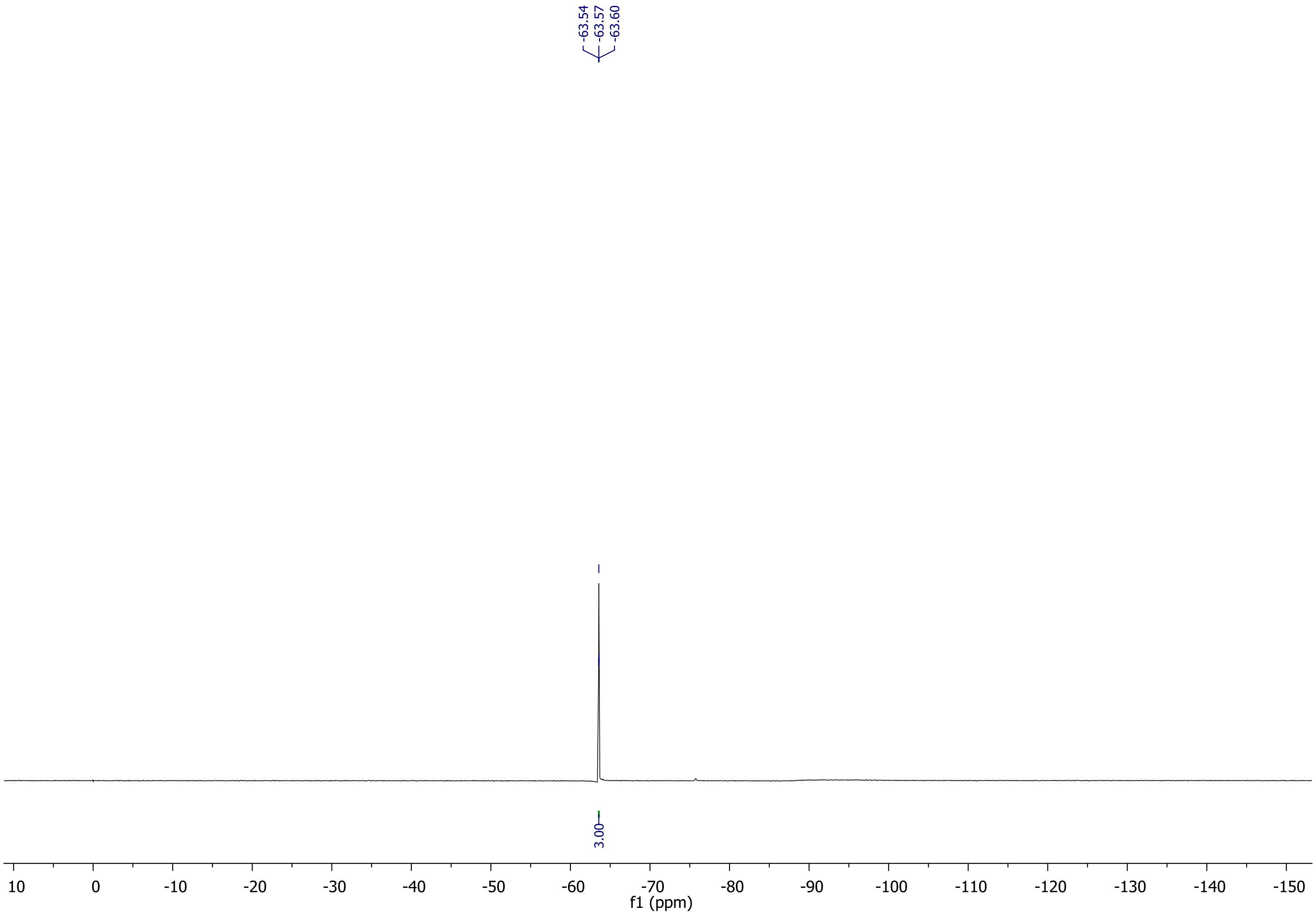

^{19}F NMR (376 MHz, $CDCl_3$)

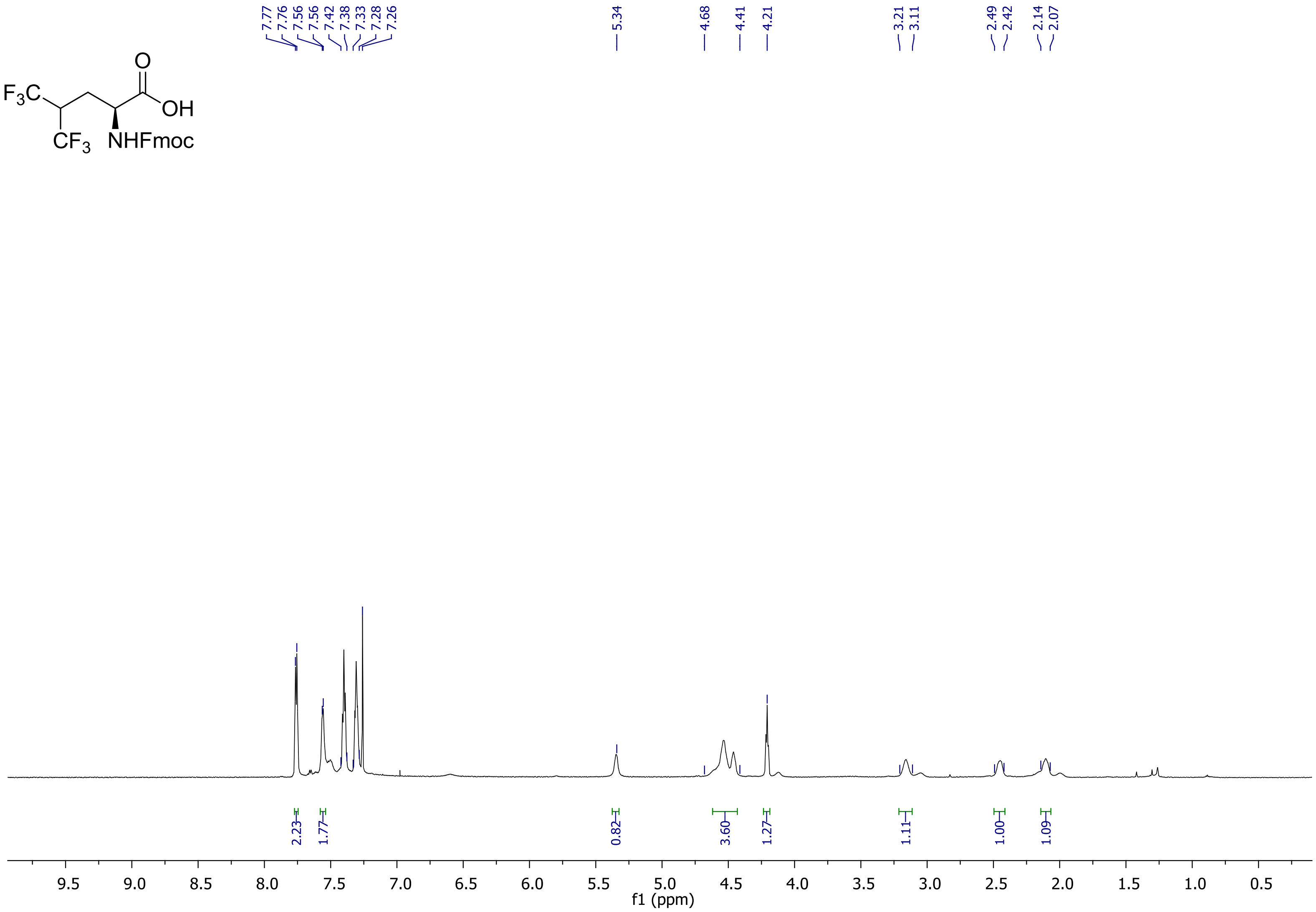

^{1}H NMR (700 MHz, $CDCl_3$)

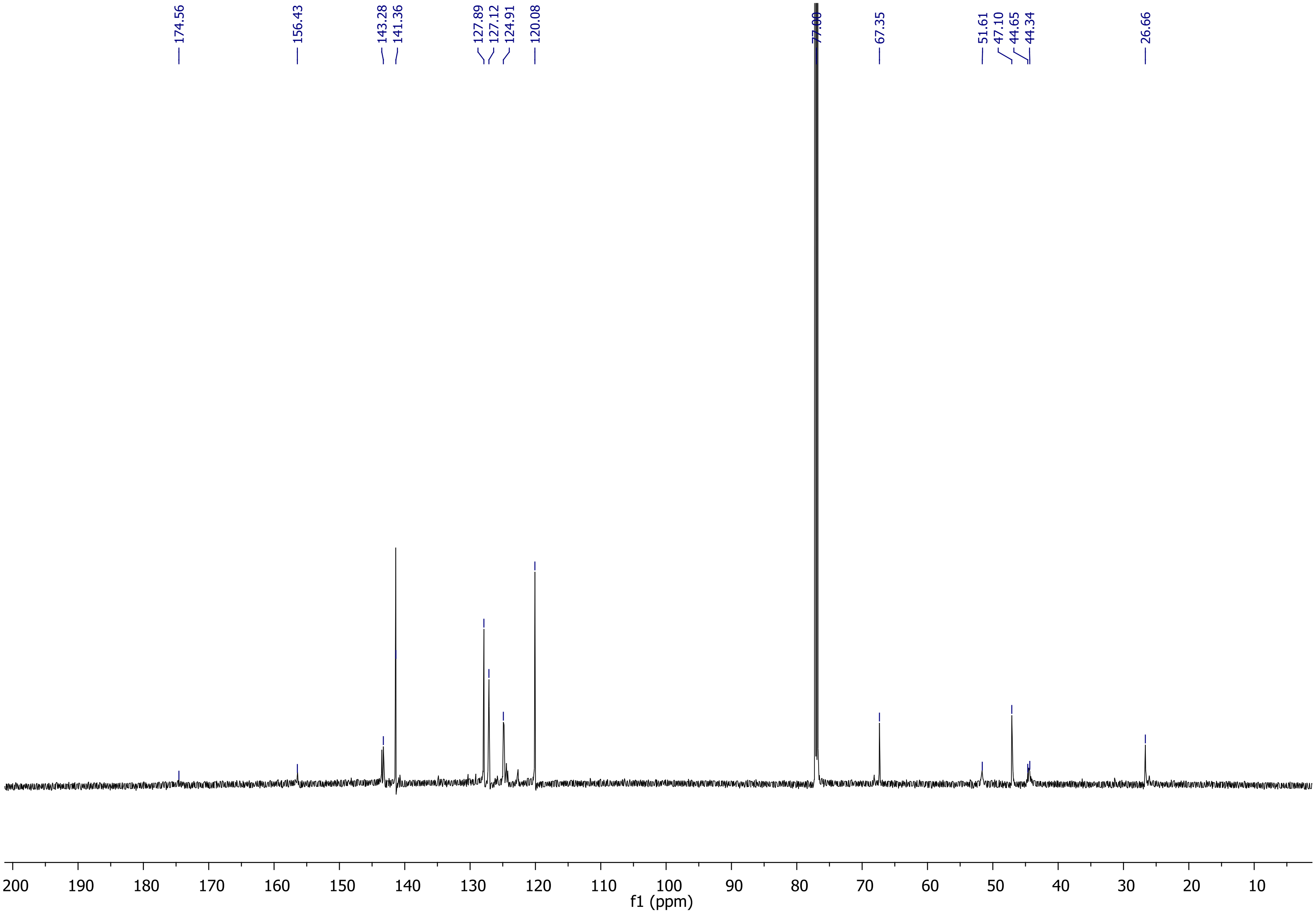

^{13}**C NMR** (176 MHz, $CDCl_3$)

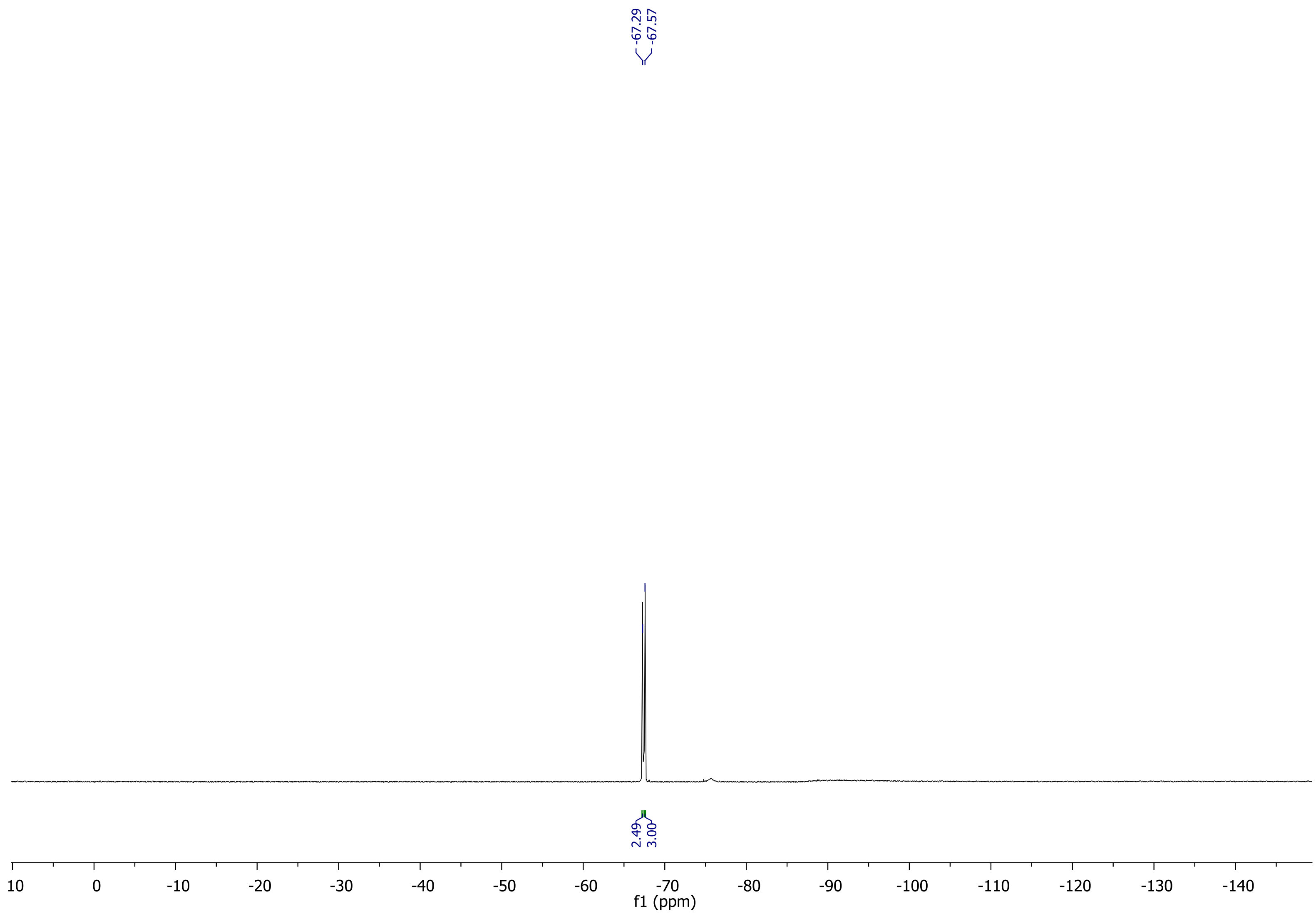

^{19}F NMR (376 MHz, $CDCl_3$)

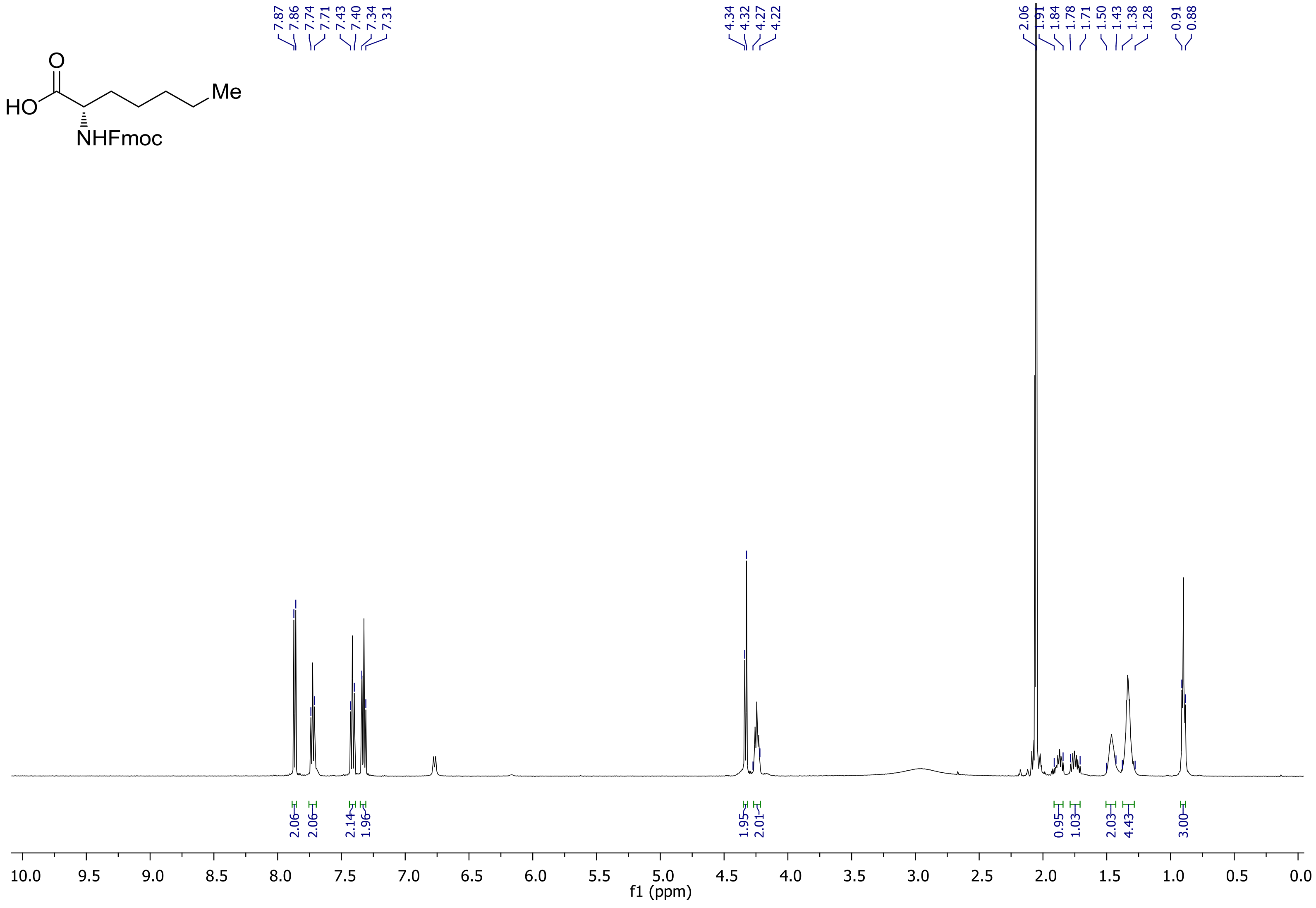

^{1}H NMR (500 MHz, C_3D_6O)

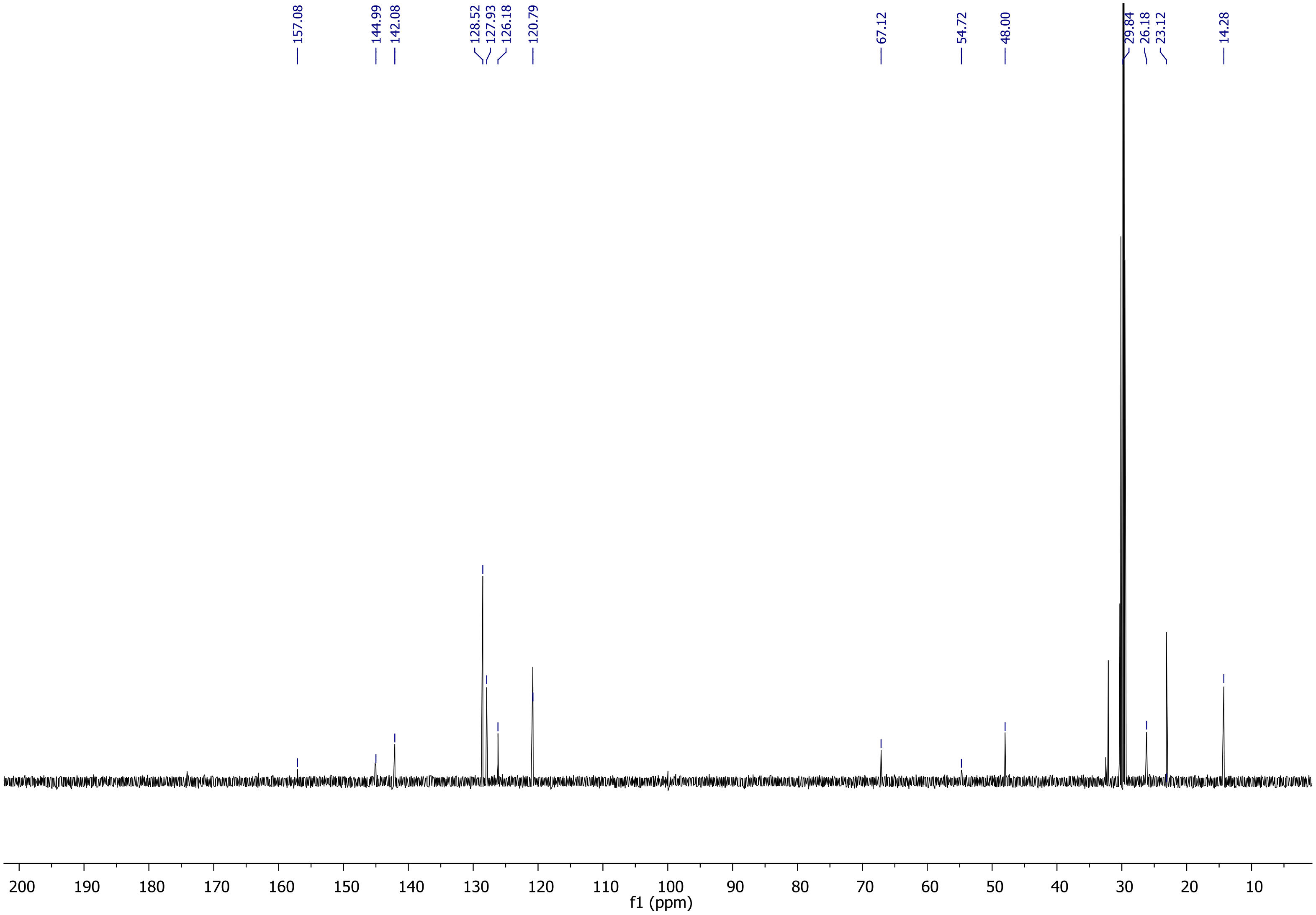

^{13}C NMR (126 MHz, $CDCl_3$)
